# Associations of the circulating levels of cytokines with risk of systemic sclerosis: a bidirectional Mendelian randomized study

**DOI:** 10.3389/fimmu.2024.1330560

**Published:** 2024-02-28

**Authors:** Zong Jiang, Xiaoling Yao, Weiya Lan, Fang Tang, Wukai Ma, Xueming Yao, Changming Chen, Xin Cai

**Affiliations:** ^1^ Second Clinical Medical College, Guizhou University of Traditional Chinese Medicine, Guiyang, China; ^2^ Department of Rheumatology and Immunology, The Second Affiliated Hospital of Guizhou University of Traditional Chinese Medicine, Guiyang, China; ^3^ Department of Rheumatology and Immunology, The First People’s Hospital Of Guiyang, Guiyang, China

**Keywords:** systemic sclerosis, cytokines, growth factor, Mendelian randomization, bidirectional

## Abstract

**Objective:**

Systemic sclerosis(SSc) remains unclear, studies suggest that inflammation may be linked to its pathogenesis. Hence, we conducted a bidirectional Mendelian randomization (MR) analysis to evaluate the association between cytokine and growth factor cycling levels and the risk of SSc onset.

**Methods:**

In our study, the instrumental variables(IVs) for circulating cytokines were sourced from the genome-wide association study (GWAS) dataset of 8293 Finnish individuals. The SSc data comprised 302 cases and 213145 controls, and was included in the GWAS dataset. We employed four methods for the MR analysis: MR Egger, Inverse variance weighted (IVW), Weighted medium, and Weighted Mode, with IVW being the primary analytical method. Sensitivity analyses were performed using heterogeneity testing, horizontal pleiotropy testing, and the Leave One Out (LOO) method. We also conducted a reverse MR analysis to determine any reverse causal relationship between SSc and circulating cytokines.

**Results:**

After Bonferroni correction, MR analysis revealed that the Interleukin-5 (IL-5) cycle level was associated with a reduced risk of SSc [odds ratio (OR)=0.48,95% confidence interval (CI): 0.27-0.84, P=0.01]. It also indicated that the Stem cell growth factor beta (SCGF-β) cycling level might elevate the risk of SSc (OR = 1.36, 95% CI: 1.01-1.83, P = 0.04). However, the reverse MR analysis did not establish a causal relationship between SSc and circulating cytokine levels. Additionally, sensitivity analysis outcomes affirm the reliability of our results.

**Conclusion:**

Our MR study suggests potential causal relationships between IL-5, SCGF-β, and the risk of SSc. Further research is essential to determine how IL-5 and SCGF-β influence the development of SSc.

## 1 Introduction

Systemic sclerosis (SSc) is a chronic and complex autoimmune disease characterized by immune abnormalities, fibrosis, excessive collagen deposition, and vascular lesions (1). Currently, the pathogenesis of SSc remains unclear, with treatments primarily focusing on inhibiting immune response, counteracting fibrosis, and promoting vasodilation. Although these methods have led to gradual improvements in the survival rates of SSc patients, the mortality rate remains considerably high. A study on the mortality rate of SSc in Italy revealed a standardized mortality rate for SSc as high as 2.8 (2), with interstitial lung disease, pulmonary arterial hypertension, and renal crisis being the primary causes of patient death (3, 4). However, the mechanism influencing the progression of SSc and resulting in patient death remain elusive. Understanding these pathogenic factors and mechanisms could slow disease progression, reduce mortality, and offer fresh perspectives.

Numerous studies have indicated that various risk factors, including genetic susceptibility, environmental factors, and immune abnormalities, might play significant roles in the pathogenesis of SSc. Recent research suggests that inflammation might be closely associated with the pathogenesis of SSc (5–8). Gough P analyzed 13 cytokines in 444 SSc patients and discovered significant variations in IL-6, IL-10, IL-17, IL-23, TNF-α, and TNF-γ levels compared to a healthy population (9). Research has also shown notable changes in the circulating levels of macrophages and monocytes in SSc patients when compared to a normal group (10). Subsequent studies identified that CD4+T cells play a pivotal role in the early stages of SSc pathogenesis, particularly Treg cell dysfunction, which might initiate inflammation. IL-6 and IL-17 can influence Treg plasticity, resulting in cellular imbalance, potentially triggering the onset of SSc (11–13). Treatment with the IL-6 inhibitor Tocilizumab has been shown to improve lung function, regulate macrophage activation, diminish inflammatory response, and delay skin deterioration (14, 15). However, debates continue regarding whether the autoimmune-mediated inflammatory response is active or if the inflammation-mediated immune disorder contributes to SSc onset. The potential causal relationship between inflammatory factors and SSc remains to be determined.

Mendelian randomization (MR) utilizes genome-wide association study (GWAS) data, comprising multiple single nucleotide polymorphisms (SNPs) genetic variation sites, as instrumental variables (IVs) to investigate causal relationships. Due to the random inheritance of genetic variations, MR can minimize confounding factors and reverse causal effects (16). It is now extensively applied in various disease exposure and outcome studies (17, 18). To date, no reports have addressed the causal relationship between cytokines and SSc, though GWAS on cytokines have been documented (19), facilitating our examination of the association between cytokines and SSc. Bidirectional MR conducts two dual sample MR analyses to determine any reverse causal relationship between exposure and outcome, enhancing our comprehension of the causal link between the two.

Therefore, using publicly available GWAS data, this study performed a bidirectional MR analysis with circulating levels of cyclines and SSc as either exposure factors or outcomes, offering fresh insights into the causal relationship between circulating levels of cytokines and SSc.

## 2 Materials and methods

### 2.1 Study design

**Figure 1:**
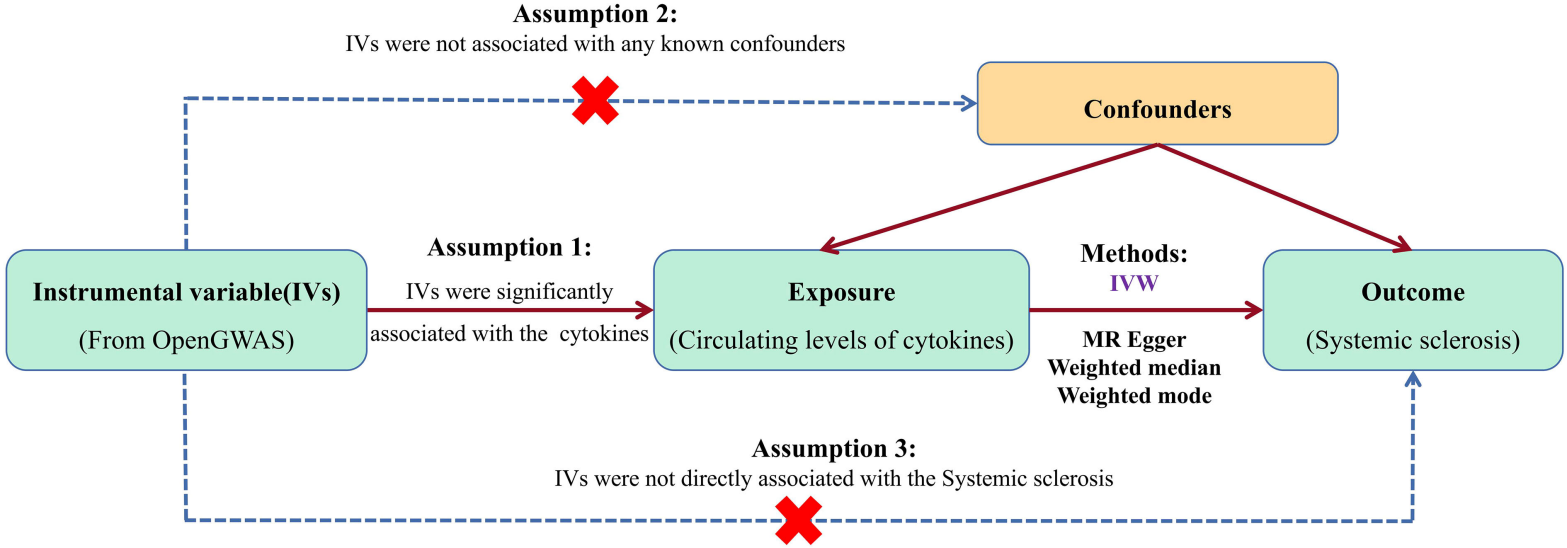
Data Source and Pre-processing.

**Figure 1** presents an overview of our dual sample MR study design. In line with MR research principles, MR research must fulfill three assumptions. First, the genetic variation chosen as IVs must be significantly associated with circulating cytokine levels. Second, the chosen IVs must be independent of any confounding factors. Lastly, IVs should only influence outcomes through exposure factors, not other biological pathways. This study draws upon data from publicly available databases; hence, no further ethical approval is necessary.

### 2.2 Circulating levels of cytokines

The GWAS summary statistical data on cytokine and growth factor cycling levels were sourced from 8,293 European individuals (19), with details provided in **Supplementary Table 1**. We selected the SNPs of IVs using the “TwoSampleMR” R software package (version 0.5.6). To ensure data reliability, we set the SNPs threshold at P<5×10^-8^ and conducted linkage disequilibrium analysis (LDA) for independence (r2 = 0.001 and kb=10000).

### 2.3 Systemic sclerosis

The IEU OpenGWAS database (https://gwas.mrcieu.ac.uk/) was used to download the SSc-related dataset (finn b-M13_SYSTSLCE). Finn-b-M13_ SYSTSLCE contains 302 case samples, 213145 control samples, and 16380454 SNP samples (**Supplementary Table 1**). We also performed IVs screening using the “TwoSampleMR” R software package (P<5×10^-8^, r2 = 0.001 and kb=10000) to investigate the reverse causal relationship between.

### 2.4 Statistical analyses

Due to the fact that the SNP loci of both GWAS studies were from the European population, there may be bias in the results. Therefore, we calculated the sample overlap error rate, which is between 0% - 9.2% (error rate <10%), and will not have a significant impact on the results (20).

After extracting SNP information for cytokines, we first computed the F-statistic for cytokines to assess the strength of the IVs (21). The F-statistic range for IVs in this study varied from 11 to 789, suggesting adequate strength of the IVs (22) (**Supplementary Table 2**). Inverse variance weighting (IVW) is viewed as a more precise and unbiased analytical method, so we employed IVW as the primary method to compute the odds ratio (OR) for interpreting risk or protective factors in the findings. We also incorporated three other MR analysis methods (MR Egger, weighted median, and weighted mode) to address potential confounding factors (23).

To achieve objective and dependable statistical outcomes, we applied the Bonferroni method to adjust for multiple comparisons. Given the number of cytokines, the Bonferroni correction set the statistical significance level at P<0.0012 (0.05/41), treating P-values between 0.0012 and 0.05 as suggesting potential causal associations (24).

In addition, we executed further MR analyses to investigate the causal links between cytokines and potential confounding factors influencing SSc. These factors comprised: Drugs used in diabetes, Antithrombotic agents, Beta blocking agents, Calcium channel blockers, HMG CoA reductase inhibitors, Immunosuppressants, Anti-inflammatory and antirheumatic products, non-steroids, Drugs affecting bone structure and mineralization, Glucocorticoids Detailed information about the data sources is available in **Supplementary Table 1**.

To evaluate the robustness of our findings, we undertook heterogeneity testing, horizontal pleiotropy testing, and a Leave One Out (LOO) sensitivity analysis. For heterogeneity testing, a Q value exceeding 0.05 implies an absence of heterogeneity. An MR Egger intercept with P<0.05 is deemed statistically significant (25), signaling the existence of horizontal pleiotropy. We further explored the presence of horizontal pleiotropy and reassessed after outlier removal using the MR PRESSO test. LOO evaluates the influence of each SNP for potential outliers. All MR analyses were executed with the “TwoSampleMR” and “MRPRESSO” software packages in R (version 4.2.0) (26–28).

## 3 Results

Initially, using SNPs with P<5×10^-8^, r2 = 0.001, and kb=10000 as IVs, no significant correlation was found between cytokines and SSc. Adjusting the criteria to P<5×10^-6^, r2 = 0.001, and kb=10000 for MR analysis, 330 SNPs associated with 41 cytokines were included as the IVs for this study. The F-statistic for the IVs was >10, signifying the robustness of the selected IVs (**Supplementary Table 2**). When SSc as an exposure factor and cytokine levels, ultimately selecting 7 SNPs (**Supplementary Table 3**).

**Figure 2:**
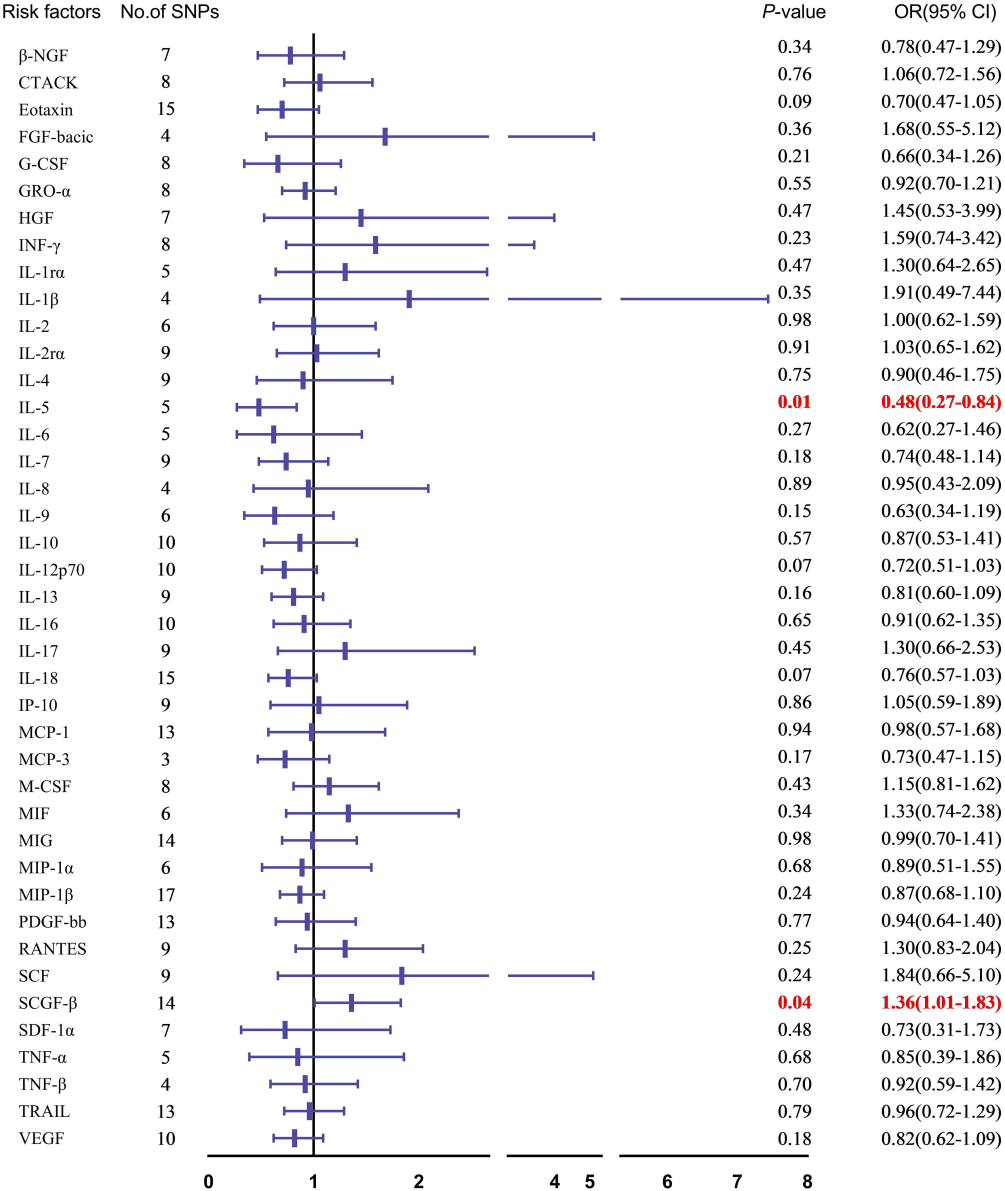
Mendelian randomization analysis of the association between circulating cytokines and SSc risk forest map.

In the MR analysis of cytokines and SSc, our primary focus was on the results from the IVW method. Post-Bonferroni correction, only two cytokines, IL-5 and SCGF-β, demonstrated an association with SSc risk (**Figure 2**). Elevated genetic circulating levels of IL-5 were inversely related to the risk of SSc (OR=0.48, 95% CI: 0.27-0.84, P=0.010). Conversely, higher circulating levels of SCGF-β were positively associated with the risk of SSc (OR=1.36,95% CI: 1.01-1.83, P=0.041) (**Figure 2**).

**Figure 3:**
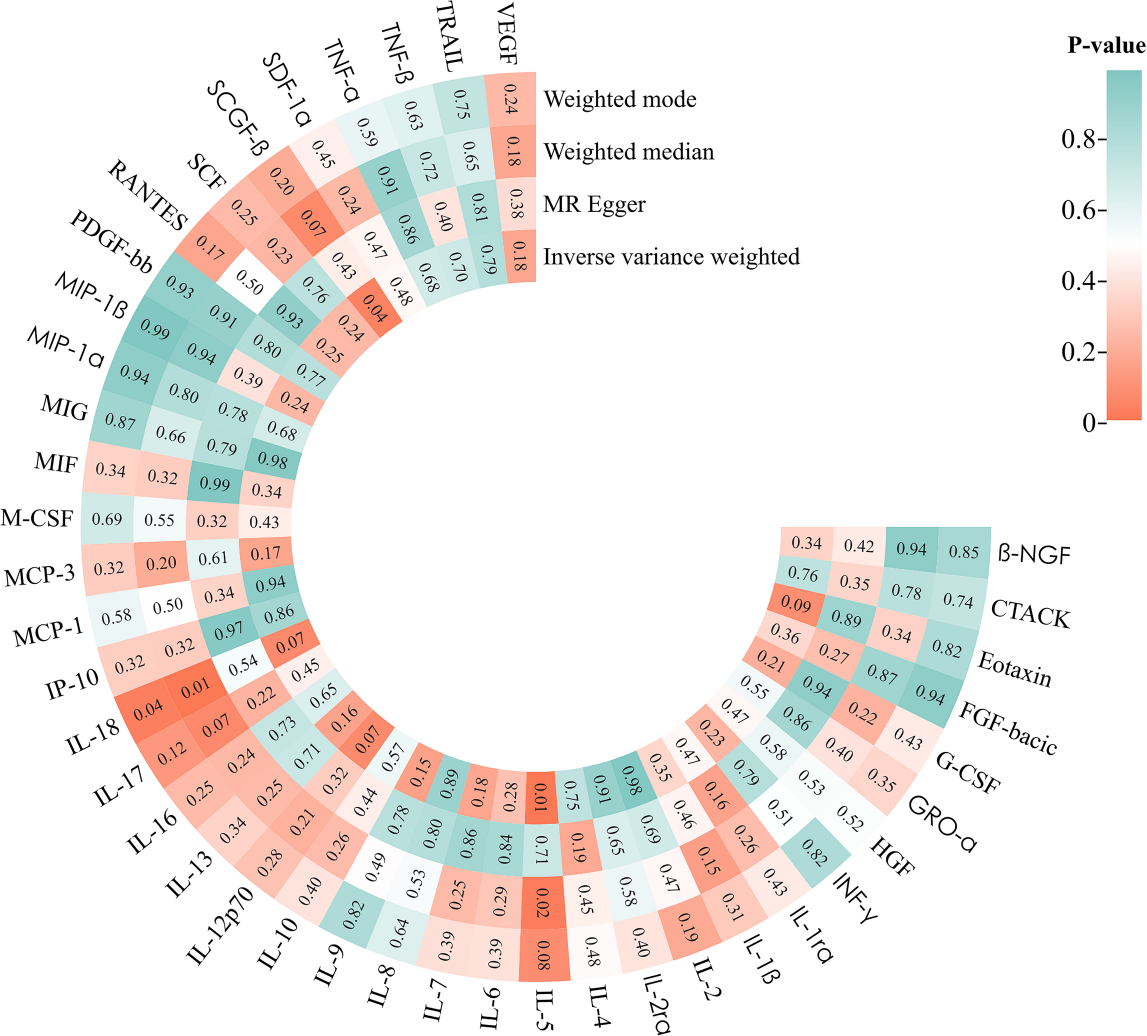
Mendelian Randomized Analysis Heat Map.

In MR analysis using the MR Egger, Weighted Median, and Weighted Mode methods, barring the discrepancy in IL-18 results compared to IVW, the trends for other cytokines were largely congruent with IVW, underscoring the dependability of the analysis results (**Figure 3**).

In order to avoid the influence of confounding factors, when examining the causal relationship between cytokines and SSc, no significant confounding factors were found by adjusting commonly used drugs for treating SSc such as immunosuppressants, glucocorticoids, and Calcium channel blockers. There was a negative causal relationship between IL-5 and SSc, and SCGF- β There is a positive causal relationship with SSc (**Table 1**).

**Table 1:** Analysis of possible confounding factors between cytokines and SSC.

Confounding factors	*P*-value	OR (95% CI)	*P*-value	OR (95% CI)
	IL-5 (*P*=0.010)		SCGF-β (*P*=0.041)	
Drugs used in diabetes	0.110	0.632(0.360-1.109)	0.236	1.221(0.878-1.697)
Antithrombotic agents	0.035	0.481(0.244-0.949)	0.045	1.432(1.007-2.036)
Beta blocking agents	0.109	0.555(0.270-1.139)	0.131	1.301(0.924-1.833)
Calcium channel blockers	0.076	0.424(0.165-1.095)	0.152	1.268(0.916-1.757)
HMG CoA reductase inhibitors	0.154	0.550(0.242-1.250)	0.160	1.306(0.900-1.896)
Immunosuppressants	0.029	0.384(0.162-0.909)	0.056	1.353(0.992-1.846)
Anti-inflammatory and antirheumatic products, non-steroids	0.026	0.377(0.159-0.891)	0.072	1.332(0.975-1.821)
Drugs affecting bone structure and mineralization	0.031	0.375(0.154-0.914)	0.099	1.312(0.950-1.811)
Glucocorticoids	0.013	0.470(0.258-0.854)	0.057	1.360(0.991-1.865)

To ensure result reliability, we conducted sensitivity analysis. Firstly, in the statistical analysis of cytokine heterogeneity, IL-5 and SCGF-β The Q>0.05 indicates the absence of heterogeneity (**Supplementary Table 4**). Then, in the horizontal pleiotropy test, IL-5 (P=0.509) did not exhibit horizontal pleiotropy, while SCGF-β (P=0.038) Preliminary statistics showed horizontal pleiotropy, and we further analyzed SCGF through MR presso analysis-β (P=0.521), confirming the absence of confounding factors in this study (P=0.328) (**Supplementary Table 5**). Afterwards, the LOO method indicated no bias points (**Supplementary Figure 1**). In conclusion, our findings are dependable, suggesting a causal relationship between cytokines IL-5 and SCGF-β and SSc.

To elucidate the precise causal link between circulating cytokines and SSc, we embarked on a reverse MR analysis, positioning SSc as the exposure factor and cytokine levels as the outcome. Employing the same selection criteria, 7 independent SNPs were identified (**Supplementary Table 3**). In the IVW model, no notable connection was discerned between SSc and cytokine levels (P>0.05) (**Supplementary Table 6**). Similarly, the other three models yielded consistent outcomes. Furthermore, the sensitivity analysis confirmed the reliability of our findings (**Supplementary Figure 2**; **Supplementary Tables 7**, **8**).

## 4 Discussion

In this study, we used GWAS data of genetic variation for bidirectional two-sample MR analysis to examine the potential causal relationship between multiple cytokine cycle levels and SSc, demonstrating strong genetic evidence. We found a causal relationship between IL-5 and SCGF-β circulation levels and SSc.

The imbalance of the immune system, especially Th1/Th2 cytokines, is central to the pathogenesis of SSc (29). IL-5 is a principal member of Th2 cytokines and a key driver of the Th2 pathway, playing a significant role in SSc (11). Concurrently, studies indicate that IL-5 can activate various signaling pathways, mediate the recruitment, maturation, differentiation, and proliferation of eosinophils, and promote B cell differentiation, activating B cells to produce immunoglobulins and partake in immune responses (30, 31). Previously, only one study noted an increase in eosinophils in SSc patients, especially in men without vascular disease (32). This might relate to our finding of a causal link between IL-5 and SSc and the IL-5 mediated eosinophil immune response. Another test that included 30 SSc patients and 80 normal control serum samples found reduced IL-5 expression in SSc (33). This corresponds to our MR analysis, which determined that lower circulating levels of IL-5 correlate with a higher risk of SSc. Additionally, in studies of SSc combined with interstitial lung disease (ILD), serum Th2 cytokine levels in SSc patients were found to be higher than in the control group. A linear correlation was also observed between ground-glass-like lesions, IL-5, and peripheral blood eosinophils, affirming that the Th2-mediated inflammatory response might be pivotal in the onset of SSc ILD (34). As IL-5 is the primary driver of Th2, this suggests that IL-5 circulation levels might be intimately tied to the onset of SSc. Regarding IL-5 targeted therapeutic drugs, the most notable, Mepolizumab, was approved in 2015. Studies have shown that Mepolizumab can mitigate eosinophil-mediated inflammatory responses while dampening the immune response (35). Therefore, further research is essential to pinpoint the precise role of IL-5 in SSc and offer fresh insights for the clinical targeted treatment of SSc.

Stem cell growth factor (SCGF) is a growth factor derived from artificial blood progenitor cells that promotes cell activation and activation of human stem cells and immune cells in the body. It has two subtypes, α and β (36). Currently, most research focuses on SCGF-β. Research has found that SCGF-β participates in the inflammatory mechanisms of metabolic disorders related to obesity (37), cardiovascular diseases (38), reproductive system inflammatory lesions (39), cerebral nervous system (40), lumbar disc herniation (41), all closely related to the severity of inflammation. However, there is limited evidence of a relationship between SCGF-β and SSc. Only a few clinical case-control observations exist, and in them, the SCGF-β levels in SSc patients were found to be significantly higher than in normal individuals (36). It’s worth noting that this aligns with our MR analysis results, suggesting a positive causal relationship between SCGF-β circulation levels and SSc. Conversely, in a study targeting the activation of endothelial mitogen-activated protein kinase (MAPK) in a mouse model, which activates inflammatory responses through Nuclear factor kB (NF-kB) leading to functional defects in vascular niches and hematopoietic stem cells, it was found that SCGF infusion can inhibit inflammation, restore vascular integrity, and address functional defects in hematopoietic stem cells (42). This differs from earlier reports suggesting SCGF-β as a pro-inflammatory factor (43). Therefore, due to these inconsistent findings, further research, especially mechanism studies, is required to determine the exact role of SCGF-β circulation levels in the development of SSc. To our knowledge, this is the first bidirectional MR study on the causal relationship between cytokine cycling levels and SSc. To ensure the reliability of our findings, we also conducted a reverse MR analysis to further exclude confounding factors.

From a genetic perspective, our research has found a causal relationship between SSc and cytokines. Currently, GWAS data on SSc already exists, but due to the lack of original data, we are unable to conduct larger sample studies (44). In addition, our research has other limitations: First, the cytokine IV levels in the study population are all derived from Finns, so further studies are needed to determine if these results are applicable to other ethnicities. Second, in line with several other studies (45–47), when we used P<5×10^-8^, we didn’t find any available IVs and had to relax the threshold to P<5×10^-6^, potentially introducing false positives. Yet, the F-statistic for all IVs was >10, suggesting that the risk of bias and false positives is minimal. Lastly, after a Bonferroni correction, only IL-5 and SCGF-β were identified as potential risks associated with SSc, with no other significant correlations between cytokines and SSc risk. Therefore, further validation of the potential association between cytokines and SSc in more GWAS studies across different races is needed. Nevertheless, even after adjusting for confounding factors such as commonly used immunosuppressants and glucocorticoids, we found from a genetic perspective that IL-5 is associated with SCGF- β There may be a causal relationship with SSc.

## Data availability statement

The original contributions presented in the study are included in the article/**Supplementary Material**. Further inquiries can be directed to the corresponding authors.

## Ethics statement

The requirement of ethical approval was waived by The Second Affiliated Hospital of Guizhou University of Traditional Chinese Medicine for the studies involving humans because our data comes from reanalysis of public databases, so no additional ethical approval is required. The studies were conducted in accordance with the local legislation and institutional requirements. Written informed consent for participation was not required from the participants or the participants’ legal guardians/next of kin because Our data comes from reanalysis of public databases, so no additional ethical approval is required.

## Author contributions

ZJ: Funding acquisition, Methodology, Writing – original draft. XLY: Formal Analysis, Methodology, Software, Writing – review and editing. WL: Resources, Validation, Writing – review and editing. FT: Supervision, Visualization, Writing – review and editing. WM: Funding acquisition, Project administration, Writing – review and editing. XMY: Data curation, Formal Analysis, Supervision, Writing – review and editing. CC: Investigation, Project administration, Supervision, Validation, Writing – review and editing. XC: Conceptualization, Data curation, Formal Analysis, Methodology, Software, Visualization, Writing – review and editing.

## Glossary

**Table:**

β-NGF	Beta-nerve growth factor
CTACK	Cutaneous T-cell attracting
Eotaxin	Eotaxin
FGF-basic	Fibroblast growth factor basic
G-CSF	Granulocyte-colony stimulating factor
GRO-α	Growth-regulated protein alpha
HGF	Hepatocyte growth factor
INF-γ	Interferon gamma
IL-1rα	Interleukin-1-receptor antagonist
IL-1β	Interleukin-1-beta
IL-2	Interleukin-2
IL-2rα	Interleukin-2 receptor antagonist
IL-4	Interleukin-4
IL-5	Interleukin-5
IL-6	Interleukin-6
IL-7	Interleukin-7
IL-8	Interleukin-8
IL-9	Interleukin-9
IL-10	Interleukin-10
IL-12p70	Interleukin-12p70
IL-13	Interleukin-13
IL-16	Interleukin-16
IL-17	Interleukin-17
IL-18	Interleukin-18
IP-10	Interferon gamma-induced protein 10
MCP-1	Monocyte chemoattractant protein-1
MCP-3	Monocyte chemoattractant protein-3
M-CSF	Macrophage colony stimulating factor
MIF	Macrophage Migration Inhibitory Factor
MIG	Monokine induced by gamma interferon
MIP-1α	Macrophage inflammatory protein 1α
MIP-1β	Macrophage inflammatory protein 1β
PDGF-bb	Platelet-derived growth factor BB
RANTES	Regulated on activation, normal T Cell expressed and secreted
SCF	Stem cell factor
SCGF-β	Stem cell growth factor beta
SDF-1α	Stromal-cell-derived factor 1 alpha
TNF-α	Tumor necrosis factor alpha
TNF-β	Tumor necrosis factor beta
TRAIL	TNF-related apoptosis inducing ligand
VEGF	Vascular endothelial growth factor
ebi-a-GCST004420	CTACK
ebi-a-GCST004441	IL-18
ebi-a-GCST004421	β-NGF
ebi-a-GCST004442	IL-17
ebi-a-GCST004422	VEGF
ebi-a-GCST004443	IL-13
ebi-a-GCST004423	MIF
ebi-a-GCST004444	IL-10
ebi-a-GCST004424	TRAIL
ebi-a-GCST004445	IL-8
ebi-a-GCST004425	TNF-β
ebi-a-GCST00444	IL-6
ebi-a-GCST004426	TNF-α
ebi-a-GCST00444	IL-1rα
ebi-a-GCST004427	SDF-1α
ebi-a-GCST004448	IL-1β
ebi-a-GCST004428	SCGF-β
ebi-a-GCST004449	HGF
ebi-a-GCST004429	SCF
ebi-a-GCST004450	IL-9
ebi-a-GCST004430	IL-16
ebi-a-GCST00445	IL-7
ebi-a-GCST004431	RANTES
ebi-a-GCST004452	IL-5
ebi-a-GCST004432	PDGF-bb
ebi-a-GCST004453	IL-4
ebi-a-GCST004433	MIP-1β
ebi-a-GCST004454	IL-2
ebi-a-GCST004434	MIP-1α
ebi-a-GCST004455	IL-2rα
ebi-a-GCST004435	MIG
ebi-a-GCST004456	INF-γ
ebi-a-GCST004436	M-CSF
ebi-a-GCST004457	GRO-α
ebi-a-GCST004437	MCP-3
ebi-a-GCST004458	G-CSF
ebi-a-GCST004438	MCP-1
ebi-a-GCST004459	FGF-basic
ebi-a-GCST004439	IL-12p70
ebi-a-GCST004460	Eotaxin
ebi-a-GCST004440	IP-10
