# Supplementary material for: Associations of the circulating levels of cytokines with risk of systemic sclerosis: a bidirectional Mendelian randomized study
Source: Front Immunol. 2024 Feb 28;15:1330560. doi: 10.3389/fimmu.2024.1330560 (PMC10933062; doi:10.3389/fimmu.2024.1330560)
Supplement: Supplementary file 1 [file Table_1.doc]

| **Supplementary Table 1**. Basic information of research data | | | |
| --- | --- | --- | --- |
| Exposure/outcome | Sample size | Population | PMID/[GWAS ID](https://gwas.mrcieu.ac.uk/datasets/?sort=-gwas_id) |
| Circulating levels of cytokines | 8293 | European | 27989323 |
| Systemic sclerosis | 302 cases and  213,145 controls | European | finn-b-M13_SYSTSLCE |
| Drugs used in diabetes | 305,913 | European | 31015401 |
| Antithrombotic agents | 153,639 | European | 31015401 |
| Beta blocking agents | 224,024 | European | 31015401 |
| Calcium channel blockers | 204,378 | European | 31015401 |
| HMG CoA reductase inhibitors | 290,385 | European | 31015401 |
| Immunosuppressants | 272,602 | European | 31015401 |
| Anti-inflammatory and antirheumatic products, non-steroids | 164,520 | European | 31015401 |
| Drugs affecting bone structure and mineralization | 215,668 | European | 31015401 |
| Glucocorticoids | 205,700 | European | 31015401 |

| **Supplementary Table 2**. Details of the number of genetic instruments and *F*-statistic for each cytokine and growth factor.. | | | |
| --- | --- | --- | --- |
| Cytokines/Growth factors | Abbreviations | No.of SNPs | *F*-statistic (range) |
| Beta-nerve growth factor | β-NGF | 7 | 24.17（20.77-36.50） |
| Cutaneous T-cell attracting | CTACK | 8 | 38.93（21.43-142.66） |
| Eotaxin | Eotaxin | 15 | 42.24（20.81-203.26） |
| Fibroblast growth factor basic | FGF-basic | 4 | 22.83（20.97-24.43） |
| Granulocyte-colony stimulating factor | G-CSF | 8 | 23.30（21.16-25.15） |
| Growth-regulated protein alpha | GRO-α | 8 | 71.46（21.01-250.49） |
| Hepatocyte growth factor | HGF | 7 | 29.68（20.65-57.25） |
| Interferon gamma | INF-γ | 8 | 23.38（21.74-27.68） |
| Interleukin-1-receptor antagonist | IL-1rα | 5 | 22.73（21.30-24.05） |
| Interleukin-1-beta | IL-1β | 4 | 15.36（14.62-16.78） |
| Interleukin-2 | IL-2 | 6 | 46.35（21.28-167.61） |
| Interleukin-2 receptor antagonist | IL-2rα | 9 | 22.81（20.97-28.23） |
| Interleukin-4 | IL-4 | 9 | 23.24（21.08-26.19） |
| Interleukin-5 | IL-5 | 5 | 25.95（22.06-37.93） |
| Interleukin-6 | IL-6 | 5 | 24.94（21.82-29.31） |
| Interleukin-7 | IL-7 | 9 | 39.59（20.59-169.84） |
| Interleukin-8 | IL-8 | 4 | 23.95（22.10-25.85） |
| Interleukin-9 | IL-9 | 6 | 22.50（21.23-26.46） |
| Interleukin-10 | IL-10 | 10 | 52.49（21.29-298.98） |
| Interleukin-12p70 | IL-12p70 | 10 | 79.42（20.80-564.29） |
| Interleukin-13 | IL-13 | 9 | 52.88（21.10-292.85） |
| Interleukin-16 | IL-16 | 10 | 37.47（20.88-131.98） |
| Interleukin-17 | IL-17 | 9 | 24.15（20.41-38.97） |
| Interleukin-18 | IL-18 | 15 | 35.22（20.61-96.17） |
| Interferon gamma-induced protein 10 | IP-10 | 9 | 23.43（21.00-31.11） |
| Monocyte chemoattractant protein-1 | MCP-1 | 13 | 43.39（20.94-198.72） |
| Monocyte chemoattractant protein-3 | MCP-3 | 3 | 23.82（22.68-25.68） |
| Macrophage colony stimulating factor | M-CSF | 8 | 23.63（21.25-31.64） |
| Macrophage Migration Inhibitory Factor | MIF | 6 | 24.52（21.20-28.11） |
| Monokine induced by gamma interferon | MIG | 14 | 24.95（20.85-42.38） |
| Macrophage inflammatory protein 1α | MIP-1α | 6 | 22.40（21.95-22.81） |
| Macrophage inflammatory protein 1β | MIP-1β | 17 | 114.45（20.56-789.15） |
| Platelet-derived growth factor BB | PDGF-bb | 13 | 48.09（21.00-245.35） |
| Regulated on activation,normal T Cell expressed and secreted | RANTES | 9 | 24.10（20.87-29.99） |
| Stem cell factor | SCF | 9 | 27.98（20.80-48.67） |
| Stem cell growth factor beta | SCGF-β | 14 | 34.86（20.72-99.38） |
| Stromal-cell-derived factor 1 alpha | SDF-1α | 7 | 16.47（11.16-21.69） |
| Tumor necrosis factor alpha | TNF-α | 5 | 23.51（22.07-24.95） |
| Tumor necrosis factor beta | TNF-β | 4 | 36.92（21.90-79.96） |
| TNF-related apoptosis inducing ligand | TRAIL | 13 | 88.79（19.86-370.00） |
| Vascular endothelial growth factor | VEGF | 10 | 105.48（22.07-784.00） |

| **Supplementary Table 3.** Details of the number of SSC genetic instruments . | | | | | |
| --- | --- | --- | --- | --- | --- |
| SNP | SE | Beta | Effect_allele | Chr | *P*-value |
| rs2222631 | 0.083 | 0.397 | 0.455 | 3 | 1.78×10^-6^ |
| rs141520926 | 0.309 | 1.435 | 0.024 | 6 | 3.34×10^-6^ |
| rs72840563 | 0.197 | 0.989 | 0.054 | 6 | 4.88×10^-7^ |
| rs36030018 | 0.154 | 0.924 | 0.095 | 6 | 1.76×10^-9^ |
| rs3807307 | 0.083 | 0.396 | 0.428 | 7 | 1.96×10^-6^ |
| rs2501203 | 0.108 | -0.493 | 0.813 | 13 | 4.63×10^-6^ |
| rs117642611 | 1.300 | 6.016 | 0.002 | 18 | 3.71×10^-6^ |

| **Supplementary Table 4** Heterogeneity test | | | | |
| --- | --- | --- | --- | --- |
| Exposure | Method | Q | Q_df | Q_pval |
| β-NGF | MR Egger | 2.239 | 5 | 0.815 |
|  | Inverse variance weighted | 3.428 | 6 | 0.753 |
| CTACK | MR Egger | 4.112 | 6 | 0.662 |
|  | Inverse variance weighted | 6.036 | 7 | 0.536 |
| Eotaxin | MR Egger | 7.232 | 13 | 0.890 |
|  | Inverse variance weighted | 8.208 | 14 | 0.878 |
| FGF-bacic | MR Egger | 0.682 | 2 | 0.711 |
|  | Inverse variance weighted | 3.800 | 3 | 0.284 |
| G-CSF | MR Egger | 1.979 | 6 | 0.922 |
|  | Inverse variance weighted | 2.946 | 7 | 0.890 |
| GRO-α | MR Egger | 6.232 | 6 | 0.398 |
|  | Inverse variance weighted | 6.477 | 7 | 0.485 |
| HGF | MR Egger | 10.709 | 5 | 0.057 |
|  | Inverse variance weighted | 12.804 | 6 | 0.046 |
| INF-γ | MR Egger | 7.245 | 6 | 0.299 |
|  | Inverse variance weighted | 8.613 | 7 | 0.282 |
| IL-1rα | MR Egger | 2.492 | 3 | 0.477 |
|  | Inverse variance weighted | 5.205 | 4 | 0.267 |
| IL-1β | MR Egger | 3.409 | 2 | 0.182 |
|  | Inverse variance weighted | 3.979 | 3 | 0.264 |
| IL-2 | MR Egger | 6.615 | 4 | 0.158 |
|  | Inverse variance weighted | 7.118 | 5 | 0.212 |
| IL-2rα | MR Egger | 9.208 | 7 | 0.238 |
|  | Inverse variance weighted | 9.519 | 8 | 0.300 |
| IL-4 | MR Egger | 3.178 | 7 | 0.868 |
|  | Inverse variance weighted | 5.432 | 8 | 0.711 |
| IL-5 | MR Egger | 1.690 | 3 | 0.639 |
|  | Inverse variance weighted | 2.250 | 4 | 0.690 |
| IL-6 | MR Egger | 2.660 | 3 | 0.447 |
|  | Inverse variance weighted | 2.767 | 4 | 0.598 |
| IL-7 | MR Egger | 13.403 | 7 | 0.063 |
|  | Inverse variance weighted | 13.645 | 8 | 0.091 |
| IL-8 | MR Egger | 3.727 | 2 | 0.155 |
|  | Inverse variance weighted | 3.868 | 3 | 0.276 |
| IL-9 | MR Egger | 5.812 | 4 | 0.214 |
|  | Inverse variance weighted | 5.921 | 5 | 0.314 |
| IL-10 | MR Egger | 11.311 | 8 | 0.185 |
|  | Inverse variance weighted | 11.869 | 9 | 0.221 |
| IL-12p70 | MR Egger | 4.800 | 8 | 0.779 |
|  | Inverse variance weighted | 4.800 | 9 | 0.851 |
| IL-13 | MR Egger | 2.879 | 7 | 0.896 |
|  | Inverse variance weighted | 4.558 | 8 | 0.804 |
| IL-16 | MR Egger | 12.385 | 8 | 0.135 |
|  | Inverse variance weighted | 12.411 | 9 | 0.191 |
| IL-17 | MR Egger | 9.316 | 7 | 0.231 |
|  | Inverse variance weighted | 10.905 | 8 | 0.207 |
| IL-18 | MR Egger | 12.429 | 13 | 0.493 |
|  | Inverse variance weighted | 15.295 | 14 | 0.358 |
| IP-10 | MR Egger | 13.571 | 7 | 0.059 |
|  | Inverse variance weighted | 13.609 | 8 | 0.093 |
| MCP-1 | MR Egger | 16.896 | 11 | 0.111 |
|  | Inverse variance weighted | 18.877 | 12 | 0.092 |
| MCP-3 | MR Egger | 0.511 | 1 | 0.475 |
|  | Inverse variance weighted | 1.937 | 2 | 0.380 |
| M-CSF | MR Egger | 4.506 | 6 | 0.608 |
|  | Inverse variance weighted | 5.093 | 7 | 0.649 |
| MIF | MR Egger | 5.482 | 4 | 0.241 |
|  | Inverse variance weighted | 6.091 | 5 | 0.297 |
| MIG | MR Egger | 10.595 | 12 | 0.564 |
|  | Inverse variance weighted | 10.682 | 13 | 0.637 |
| MIP-1α | MR Egger | 3.164 | 4 | 0.531 |
|  | Inverse variance weighted | 3.186 | 5 | 0.671 |
| MIP-1β | MR Egger | 12.383 | 15 | 0.650 |
|  | Inverse variance weighted | 17.534 | 16 | 0.352 |
| PDGF-bb | MR Egger | 9.753 | 11 | 0.553 |
|  | Inverse variance weighted | 9.768 | 12 | 0.636 |
| RANTES | MR Egger | 6.326 | 7 | 0.502 |
|  | Inverse variance weighted | 6.662 | 8 | 0.573 |
| SCF | MR Egger | 20.668 | 7 | 0.004 |
|  | Inverse variance weighted | 20.747 | 8 | 0.008 |
| SCGF-β | MR Egger | 6.672 | 12 | 0.878 |
|  | Inverse variance weighted | 12.120 | 13 | 0.518 |
| SDF-1α | MR Egger | 5.408 | 5 | 0.368 |
|  | Inverse variance weighted | 5.680 | 6 | 0.460 |
| TNF-α | MR Egger | 7.180 | 3 | 0.066 |
|  | Inverse variance weighted | 7.182 | 4 | 0.127 |
| TNF-β | MR Egger | 1.014 | 2 | 0.602 |
|  | Inverse variance weighted | 3.792 | 3 | 0.285 |
| TRAIL | MR Egger | 10.742 | 11 | 0.465 |
|  | Inverse variance weighted | 11.295 | 12 | 0.504 |
| VEGF | MR Egger | 6.785 | 8 | 0.560 |
|  | Inverse variance weighted | 6.813 | 9 | 0.657 |

| **Supplementary Table5** Horizontal gene pleiotropy test | | | |
| --- | --- | --- | --- |
| Exposure | Egger_intercept | SE | *P*-value |
| β-NGF | -0.202 | 0.185 | 0.325 |
| CTACK | 0.130 | 0.094 | 0.215 |
| Eotaxin | -0.068 | 0.069 | 0.341 |
| FGF-bacic | 0.441 | 0.250 | 0.219 |
| G-CSF | -0.075 | 0.076 | 0.363 |
| GRO-α | -0.050 | 0.103 | 0.645 |
| HGF | 0.179 | 0.181 | 0.368 |
| INF-γ | 0.105 | 0.098 | 0.328 |
| IL-1rα | -0.222 | 0.135 | 0.198 |
| IL-1β | -0.103 | 0.178 | 0.622 |
| IL-2 | 0.066 | 0.120 | 0.611 |
| IL-2rα | -0.040 | 0.082 | 0.641 |
| IL-4 | 0.156 | 0.104 | 0.177 |
| IL-5 | -0.093 | 0.124 | 0.509 |
| IL-6 | -0.040 | 0.123 | 0.765 |
| IL-7 | -0.058 | 0.163 | 0.733 |
| IL-8 | 0.049 | 0.179 | 0.809 |
| IL-9 | -0.052 | 0.190 | 0.797 |
| IL-10 | 0.045 | 0.072 | 0.547 |
| IL-12p70 | 0.000 | 0.056 | 0.999 |
| IL-13 | -0.100 | 0.077 | 0.236 |
| IL-16 | 0.012 | 0.094 | 0.900 |
| IL-17 | -0.116 | 0.106 | 0.311 |
| IL-18 | -0.119 | 0.070 | 0.114 |
| IP-10 | 0.016 | 0.114 | 0.893 |
| MCP-1 | -0.101 | 0.089 | 0.280 |
| MCP-3 | -0.258 | 0.216 | 0.444 |
| M-CSF | -0.081 | 0.106 | 0.473 |
| MIF | 0.094 | 0.141 | 0.542 |
| MIG | 0.027 | 0.091 | 0.773 |
| MIP-1α | 0.020 | 0.135 | 0.891 |
| MIP-1β | -0.118 | 0.052 | 0.038 |
| PDGF-bb | 0.007 | 0.059 | 0.903 |
| RANTES | 0.074 | 0.127 | 0.580 |
| SCF | 0.026 | 0.161 | 0.875 |
| SCGF-β | 0.144 | 0.062 | 0.038 |
| SDF-1α | 0.046 | 0.092 | 0.637 |
| TNF-α | -0.004 | 0.159 | 0.981 |
| TNF-β | -0.151 | 0.091 | 0.237 |
| TRAIL | -0.041 | 0.055 | 0.473 |
| VEGF | 0.010 | 0.058 | 0.870 |

| **Supplementary Table 6** MR analysis of SSC as an exposure factor | | | |
| --- | --- | --- | --- |
| Methods | No. of SNPs | OR (95 CI) | *P*-value |
| **β-NGF** |  |  |  |
| Inverse variance weighted | 4 | 1.031(0.972-1.093) | 0.309 |
| MR Egger | 4 | 1.009(0.862-1.182) | 0.920 |
| Weighted median | 4 | 1.022(0.955-1.093) | 0.531 |
| Weighted mode | 4 | 1.021(0.934-1.116) | 0.678 |
| **CTACK** |  |  |  |
| Inverse variance weighted | 4 | 0.995(0.939-1.053) | 0.850 |
| MR Egger | 4 | 1.052(0.902-1.226) | 0.585 |
| Weighted median | 4 | 0.996(0.932-1.063) | 0.899 |
| Weighted mode | 4 | 1.010(0.916-1.114) | 0.853 |
| **Eotaxin** |  |  |  |
| Inverse variance weighted | 4 | 0.981(0.939-1.024) | 0.385 |
| MR Egger | 4 | 0.955(0.834-1.093) | 0.573 |
| Weighted median | 4 | 0.981(0.936-1.028) | 0.417 |
| Weighted mode | 4 | 0.955(0.887-1.028) | 0.309 |
| **FGF-bacic** |  |  |  |
| Inverse variance weighted | 4 | 0.988(0.945-1.033) | 0.592 |
| MR Egger | 4 | 1.013(0.878-1.168) | 0.878 |
| Weighted median | 4 | 0.987(0.939-1.037) | 0.598 |
| Weighted mode | 4 | 0.986(0.920-1.057) | 0.724 |
| **G-CSF** |  |  |  |
| Inverse variance weighted | 4 | 0.962(0.899-1.030) | 0.269 |
| MR Egger | 4 | 0.953(0.765-1.187) | 0.709 |
| Weighted median | 4 | 0.982(0.931-1.036) | 0.502 |
| Weighted mode | 4 | 1.001(0.925-1.083) | 0.983 |
| **GRO-α** |  |  |  |
| Inverse variance weighted | 4 | 0.989(0.931-1.050) | 0.714 |
| MR Egger | 4 | 0.989(0.809-1.208) | 0.921 |
| Weighted median | 4 | 0.986(0.919-1.057) | 0.685 |
| Weighted mode | 4 | 0.976(0.884-1.078) | 0.668 |
| **HGF** |  |  |  |
| Inverse variance weighted | 4 | 0.999(0.962-1.037) | 0.948 |
| MR Egger | 4 | 0.986(0.892-1.089) | 0.803 |
| Weighted median | 4 | 0.990(0.948-1.035) | 0.662 |
| Weighted mode | 4 | 0.984(0.924-1.047) | 0.644 |
| **INF-γ** |  |  |  |
| Inverse variance weighted | 4 | 0.974(0.930-1.020) | 0.260 |
| MR Egger | 4 | 0.944(0.820-1.086) | 0.503 |
| Weighted median | 4 | 0.978(0.932-1.027 | 0.374 |
| Weighted mode | 4 | 1.003(0.923-1.090 | 0.940 |
| **IL-1rα** |  |  |  |
| Inverse variance weighted | 4 | 0.991(0.936-1.049 ) | 0.765 |
| MR Egger | 4 | 1.004(0.863-1.169) | 0.959 |
| Weighted median | 4 | 1.001(0.936-1.071) | 0.970 |
| Weighted mode | 4 | 1.009(0.921-1.106) | 0.854 |
| **IL-1β** |  |  |  |
| Inverse variance weighted | 4 | 0.991(0.928-1.059) | 0.795 |
| MR Egger | 4 | 1.049(0.859-1.281) | 0.684 |
| Weighted median | 4 | 1.011(0.955-1.071) | 0.704 |
| Weighted mode | 4 | 1.036(0.954-1.124) | 0.466 |
| **IL-2** |  |  |  |
| Inverse variance weighted | 4 | 1.002(0.947-1.061) | 0.941 |
| MR Egger | 4 | 1.000(0.860-1.163) | 1.000 |
| Weighted median | 4 | 1.007(0.943-1.075) | 0.837 |
| Weighted mode | 4 | 1.019(0.928-1.120) | 0.717 |
| **IL-2rα** |  |  |  |
| Inverse variance weighted | 4 | 0.983(0.928-1.042) | 0.573 |
| MR Egger | 4 | 0.938(0.802-1.098) | 0.509 |
| Weighted median | 4 | 0.985(0.922-1.053) | 0.668 |
| Weighted mode | 4 | 0.987(0.899-1.083) | 0.797 |
| **IL-4** |  |  |  |
| Inverse variance weighted | 4 | 0.985(0.947-1.023) | 0.429 |
| MR Egger | 4 | 0.965(0.866-1.075) | 0.582 |
| Weighted median | 4 | 0.991(0.946-1.038) | 0.710 |
| Weighted mode | 4 | 1.009(0.940-1.084) | 0.813 |
| **IL-5** |  |  |  |
| Inverse variance weighted | 4 | 0.995(0.937-1.055) | 0.858 |
| MR Egger | 4 | 0.970(0.826-1.138) | 0.742 |
| Weighted median | 4 | 0.984(0.919-1.052) | 0.629 |
| Weighted mode | 4 | 0.982(0.899-1.072) | 0.712 |
| **IL-6** |  |  |  |
| Inverse variance weighted | 4 | 0.996(0.956-1.038) | 0.857 |
| MR Egger | 4 | 0.949(0.848-1.062) | 0.459 |
| Weighted median | 4 | 0.984(0.940-1.030) | 0.485 |
| Weighted mode | 4 | 0.969(0.904-1.039) | 0.443 |
| **IL-7** |  |  |  |
| Inverse variance weighted | 4 | 0.961(0.906-1.019) | 0.181 |
| MR Egger | 4 | 0.976(0.833-1.143) | 0.791 |
| Weighted median | 4 | 0.959(0.897-1.025) | 0.221 |
| Weighted mode | 4 | 0.940(0.857-1.032) | 0.287 |
| **IL-8** |  |  |  |
| Inverse variance weighted | 4 | 1.002(0.946-1.062) | 0.935 |
| MR Egger | 4 | 1.015(0.867-1.188) | 0.870 |
| Weighted median | 4 | 1.008(0.944-1.076) | 0.816 |
| Weighted mode | 4 | 1.009(0.930-1.094) | 0.843 |
| **IL-9** |  |  |  |
| Inverse variance weighted | 4 | 1.006(0.948-1.066) | 0.853 |
| MR Egger | 4 | 1.000(0.827-1.210) | 0.998 |
| Weighted median | 4 | 0.985(0.917-1.059) | 0.691 |
| Weighted mode | 4 | 0.981(0.890-1.082) | 0.728 |
| **IL-10** |  |  |  |
| Inverse variance weighted | 4 | 0.975(0.910-1.044) | 0.464 |
| MR Egger | 4 | 0.956(0.766-1.194) | 0.730 |
| Weighted median | 4 | 0.989(0.937-1.044) | 0.695 |
| Weighted mode | 4 | 1.008(0.927-1.095) | 0.871 |
| **IL-12p70** |  |  |  |
| Inverse variance weighted | 4 | 0.990(0.924-1.061) | 0.776 |
| MR Egger | 4 | 0.925(0.757-1.130) | 0.526 |
| Weighted median | 4 | 0.980(0.935-1.027) | 0.391 |
| Weighted mode | 4 | 0.951(0.869-1.041) | 0.353 |
| **IL-13** |  |  |  |
| Inverse variance weighted | 4 | 0.995(0.933-1.061) | 0.880 |
| MR Egger | 4 | 0.886(0.757-1.037) | 0.270 |
| Weighted median | 4 | 0.998(0.929-1.073) | 0.959 |
| Weighted mode | 4 | 1.004(0.907-1.112) | 0.940 |
| **IL-16** |  |  |  |
| Inverse variance weighted | 4 | 1.002(0.920-1.091) | 0.966 |
| MR Egger | 4 | 0.959(0.726-1.265) | 0.794 |
| Weighted median | 4 | 0.992(0.921-1.070) | 0.843 |
| Weighted mode | 4 | 0.955(0.836-1.091) | 0.546 |
| **IL-17** |  |  |  |
| Inverse variance weighted | 4 | 0.975(0.935-1.017) | 0.239 |
| MR Egger | 4 | 0.948(0.831-1.080) | 0.506 |
| Weighted median | 4 | 0.961(0.916-1.009) | 0.114 |
| Weighted mode | 4 | 0.958(0.895-1.025) | 0.301 |
| **IL-18** |  |  |  |
| Inverse variance weighted | 4 | 1.027(0.959-1.100) | 0.453 |
| MR Egger | 4 | 1.070(0.861-1.331) | 0.603 |
| Weighted median | 4 | 1.053(0.979-1.132) | 0.165 |
| Weighted mode | 4 | 1.065(0.961-1.181) | 0.315 |
| **IP-10** |  |  |  |
| Inverse variance weighted | 4 | 1.039(0.935-1.155) | 0.478 |
| MR Egger | 4 | 0.993(0.707-1.396) | 0.973 |
| Weighted median | 4 | 1.008(0.933-1.090) | 0.831 |
| Weighted mode | 4 | 1.001(0.918-1.091) | 0.989 |
| **MCP-1** |  |  |  |
| Inverse variance weighted | 4 | 1.010(0.972-1.049) | 0.620 |
| MR Egger | 4 | 0.975(0.882-1.078) | 0.671 |
| Weighted median | 4 | 1.014(0.972-1.059) | 0.511 |
| Weighted mode | 4 | 1.016(0.958-1.077) | 0.628 |
| **MCP-3** |  |  |  |
| Inverse variance weighted | 3 | 1.008(0.893-1.138) | 0.898 |
| MR Egger | 3 | 0.880(0.165-4.699) | 0.906 |
| Weighted median | 3 | 0.985(0.843-1.152) | 0.853 |
| Weighted mode | 3 | 0.964(0.799-1.163) | 0.738 |
| **M-CSF** |  |  |  |
| Inverse variance weighted | 4 | 0.976(0.877-1.086) | 0.658 |
| MR Egger | 4 | 1.134(0.867-1.482) | 0.455 |
| Weighted median | 4 | 0.976(0.887-1.074) | 0.618 |
| Weighted mode | 4 | 0.980(0.846-1.136) | 0.809 |
| **MIF** |  |  |  |
| Inverse variance weighted | 4 | 1.011(0.939-1.089) | 0.772 |
| MR Egger | 4 | 0.929(0.753-1.145) | 0.560 |
| Weighted median | 4 | 1.000(0.928-1.078) | 0.994 |
| Weighted mode | 4 | 0.991(0.889-1.105) | 0.877 |
| **MIG** |  |  |  |
| Inverse variance weighted | 4 | 1.030(0.974-1.090) | 0.302 |
| MR Egger | 4 | 0.977(0.840-1.137) | 0.794 |
| Weighted median | 4 | 1.019(0.952-1.091) | 0.584 |
| Weighted mode | 4 | 1.001(0.910-1.102) | 0.978 |
| **MIP-1α** |  |  |  |
| Inverse variance weighted | 4 | 0.982(0.926-1.041) | 0.536 |
| MR Egger | 4 | 0.932(0.796-1.092) | 0.476 |
| Weighted median | 4 | 0.987(0.925-1.053) | 0.697 |
| Weighted mode | 4 | 0.995(0.911-1.086) | 0.911 |
| **MIP-1β** |  |  |  |
| Inverse variance weighted | 4 | 0.987(0.950-1.025) | 0.490 |
| MR Egger | 4 | 0.935(0.846-1.034) | 0.319 |
| Weighted median | 4 | 0.991(0.947-1.038) | 0.714 |
| Weighted mode | 4 | 1.013(0.943-1.089) | 0.745 |
| **PDGF-bb** |  |  |  |
| Inverse variance weighted | 4 | 0.995(0.958-1.034) | 0.813 |
| MR Egger | 4 | 0.982(0.879-1.098) | 0.784 |
| Weighted median | 4 | 0.979(0.937-1.024) | 0.354 |
| Weighted mode | 4 | 0.976(0.918-1.038) | 0.496 |
| **RANTES** |  |  |  |
| Inverse variance weighted | 4 | 1.023(0.965-1.085) | 0.444 |
| MR Egger | 4 | 1.117(0.955-1.305) | 0.300 |
| Weighted median | 4 | 1.012(0.945-1.085) | 0.729 |
| Weighted mode | 4 | 1.007(0.910-1.115) | 0.896 |
| **SCF** |  |  |  |
| Inverse variance weighted | 4 | 1.032(0.993-1.071) | 0.107 |
| MR Egger | 4 | 1.080(0.977-.194) | 0.269 |
| Weighted median | 4 | 1.032(0.986-1.080) | 0.170 |
| Weighted mode | 4 | 1.036(0.975-1.101) | 0.333 |
| **SCGF-β** |  |  |  |
| Inverse variance weighted | 4 | 0.998(0.944-1.057) | 0.957 |
| MR Egger | 4 | 0.963(0.828-1.121) | 0.676 |
| Weighted median | 4 | 0.983(0.917-1.053) | 0.618 |
| Weighted mode | 4 | 0.970(0.883-1.066) | 0.572 |
| **SDF-1α** |  |  |  |
| Inverse variance weighted | 4 | 0.979(0.933-1.027) | 0.386 |
| MR Egger | 4 | 0.941(0.815-1.087) | 0.497 |
| Weighted median | 4 | 0.982(0.935-1.032) | 0.482 |
| Weighted mode | 4 | 1.014(0.931-1.104) | 0.769 |
| **TNF-α** |  |  |  |
| Inverse variance weighted | 4 | 1.010(0.952-1.071) | 0.746 |
| MR Egger | 4 | 1.009(0.860-1.184) | 0.923 |
| Weighted median | 4 | 1.016(0.947-1.091) | 0.652 |
| Weighted mode | 4 | 1.027(0.938-1.125) | 0.602 |
| **TNF-β** |  |  |  |
| Inverse variance weighted | 3 | 1.008(0.910-1.117) | 0.871 |
| MR Egger | 3 | 0.673(0.239-1.896) | 0.591 |
| Weighted median | 3 | 1.026(0.904-1.165) | 0.689 |
| Weighted mode | 3 | 1.038(0.897-1.201) | 0.668 |
| **TRAIL** |  |  |  |
| Inverse variance weighted | 4 | 1.024(0.986-1.064) | 0.224 |
| MR Egger | 4 | 1.018(0.921-1.126) | 0.759 |
| Weighted median | 4 | 1.031(0.986-1.077) | 0.176 |
| Weighted mode | 4 | 1.035(0.977-1.097) | 0.329 |
| **VEGF** |  |  |  |
| Inverse variance weighted | 4 | 0.991(0.950-1.032) | 0.654 |
| MR Egger | 4 | 0.984(0.879-1.101) | 0.805 |
| Weighted median | 4 | 0.999(0.949-1.052) | 0.969 |
| Weighted mode | 4 | 1.014(0.944-1.089) | 0.726 |

| **Supplementary Table 7** Heterogeneity test | | | | |
| --- | --- | --- | --- | --- |
| Exposure | Method | Q | Q_df | Q_pval |
| β-NGF | MR Egger | 0.369 | 2 | 0.832 |
|  | Inverse variance weighted | 0.448 | 3 | 0.930 |
| CTACK | MR Egger | 1.502 | 2 | 0.472 |
|  | Inverse variance weighted | 2.097 | 3 | 0.553 |
| Eotaxin | MR Egger | 3.543 | 2 | 0.170 |
|  | Inverse variance weighted | 3.854 | 3 | 0.278 |
| FGF-bacic | MR Egger | 3.525 | 2 | 0.172 |
|  | Inverse variance weighted | 3.765 | 3 | 0.288 |
| G-CSF | MR Egger | 9.159 | 2 | 0.010 |
|  | Inverse variance weighted | 9.201 | 3 | 0.027 |
| GRO-α | MR Egger | 3.246 | 2 | 0.197 |
|  | Inverse variance weighted | 3.246 | 3 | 0.355 |
| HGF | MR Egger | 1.088 | 2 | 0.580 |
|  | Inverse variance weighted | 1.167 | 3 | 0.761 |
| INF-γ | MR Egger | 3.625 | 2 | 0.163 |
|  | Inverse variance weighted | 4.041 | 3 | 0.257 |
| IL-1rα | MR Egger | 1.105 | 2 | 0.575 |
|  | Inverse variance weighted | 1.139 | 3 | 0.768 |
| IL-1β | MR Egger | 5.37 | 2 | 0.068 |
|  | Inverse variance weighted | 6.332 | 3 | 0.097 |
| IL-2 | MR Egger | 1.17 | 2 | 0.557 |
|  | Inverse variance weighted | 1.171 | 3 | 0.760 |
| IL-2rα | MR Egger | 0.086 | 2 | 0.958 |
|  | Inverse variance weighted | 0.485 | 3 | 0.922 |
| IL-4 | MR Egger | 2.229 | 2 | 0.328 |
|  | Inverse variance weighted | 2.405 | 3 | 0.493 |
| IL-5 | MR Egger | 0.374 | 2 | 0.829 |
|  | Inverse variance weighted | 0.487 | 3 | 0.922 |
| IL-6 | MR Egger | 2.449 | 2 | 0.294 |
|  | Inverse variance weighted | 3.463 | 3 | 0.326 |
| IL-7 | MR Egger | 0.84 | 2 | 0.657 |
|  | Inverse variance weighted | 0.883 | 3 | 0.829 |
| IL-8 | MR Egger | 0.214 | 2 | 0.899 |
|  | Inverse variance weighted | 0.242 | 3 | 0.971 |
| IL-9 | MR Egger | 3.157 | 2 | 0.206 |
|  | Inverse variance weighted | 3.163 | 3 | 0.367 |
| IL-10 | MR Egger | 8.951 | 2 | 0.011 |
|  | Inverse variance weighted | 9.101 | 3 | 0.028 |
| IL-12p70 | MR Egger | 7.938 | 2 | 0.019 |
|  | Inverse variance weighted | 9.976 | 3 | 0.019 |
| IL-13 | MR Egger | 1.294 | 2 | 0.524 |
|  | Inverse variance weighted | 3.718 | 3 | 0.294 |
| IL-16 | MR Egger | 6.107 | 2 | 0.047 |
|  | Inverse variance weighted | 6.448 | 3 | 0.092 |
| IL-17 | MR Egger | 3.106 | 2 | 0.212 |
|  | Inverse variance weighted | 3.433 | 3 | 0.330 |
| IL-18 | MR Egger | 3.989 | 2 | 0.136 |
|  | Inverse variance weighted | 4.314 | 3 | 0.229 |
| IP-10 | MR Egger | 10.063 | 2 | 0.007 |
|  | Inverse variance weighted | 10.453 | 3 | 0.015 |
| MCP-1 | MR Egger | 0.299 | 2 | 0.861 |
|  | Inverse variance weighted | 0.841 | 3 | 0.840 |
| MCP-3 | MR Egger | 1.822 | 1 | 0.177 |
|  | Inverse variance weighted | 1.869 | 2 | 0.393 |
| M-CSF | MR Egger | 4.259 | 2 | 0.119 |
|  | Inverse variance weighted | 7.235 | 3 | 0.065 |
| MIF | MR Egger | 3.602 | 2 | 0.165 |
|  | Inverse variance weighted | 4.93 | 3 | 0.177 |
| MIG | MR Egger | 1.147 | 2 | 0.563 |
|  | Inverse variance weighted | 1.689 | 3 | 0.639 |
| MIP-1α | MR Egger | 0.346 | 2 | 0.841 |
|  | Inverse variance weighted | 0.824 | 3 | 0.844 |
| MIP-1β | MR Egger | 1.639 | 2 | 0.441 |
|  | Inverse variance weighted | 2.932 | 3 | 0.402 |
| PDGF-bb | MR Egger | 2.45 | 2 | 0.294 |
|  | Inverse variance weighted | 2.527 | 3 | 0.470 |
| RANTES | MR Egger | 0.098 | 2 | 0.952 |
|  | Inverse variance weighted | 1.505 | 3 | 0.681 |
| SCF | MR Egger | 0.311 | 2 | 0.856 |
|  | Inverse variance weighted | 1.261 | 3 | 0.738 |
| SCGF-β | MR Egger | 1.976 | 2 | 0.372 |
|  | Inverse variance weighted | 2.226 | 3 | 0.527 |
| SDF-1α | MR Egger | 3.834 | 2 | 0.147 |
|  | Inverse variance weighted | 4.473 | 3 | 0.215 |
| TNF-α | MR Egger | 0.591 | 2 | 0.744 |
|  | Inverse variance weighted | 0.591 | 3 | 0.899 |
| TNF-β | MR Egger | 0.029 | 1 | 0.865 |
|  | Inverse variance weighted | 0.619 | 2 | 0.734 |
| TRAIL | MR Egger | 0.742 | 2 | 0.690 |
|  | Inverse variance weighted | 0.755 | 3 | 0.860 |
| VEGF | MR Egger | 2.057 | 2 | 0.357 |
|  | Inverse variance weighted | 2.074 | 3 | 0.557 |

| **Supplementary Table 8** Horizontal gene pleiotropy test | | | |
| --- | --- | --- | --- |
| Outcome | Egger_intercept | SE | *P*-value |
| β-NGF | 0.011 | 0.039 | 0.804 |
| CTACK | -0.029 | 0.038 | 0.521 |
| Eotaxin | 0.014 | 0.034 | 0.716 |
| FGF-bacic | -0.013 | 0.035 | 0.748 |
| G-CSF | 0.005 | 0.055 | 0.932 |
| GRO-α | 0.000 | 0.050 | 0.999 |
| HGF | 0.007 | 0.025 | 0.806 |
| INF-γ | 0.017 | 0.035 | 0.679 |
| IL-1rα | -0.007 | 0.038 | 0.871 |
| IL-1β | -0.030 | 0.050 | 0.610 |
| IL-2 | 0.001 | 0.038 | 0.979 |
| IL-2rα | 0.025 | 0.039 | 0.592 |
| IL-4 | 0.011 | 0.027 | 0.730 |
| IL-5 | 0.013 | 0.040 | 0.769 |
| IL-6 | 0.026 | 0.028 | 0.459 |
| IL-7 | -0.008 | 0.039 | 0.854 |
| IL-8 | -0.007 | 0.039 | 0.883 |
| IL-9 | 0.003 | 0.048 | 0.959 |
| IL-10 | 0.010 | 0.056 | 0.871 |
| IL-12p70 | 0.036 | 0.050 | 0.548 |
| IL-13 | 0.061 | 0.039 | 0.260 |
| IL-16 | 0.023 | 0.069 | 0.770 |
| IL-17 | 0.015 | 0.033 | 0.691 |
| IL-18 | -0.022 | 0.054 | 0.726 |
| IP-10 | 0.024 | 0.085 | 0.807 |
| MCP-1 | 0.018 | 0.025 | 0.538 |
| MCP-3 | 0.057 | 0.360 | 0.899 |
| M-CSF | -0.079 | 0.067 | 0.359 |
| MIF | 0.045 | 0.052 | 0.481 |
| MIG | 0.028 | 0.038 | 0.538 |
| MIP-1α | 0.027 | 0.039 | 0.561 |
| MIP-1β | 0.029 | 0.025 | 0.373 |
| PDGF-bb | 0.007 | 0.028 | 0.825 |
| RANTES | -0.046 | 0.039 | 0.357 |
| SCF | -0.024 | 0.025 | 0.433 |
| SCGF-β | 0.019 | 0.038 | 0.666 |
| SDF-1α | 0.021 | 0.036 | 0.622 |
| TNF-α | 0.000 | 0.040 | 0.993 |
| TNF-β | 0.172 | 0.223 | 0.583 |
| TRAIL | 0.003 | 0.025 | 0.917 |
| VEGF | 0.004 | 0.028 | 0.912 |
