# Supplementary material for: Associations of the circulating levels of cytokines with risk of systemic sclerosis: a bidirectional Mendelian randomized study
Source: Front Immunol. 2024 Feb 28;15:1330560. doi: 10.3389/fimmu.2024.1330560 (PMC10933062; doi:10.3389/fimmu.2024.1330560)
Supplement: Supplementary file 2 [file DataSheet_1.pdf]

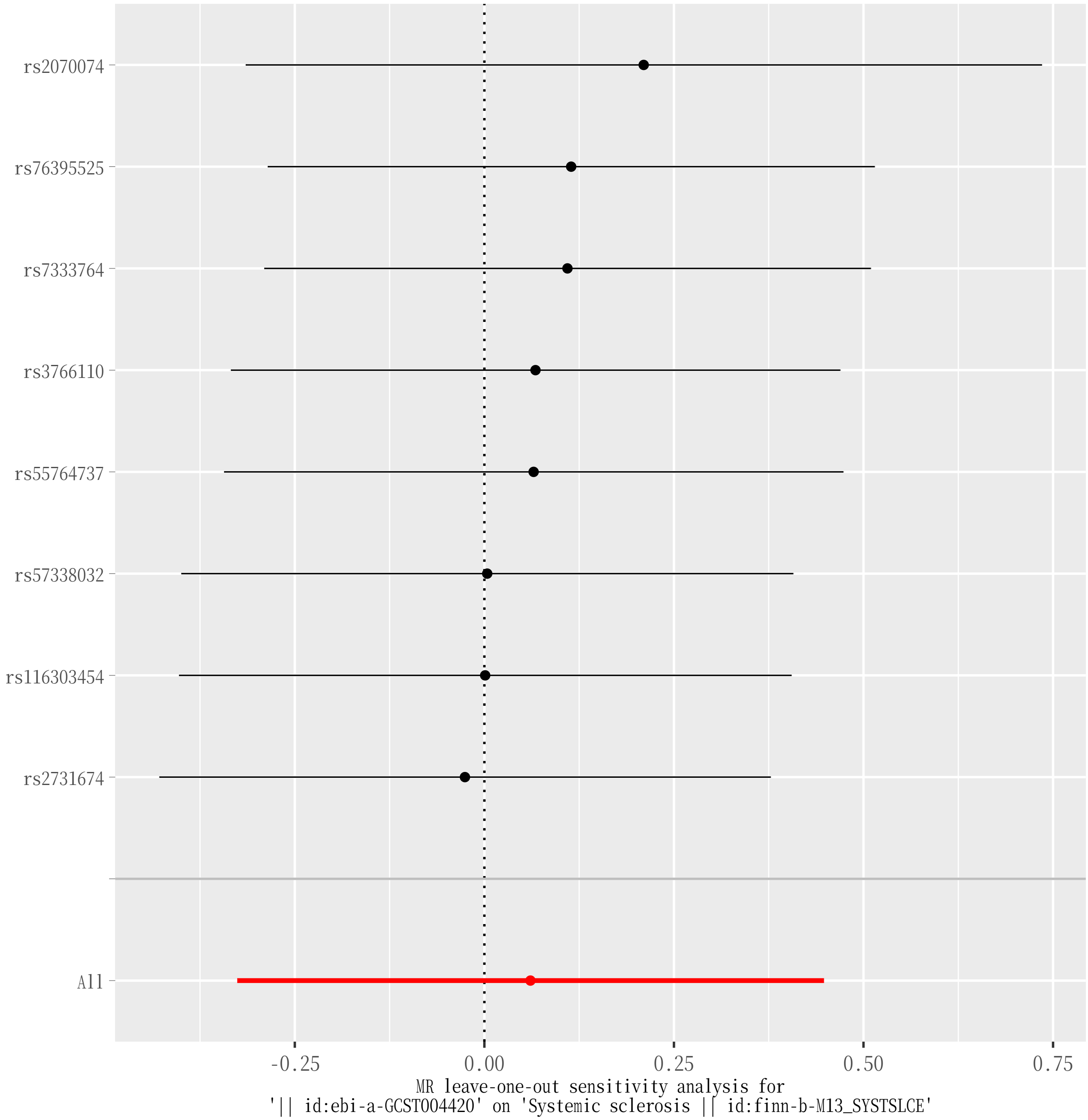
rs2070074
rs76395525
rs7333764
rs3766110
rs55764737
rs57338032
rs116303454
rs2731674
All
-0.25
0.00
0.25
0.50
0.75
MR leave-one-out sensitivity analysis for
'|| id:ebi-a-GCST004420' on 'Systemic sclerosis || id:finn-b-M13_SYSTSLCE'

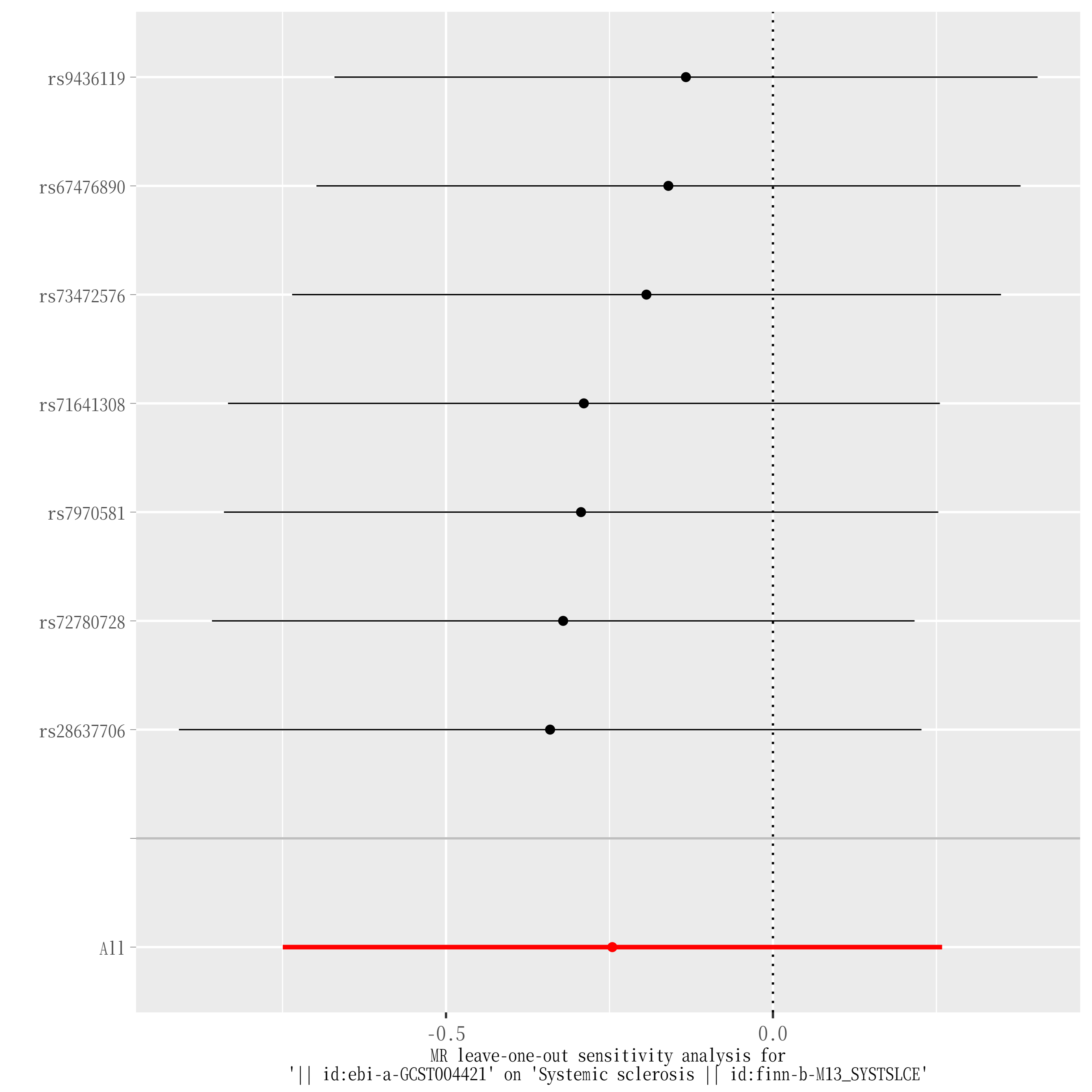
rs9436119
rs67476890
rs73472576
rs71641308
rs7970581
rs72780728
rs28637706
All
-0.5
0.0
MR leave-one-out sensitivity analysis for
'|| id:ebi-a-GCST004421' on 'Systemic sclerosis || id:finn-b-M13_SYSTSLCE'

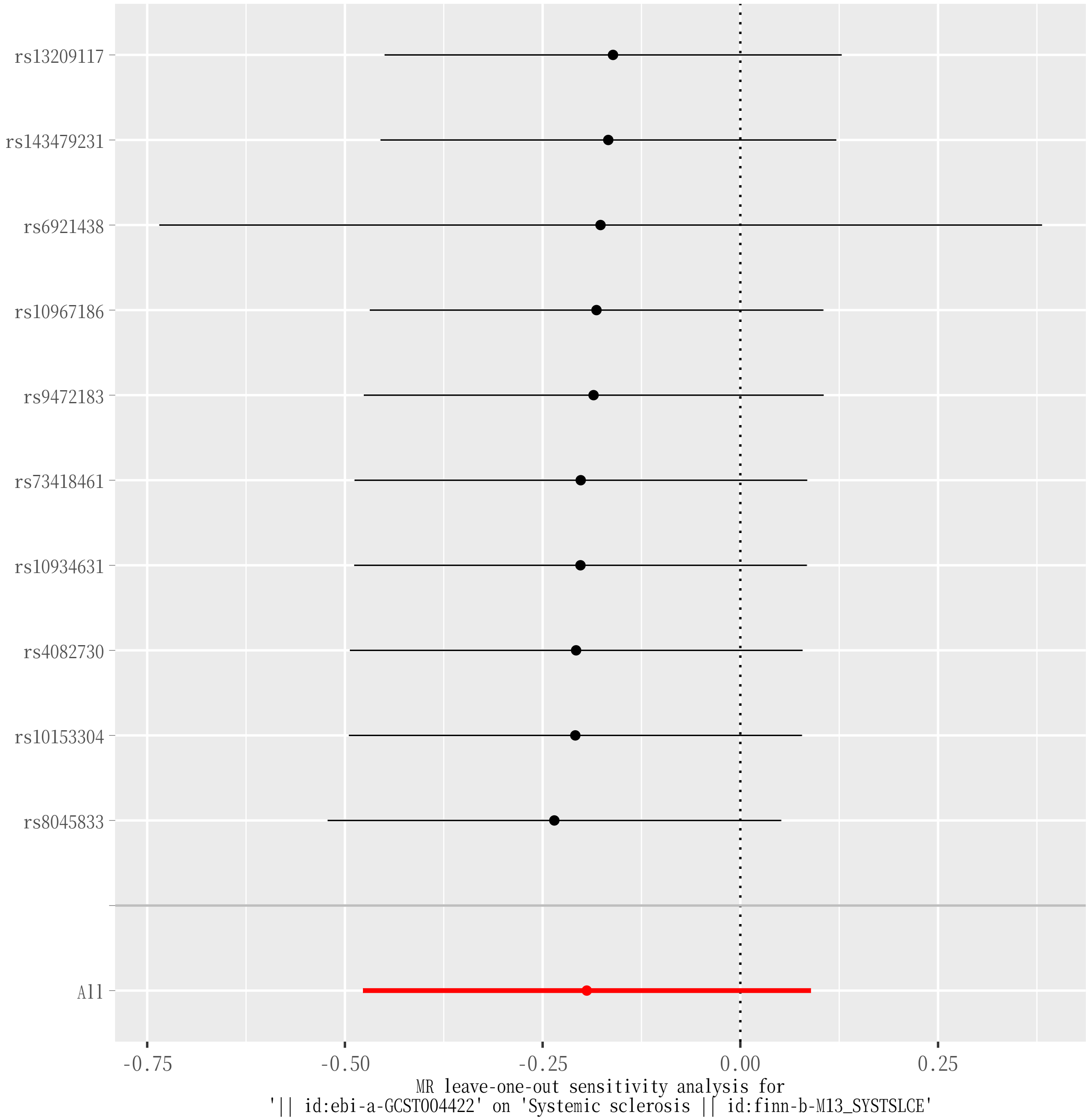
rs13209117
rs143479231
rs6921438
rs10967186
rs9472183
rs73418461
rs10934631
rs4082730
rs10153304
rs8045833
All
-0.75
-0.50
-0.25
0.00
0.25
MR leave-one-out sensitivity analysis for
'|| id:ebi-a-GCST004422' on 'Systemic sclerosis || id:finn-b-M13_SYSTSLCE'

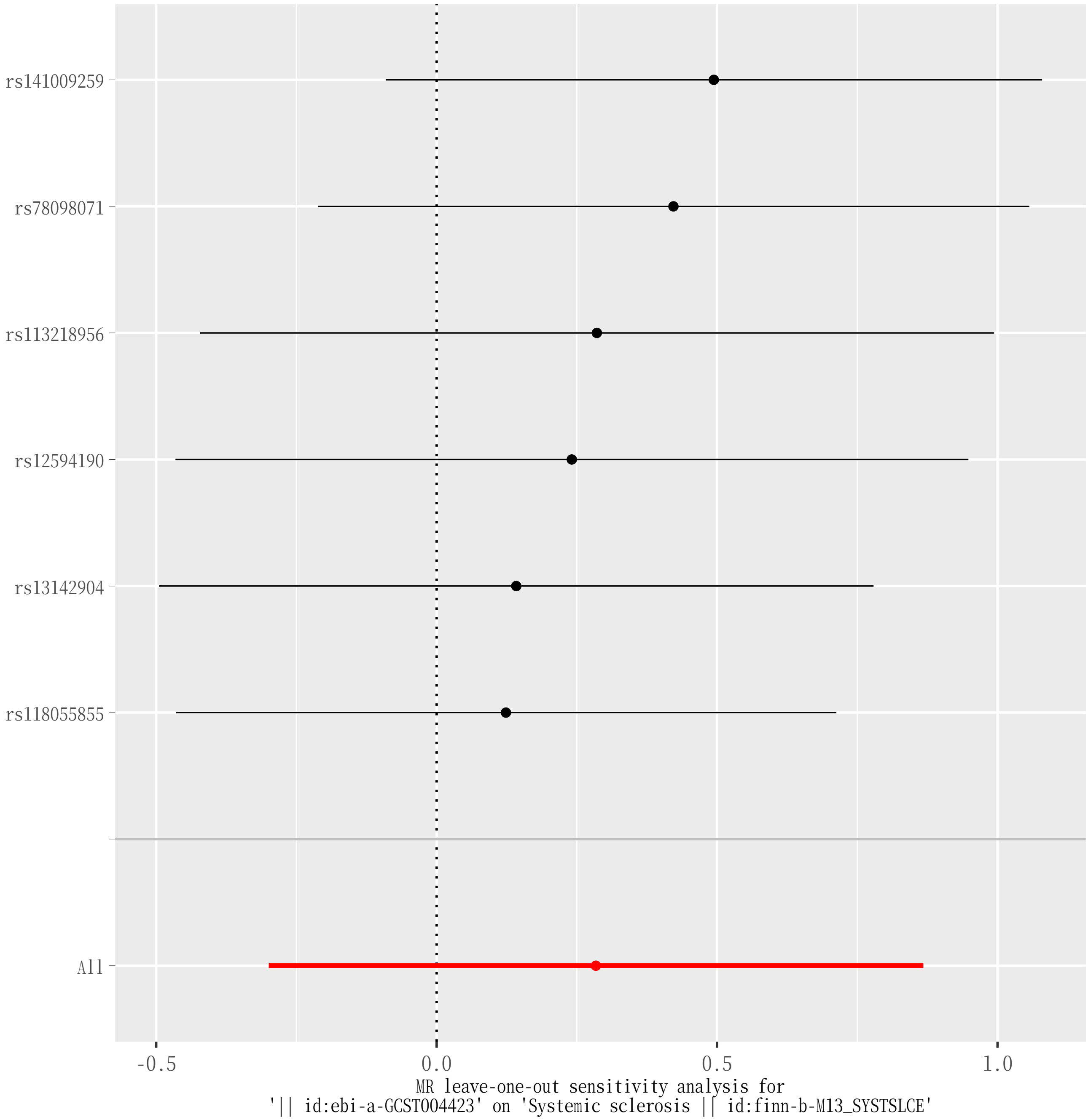
rs141009259
rs78098071
rs113218956
rs12594190
rs13142904
rs118055855
All
-0.5
0.0
0.5
1.0
MR leave-one-out sensitivity analysis for
'|| id:ebi-a-GCST004423' on 'Systemic sclerosis || id:finn-b-M13_SYSTSLCE'

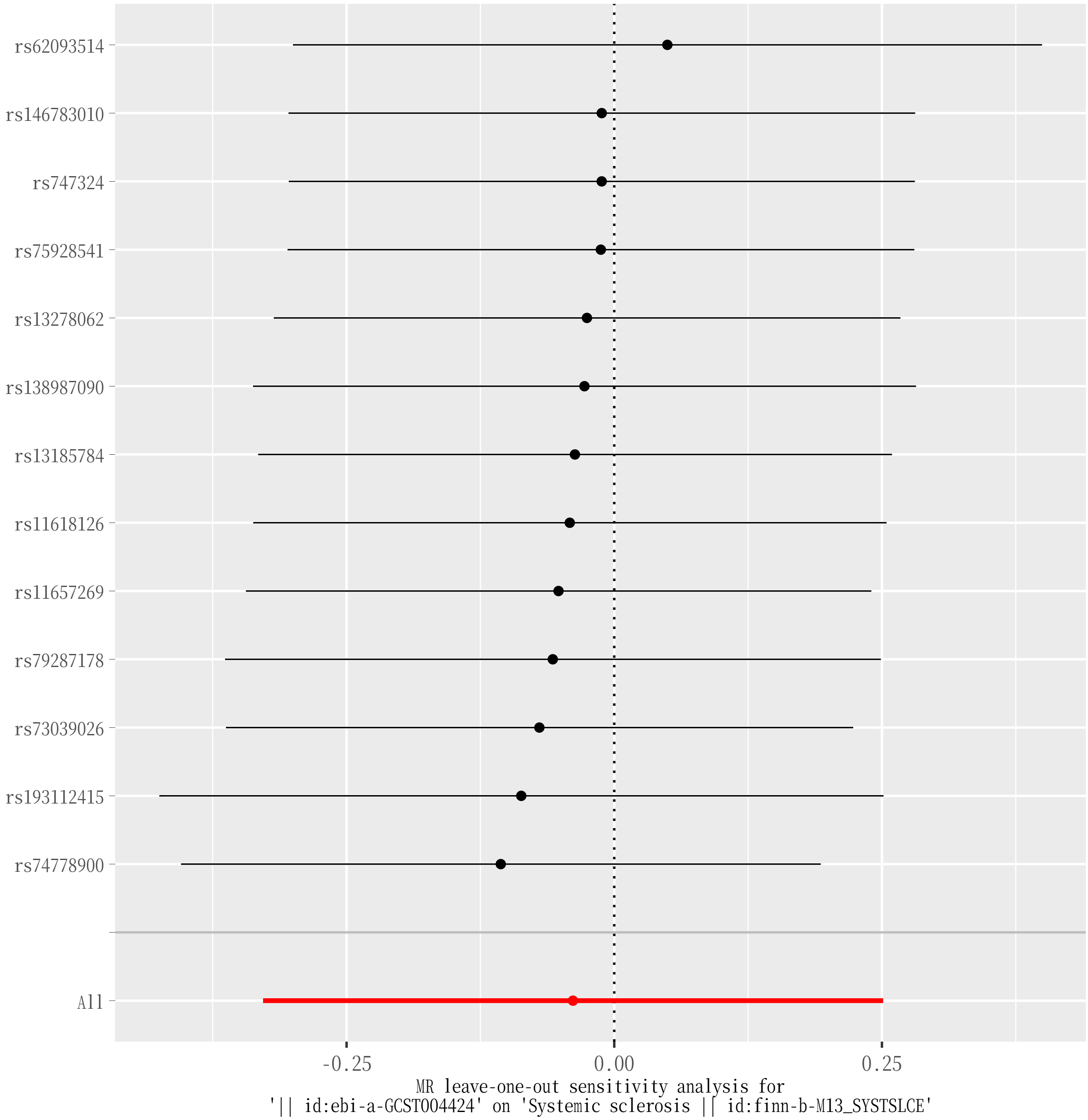
rs62093514
rs146783010
rs747324
rs75928541
rs13278062
rs138987090
rs13185784
rs11618126
rs11657269
rs79287178
rs73039026
rs193112415
rs74778900
All
-0.25
0.00
0.25
MR leave-one-out sensitivity analysis for
'|| id:ebi-a-GCST004424' on 'Systemic sclerosis || id:finn-b-M13_SYSTSLCE'

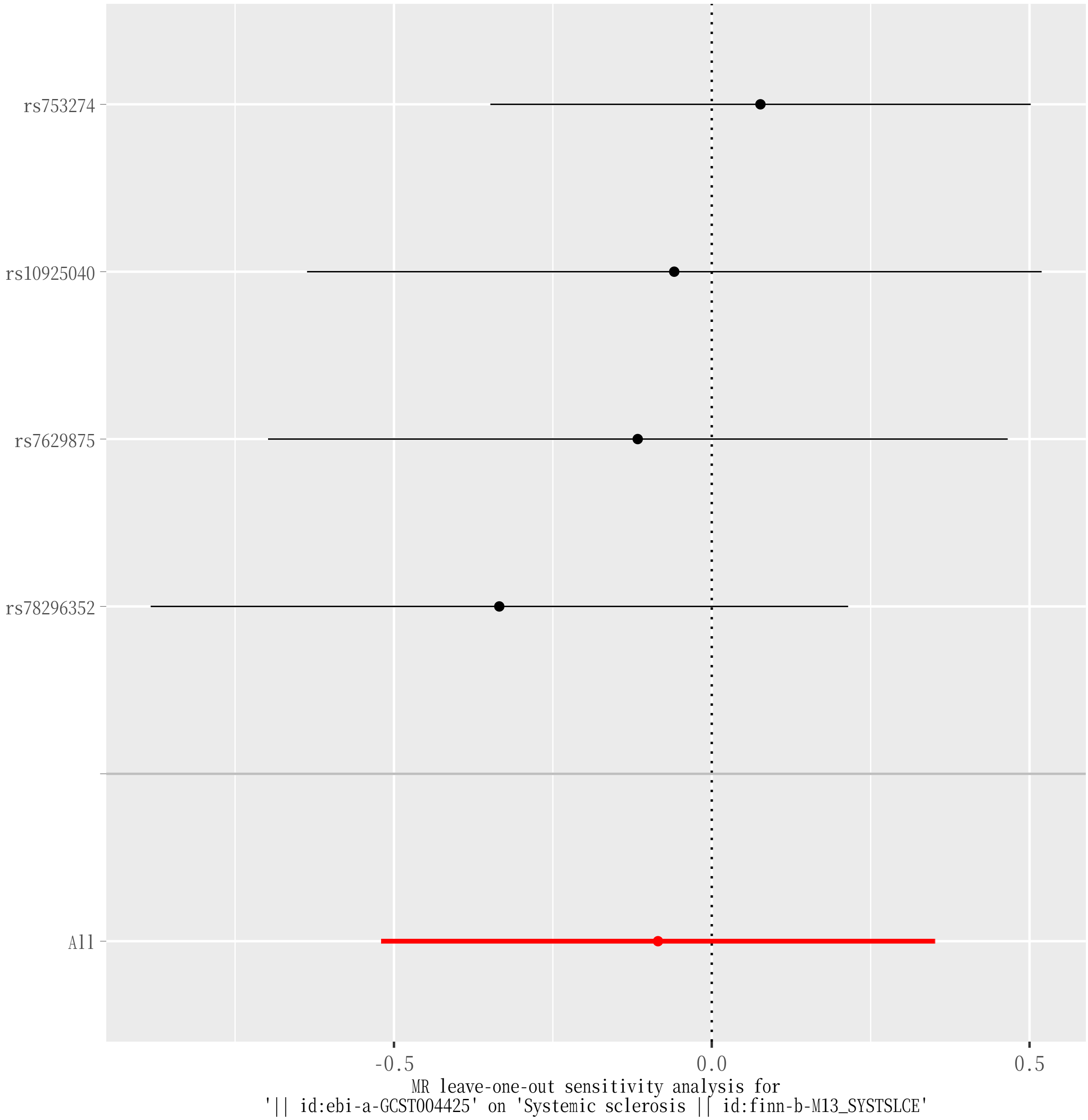
rs753274
rs10925040
rs7629875
rs78296352
All
-0.5
0.0
0.5
MR leave-one-out sensitivity analysis for
'|| id:ebi-a-GCST004425' on 'Systemic sclerosis || id:finn-b-M13_SYSTSLCE'

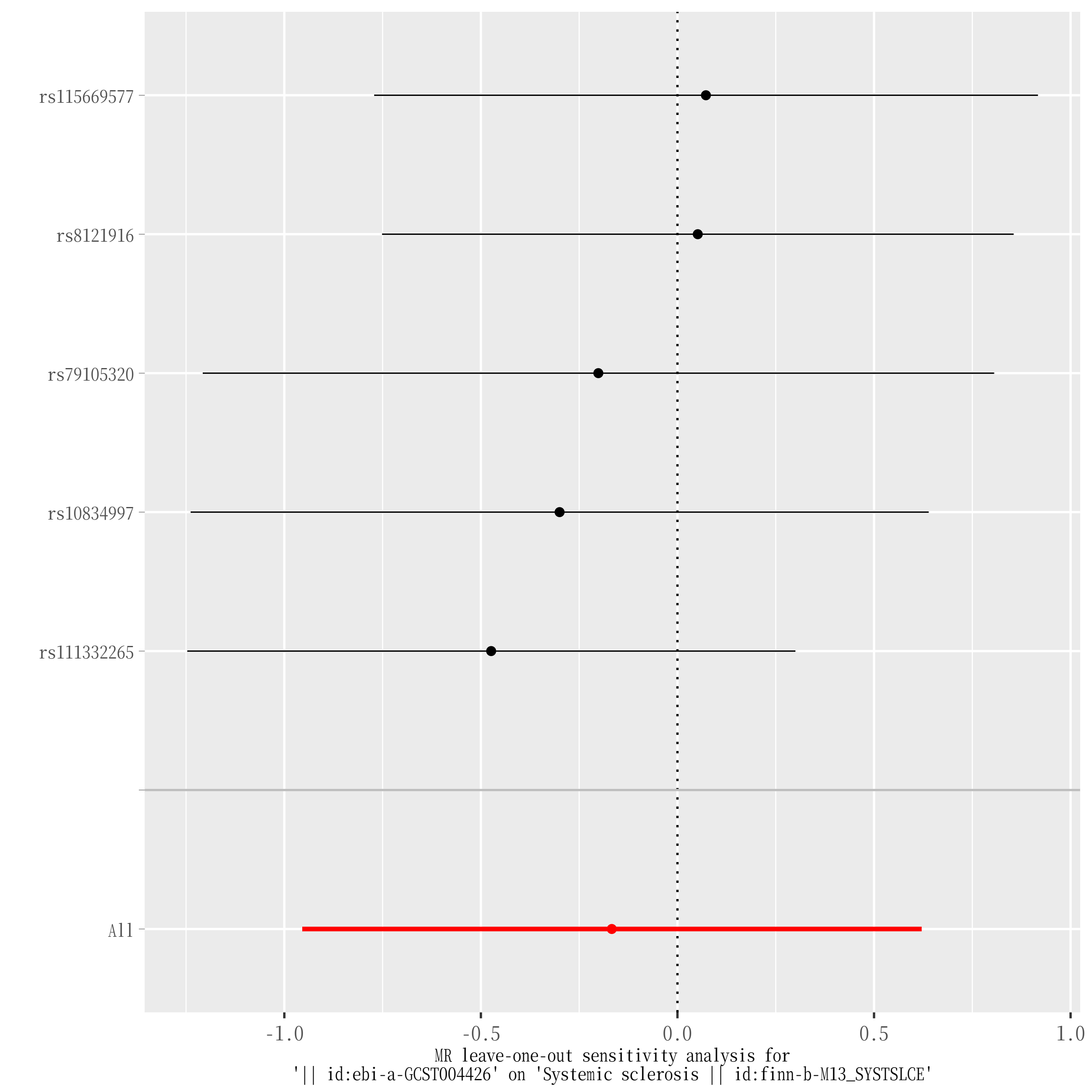
rs115669577
rs8121916
rs79105320
rs10834997
rs111332265
All
-1.0
-0.5
0.0
0.5
1.0
MR leave-one-out sensitivity analysis for
'|| id:ebi-a-GCST004426' on 'Systemic sclerosis || id:finn-b-M13_SYSTSLCE'

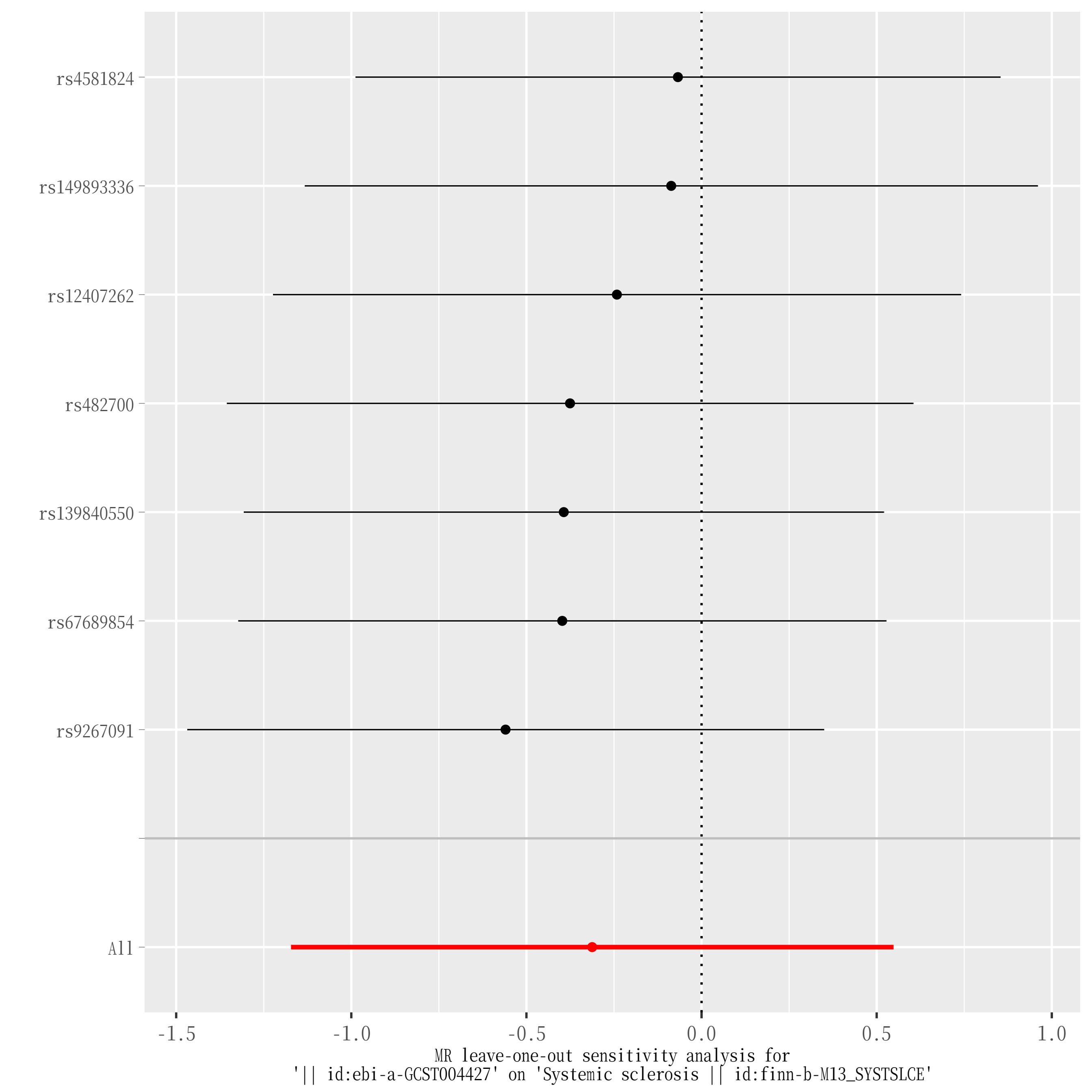

rs4581824
rs149893336
rs12407262
rs482700
rs139840550
rs67689854
rs9267091
All
-1.5
-1.0
-0.5
0.0
0.5
1.0
MR leave-one-out sensitivity analysis for
'|| id:ebi-a-GCST004427' on 'Systemic sclerosis || id:finn-b-M13_SYSTSLCE'

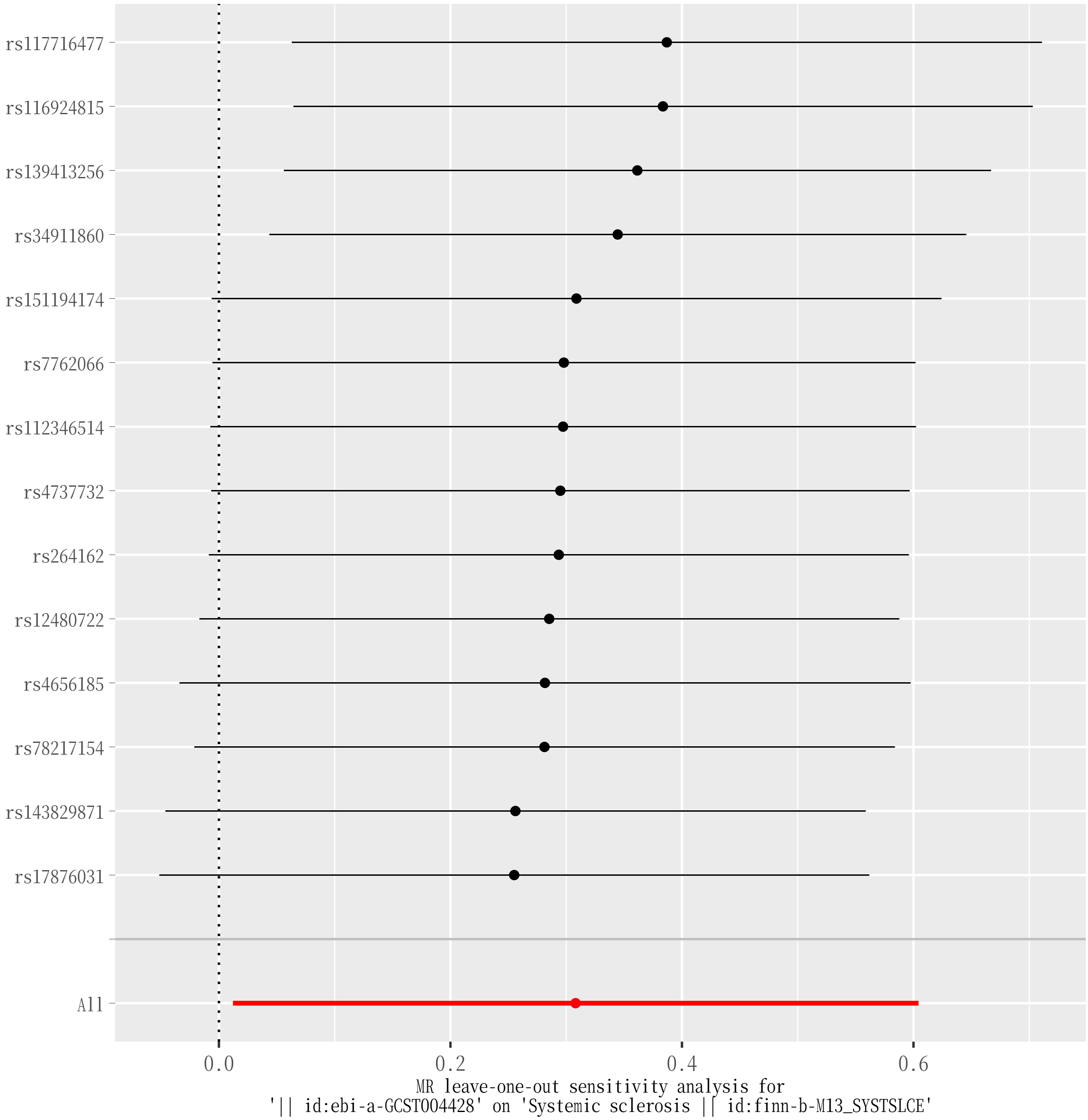
rs117716477
rs116924815
rs139413256
rs34911860
rs151194174
rs7762066
rs112346514
rs4737732
rs264162
rs12480722
rs4656185
rs78217154
rs143829871
rs17876031
All
0.0
0.2
0.4
0.6
MR leave-one-out sensitivity analysis for
'|| id:ebi-a-GCST004428' on 'Systemic sclerosis || id:finn-b-M13_SYSTSLCE'

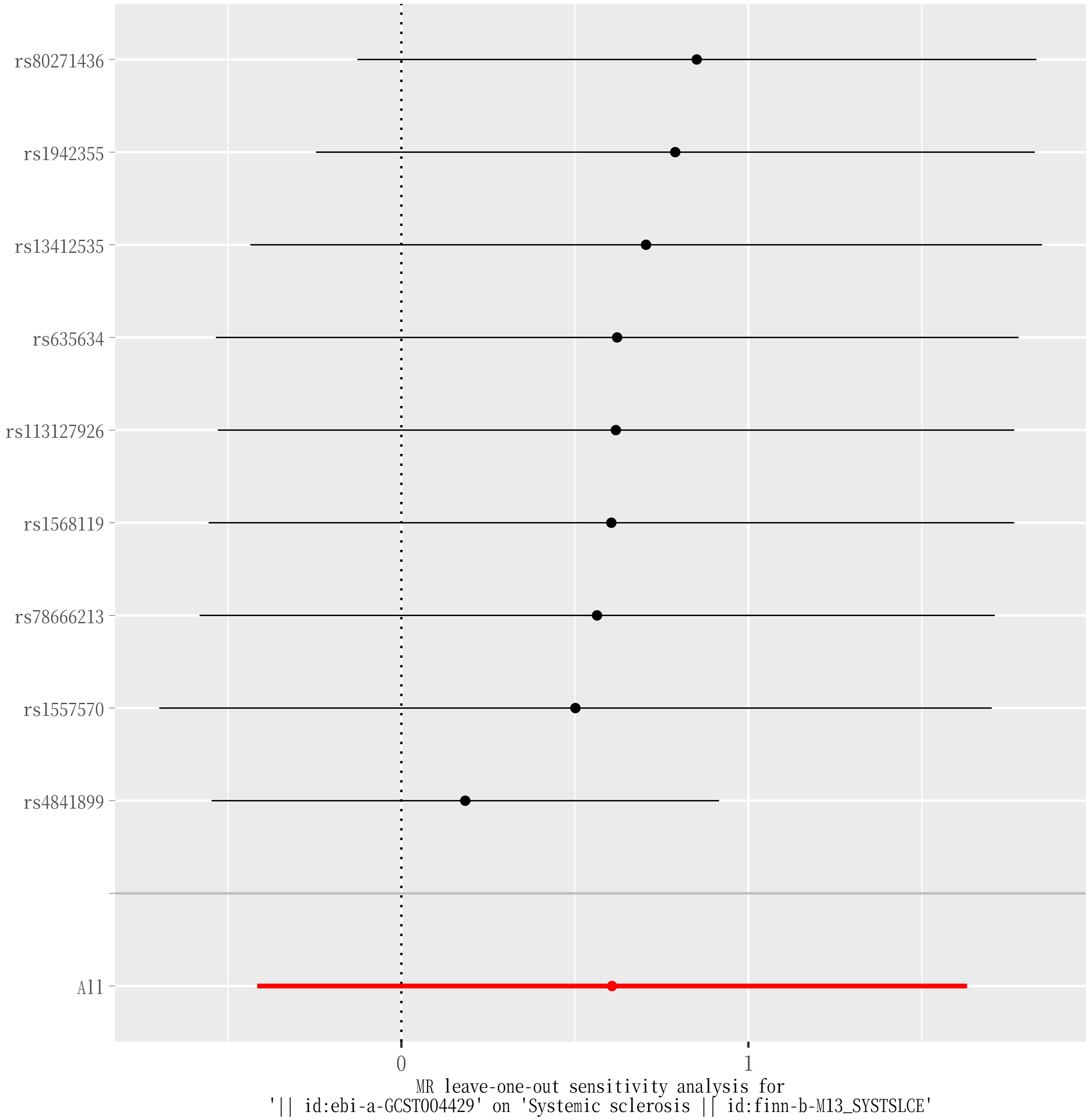

rs80271436
rs1942355
rs13412535
rs635634
rs113127926
rs1568119
rs78666213
rs1557570
rs4841899
All
0
1
MR leave-one-out sensitivity analysis for
'|| id:ebi-a-GCST004429' on 'Systemic sclerosis || id:finn-b-M13_SYSTSLCE'

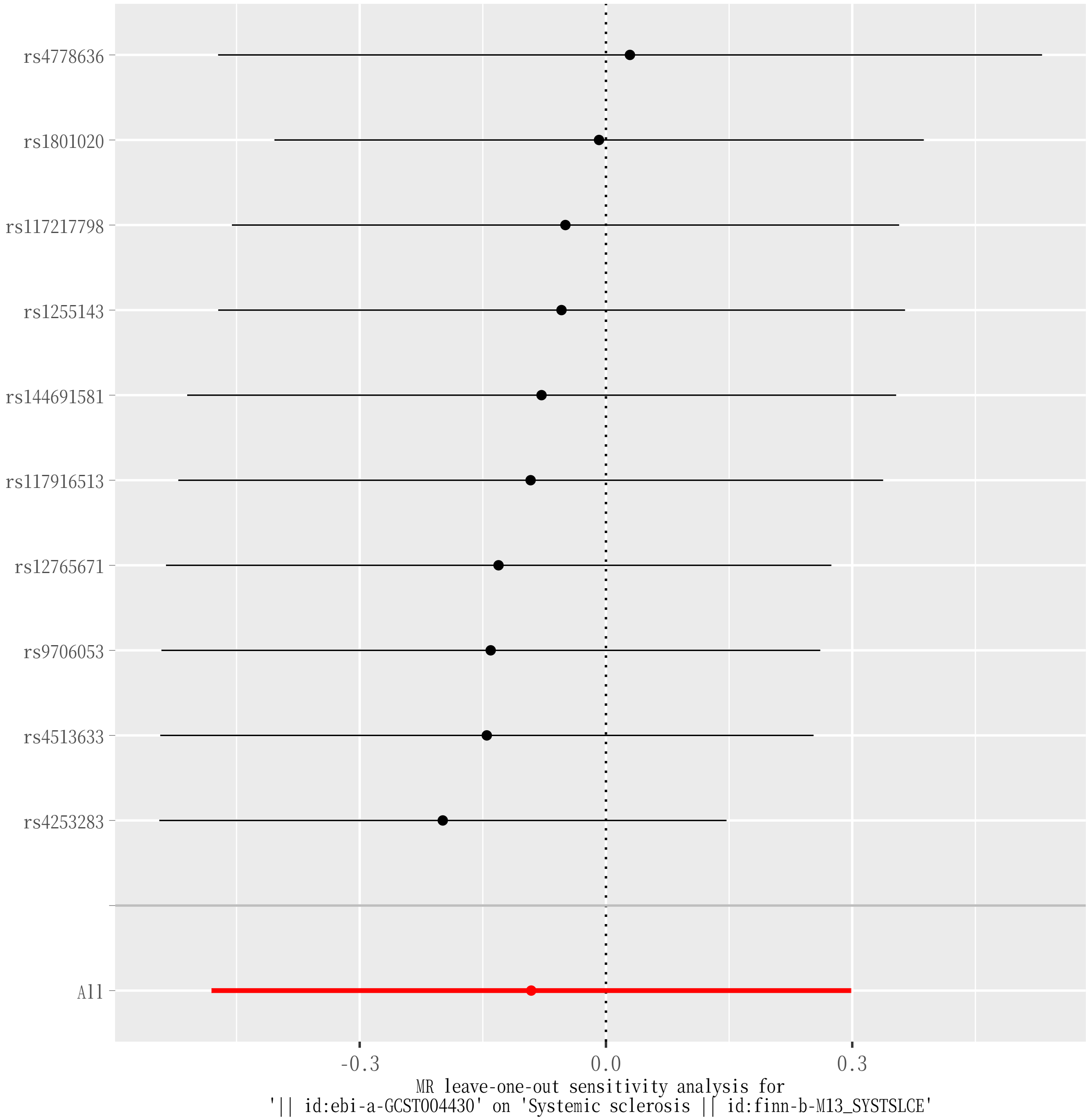
rs4778636
rs1801020
rs117217798
rs1255143
rs144691581
rs117916513
rs12765671
rs9706053
rs4513633
rs4253283
All
-0.3
0.0
0.3
MR leave-one-out sensitivity analysis for
'|| id:ebi-a-GCST004430' on 'Systemic sclerosis || id:finn-b-M13_SYSTSLCE'

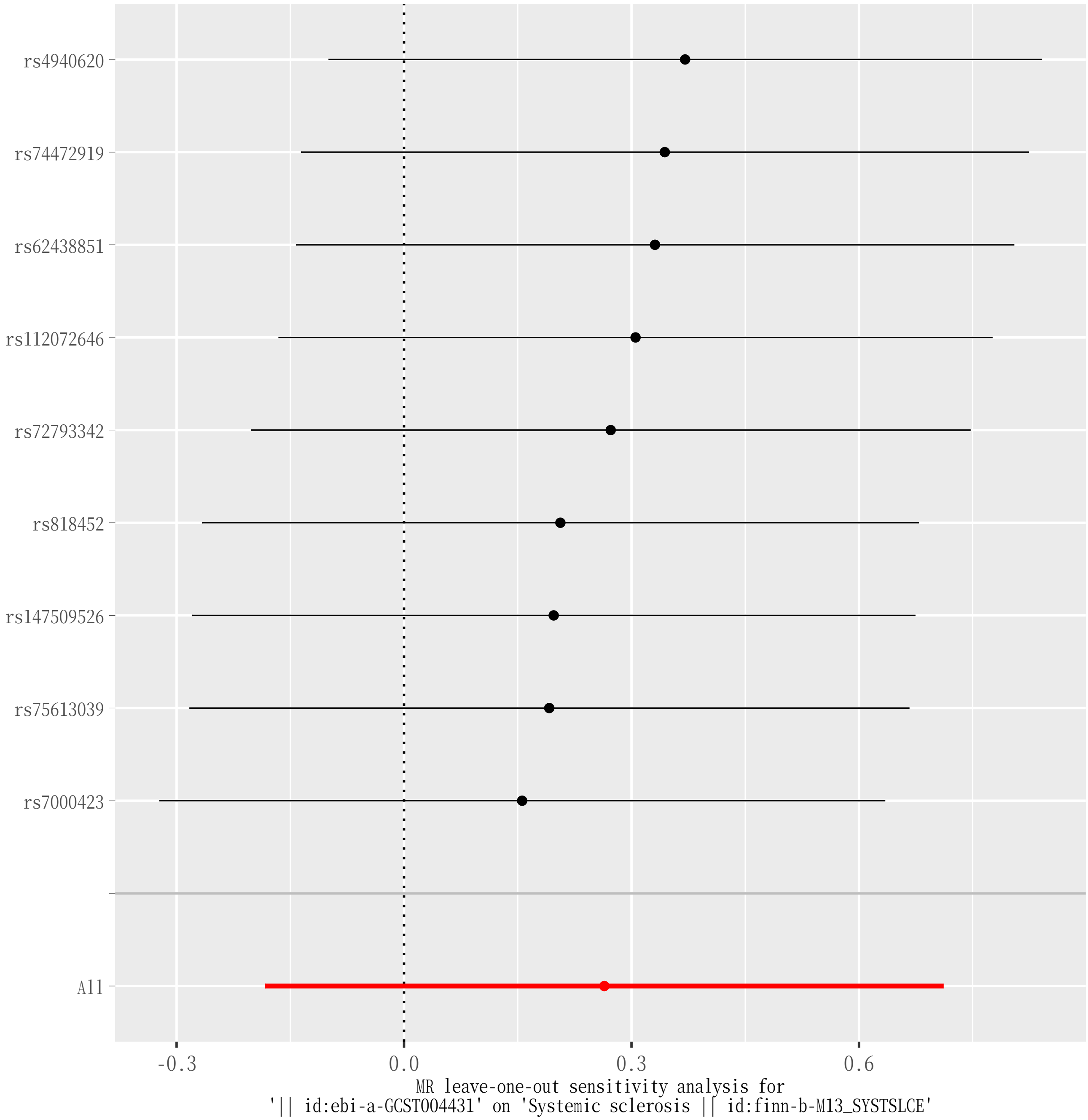
rs4940620
rs74472919
rs62438851
rs112072646
rs72793342
rs818452
rs147509526
rs75613039
rs7000423
All
-0.3
0.0
0.3
0.6
MR leave-one-out sensitivity analysis for
'|| id:ebi-a-GCST004431' on 'Systemic sclerosis || id:finn-b-M13_SYSTSLCE'

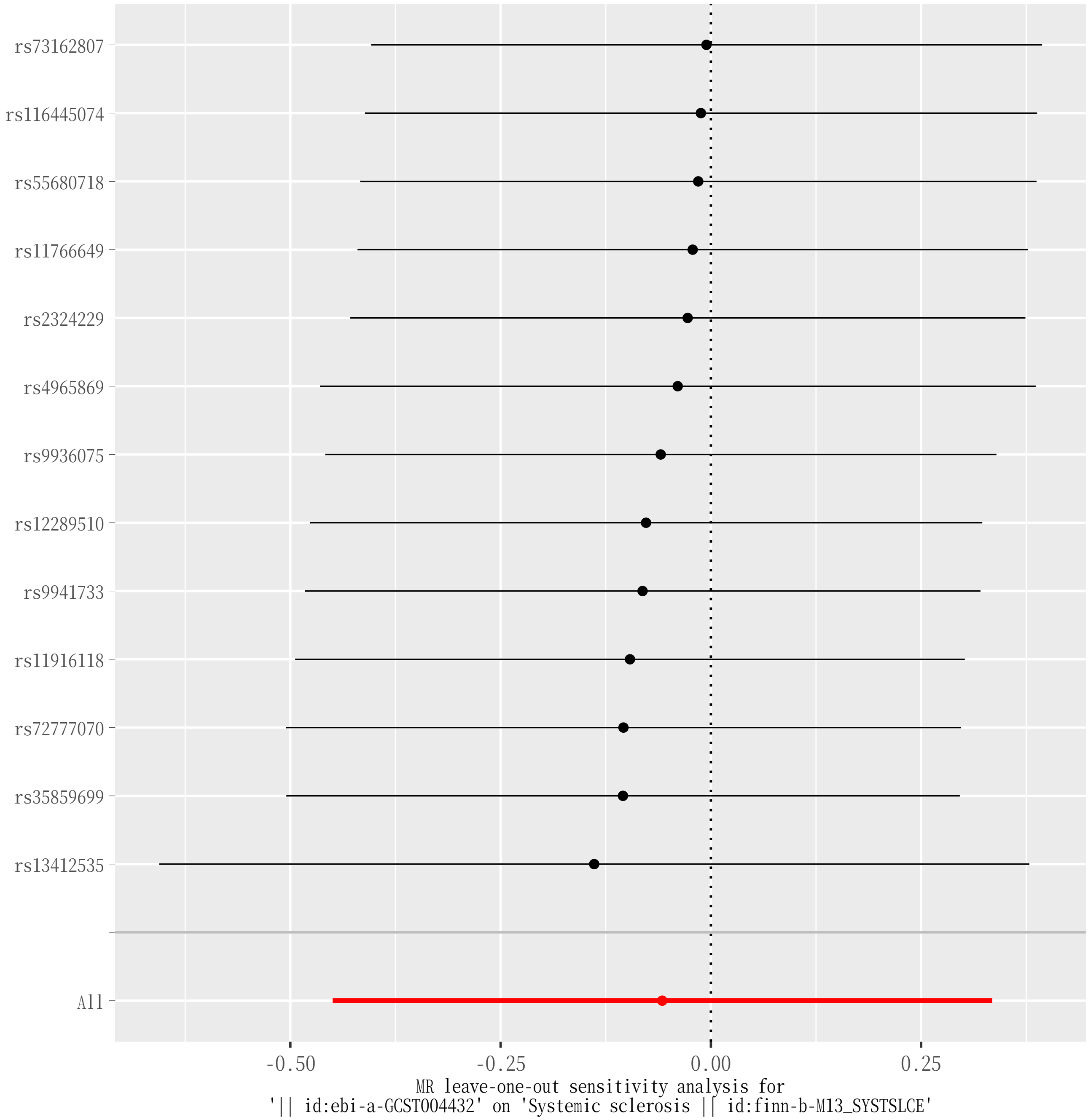
rs73162807
rs116445074
rs55680718
rs11766649
rs2324229
rs4965869
rs9936075
rs12289510
rs9941733
rs11916118
rs72777070
rs35859699
rs13412535
All
-0.50
-0.25
0.00
0.25
MR leave-one-out sensitivity analysis for
'|| id:ebi-a-GCST004432' on 'Systemic sclerosis || id:finn-b-M13_SYSTSLCE'

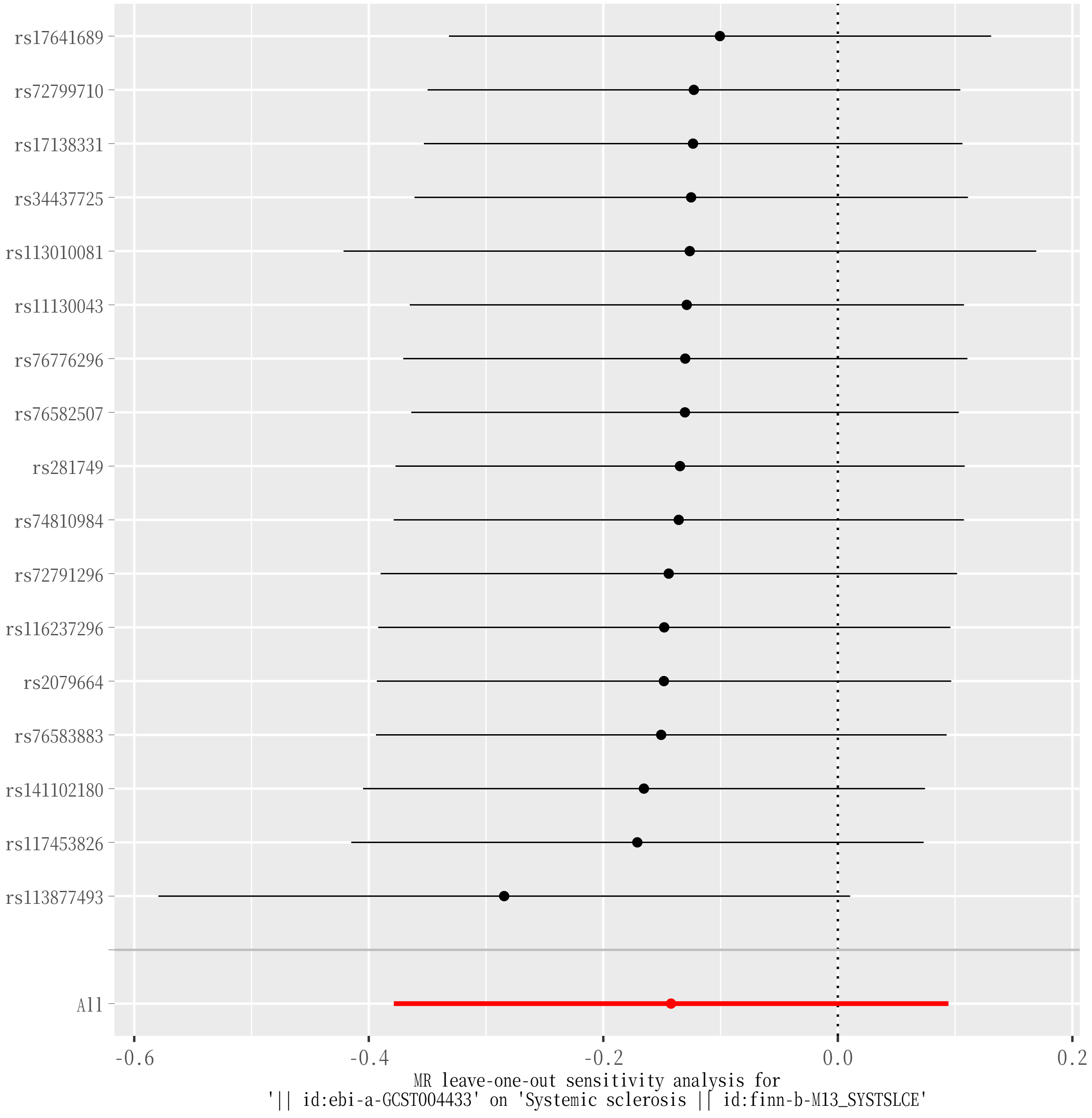
rs17641689
rs72799710
rs17138331
rs34437725
rs113010081
rs11130043
rs76776296
rs76582507
rs281749
rs74810984
rs72791296
rs116237296
rs2079664
rs76583883
rs141102180
rs117453826
rs113877493
All
-0.6
-0.4
-0.2
0.0
0.2
MR leave-one-out sensitivity analysis for
'|| id:ebi-a-GCST004433' on 'Systemic sclerosis || id:finn-b-M13_SYSTSLCE'

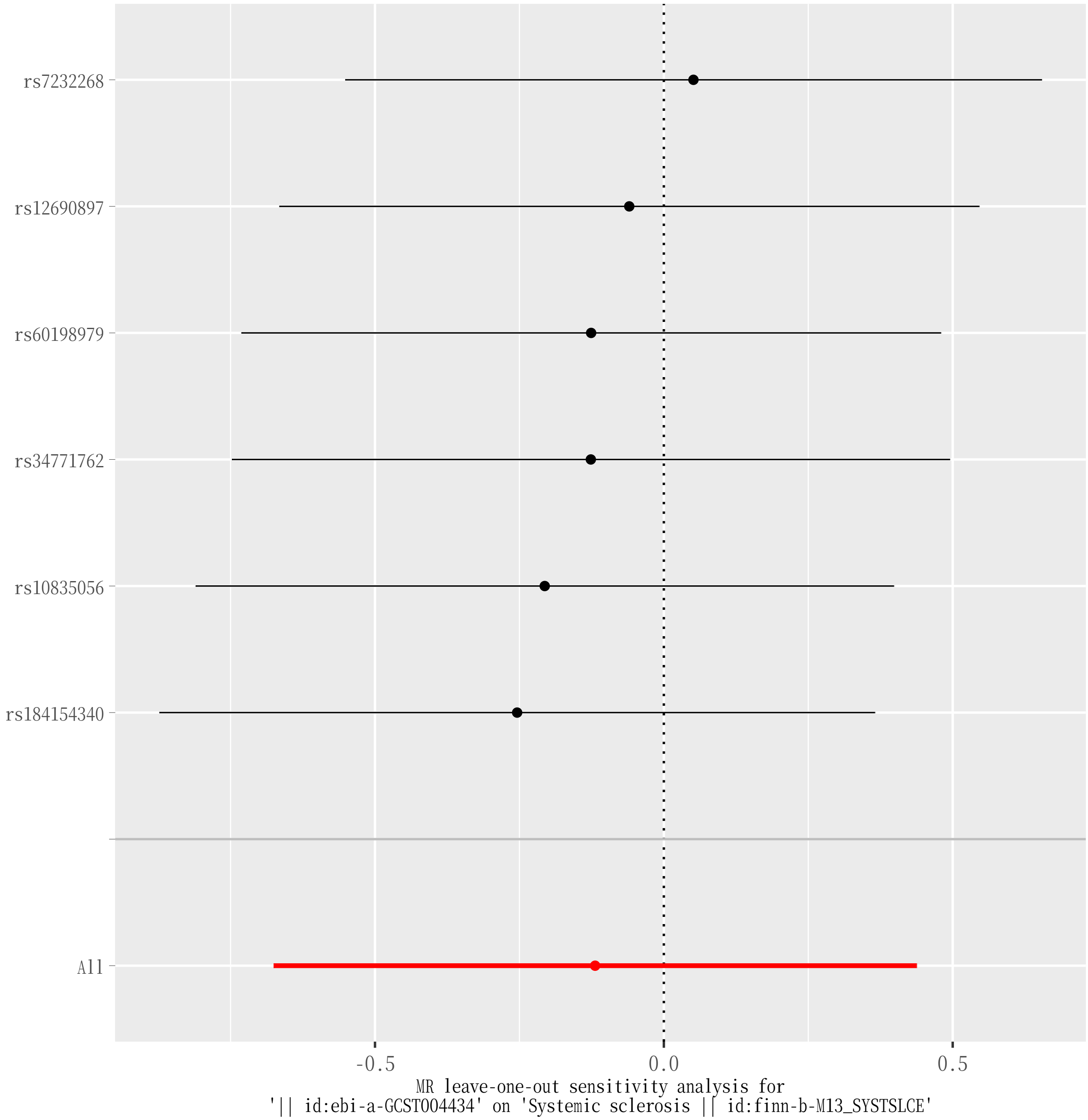

rs7232268
rs12690897
rs60198979
rs34771762
rs10835056
rs184154340
All
-0.5
0.0
0.5
MR leave-one-out sensitivity analysis for
'|| id:ebi-a-GCST004434' on 'Systemic sclerosis || id:finn-b-M13_SYSTSLCE'

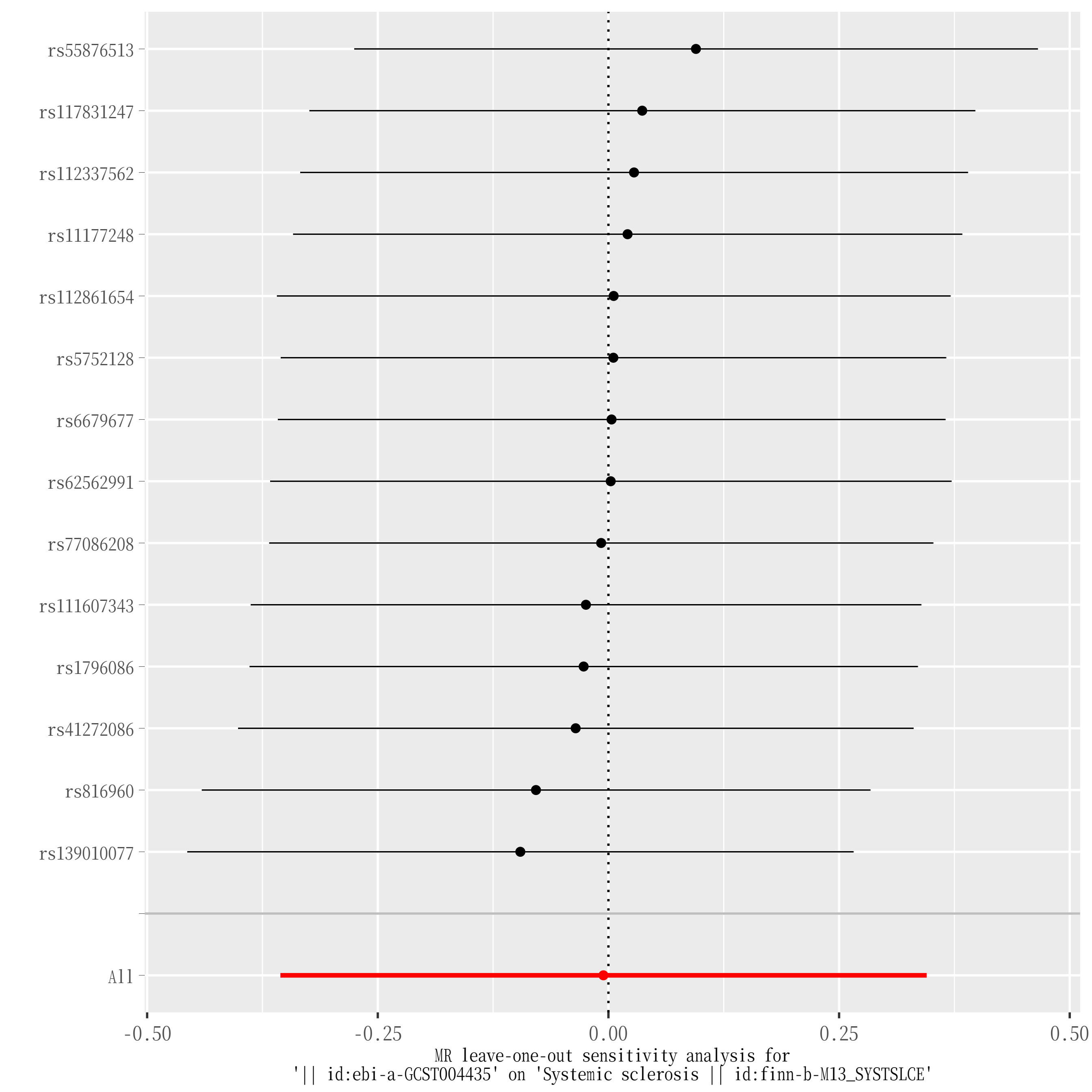
rs55876513
rs117831247
rs112337562
rs11177248
rs112861654
rs5752128
rs6679677
rs62562991
rs77086208
rs111607343
rs1796086
rs41272086
rs816960
rs139010077
All
-0.50
-0.25
0.00
0.25
0.50
MR leave-one-out sensitivity analysis for
'|| id:ebi-a-GCST004435' on 'Systemic sclerosis || id:finn-b-M13_SYSTSLCE'

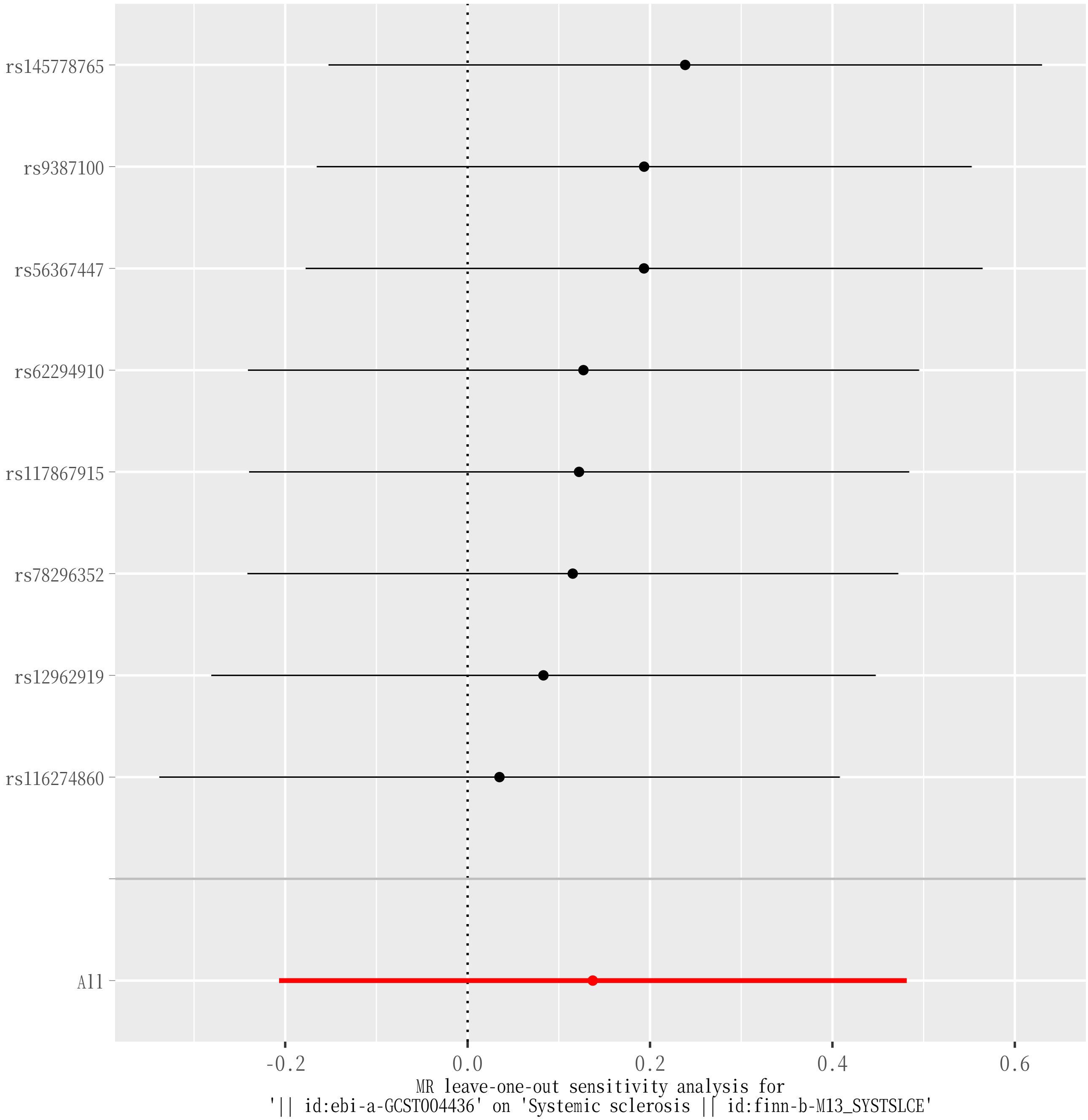

rs145778765
rs9387100
rs56367447
rs62294910
rs117867915
rs78296352
rs12962919
rs116274860
All
-0.2
0.0
0.2
0.4
0.6
MR leave-one-out sensitivity analysis for
'|| id:ebi-a-GCST004436' on 'Systemic sclerosis || id:finn-b-M13_SYSTSLCE'

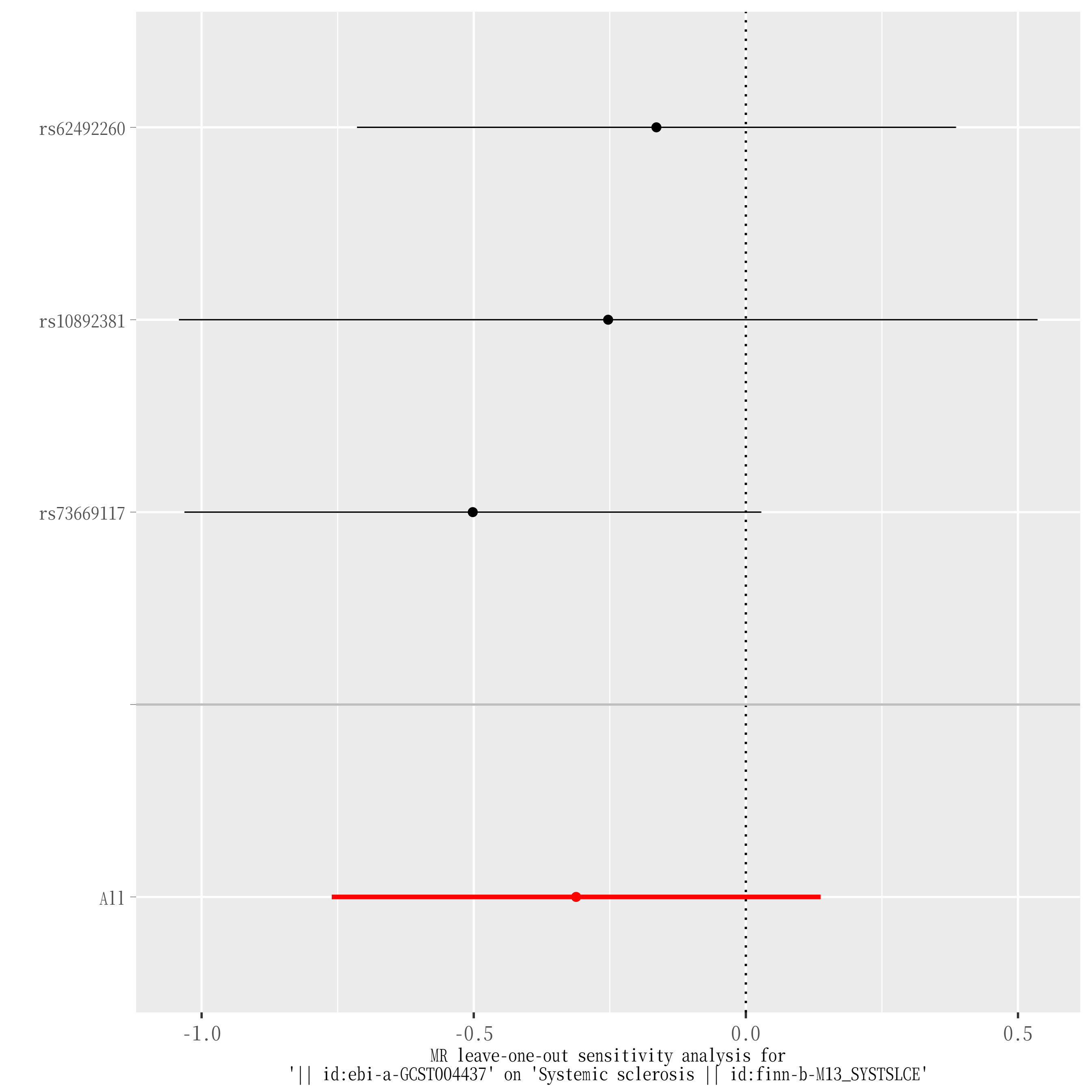

rs62492260
rs10892381
rs73669117
All
-1.0
-0.5
0.0
0.5
MR leave-one-out sensitivity analysis for
'|| id:ebi-a-GCST004437' on 'Systemic sclerosis || id:finn-b-M13_SYSTSLCE'

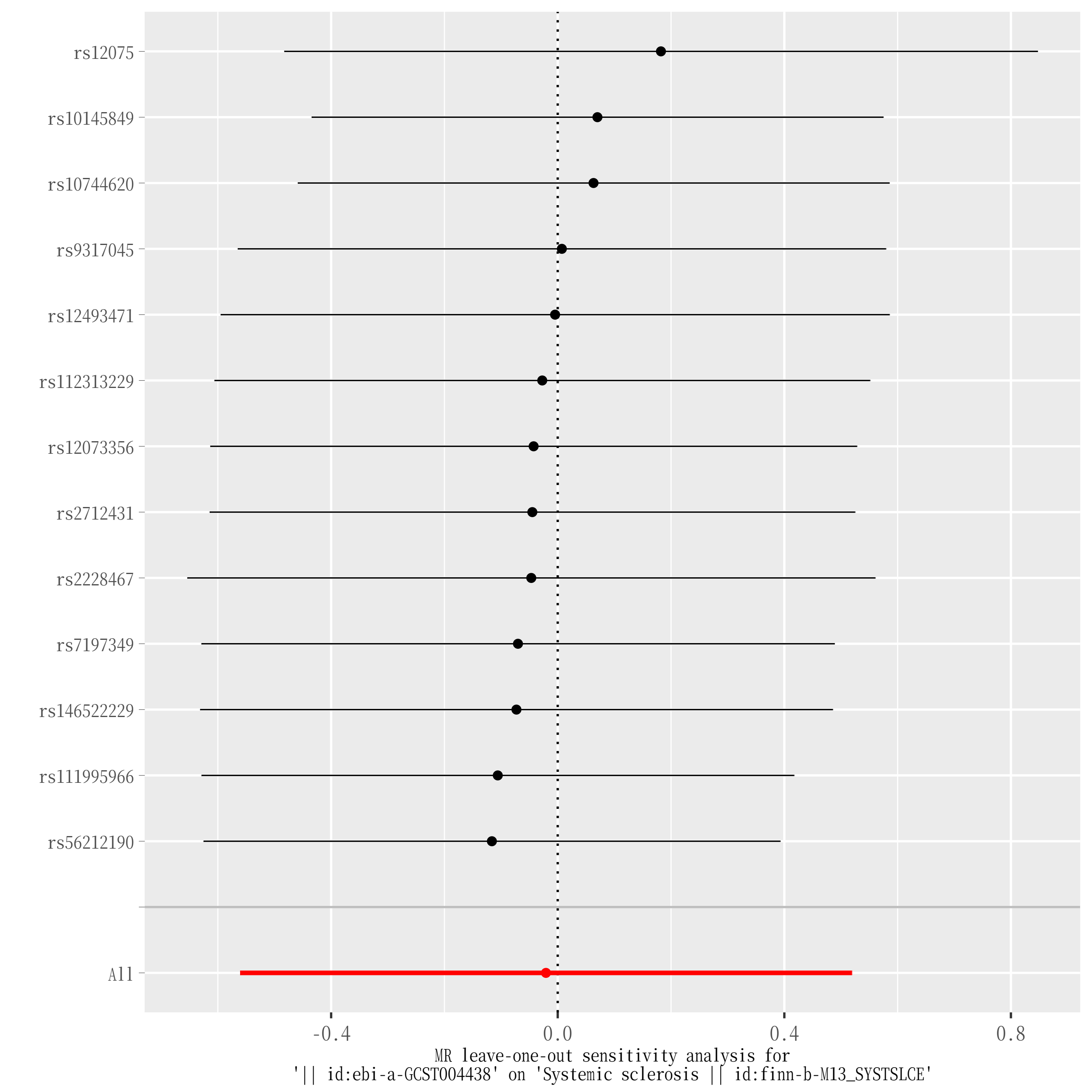
rs12075
rs10145849
rs10744620
rs9317045
rs12493471
rs112313229
rs12073356
rs2712431
rs2228467
rs7197349
rs146522229
rs111995966
rs56212190
All
-0.4
0.0
0.4
0.8
MR leave-one-out sensitivity analysis for
'|| id:ebi-a-GCST004438' on 'Systemic sclerosis || id:finn-b-M13_SYSTSLCE'

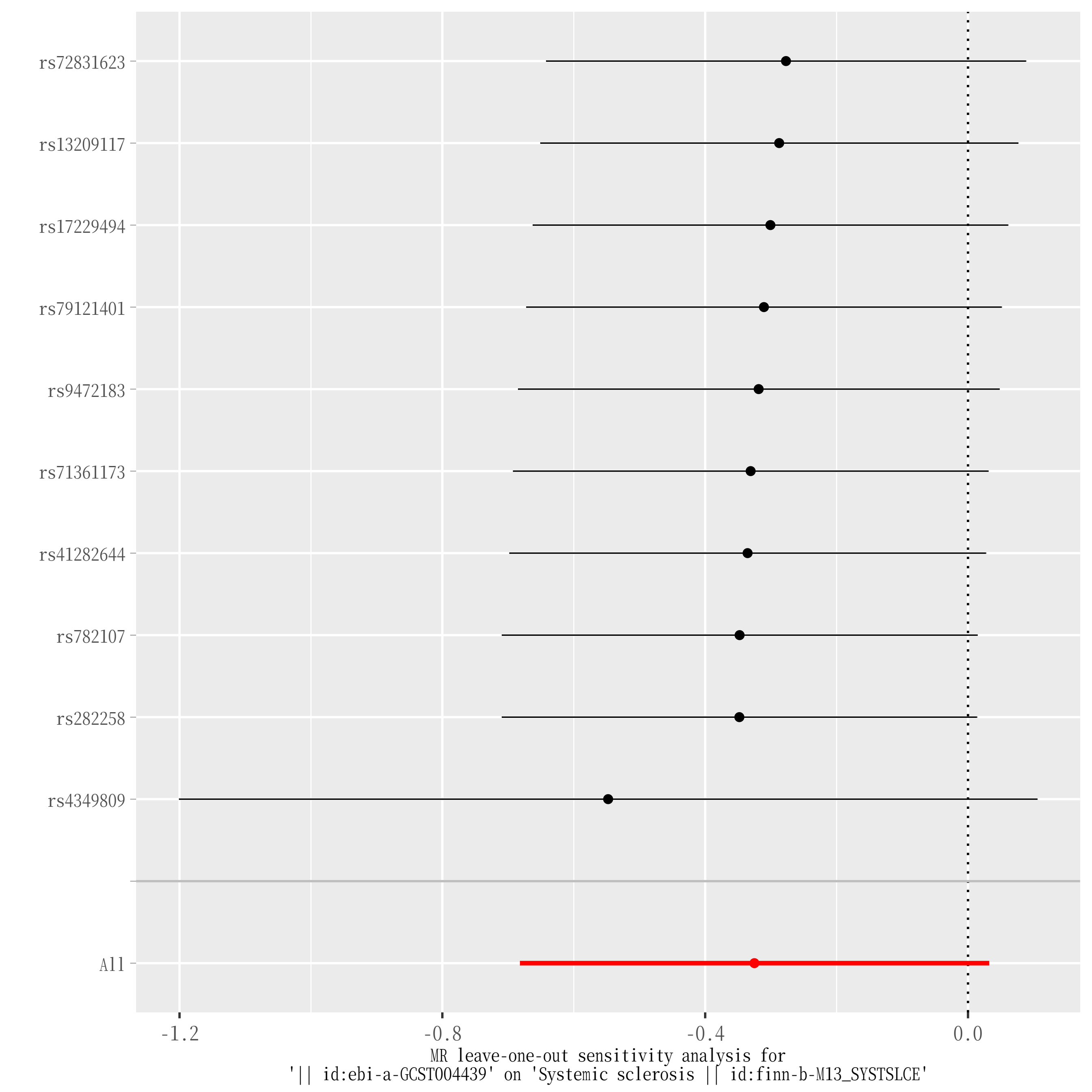
rs72831623
rs13209117
rs17229494
rs79121401
rs9472183
rs71361173
rs41282644
rs782107
rs282258
rs4349809
All
-1.2
-0.8
-0.4
0.0
MR leave-one-out sensitivity analysis for
'|| id:ebi-a-GCST004439' on 'Systemic sclerosis || id:finn-b-M13_SYSTSLCE'

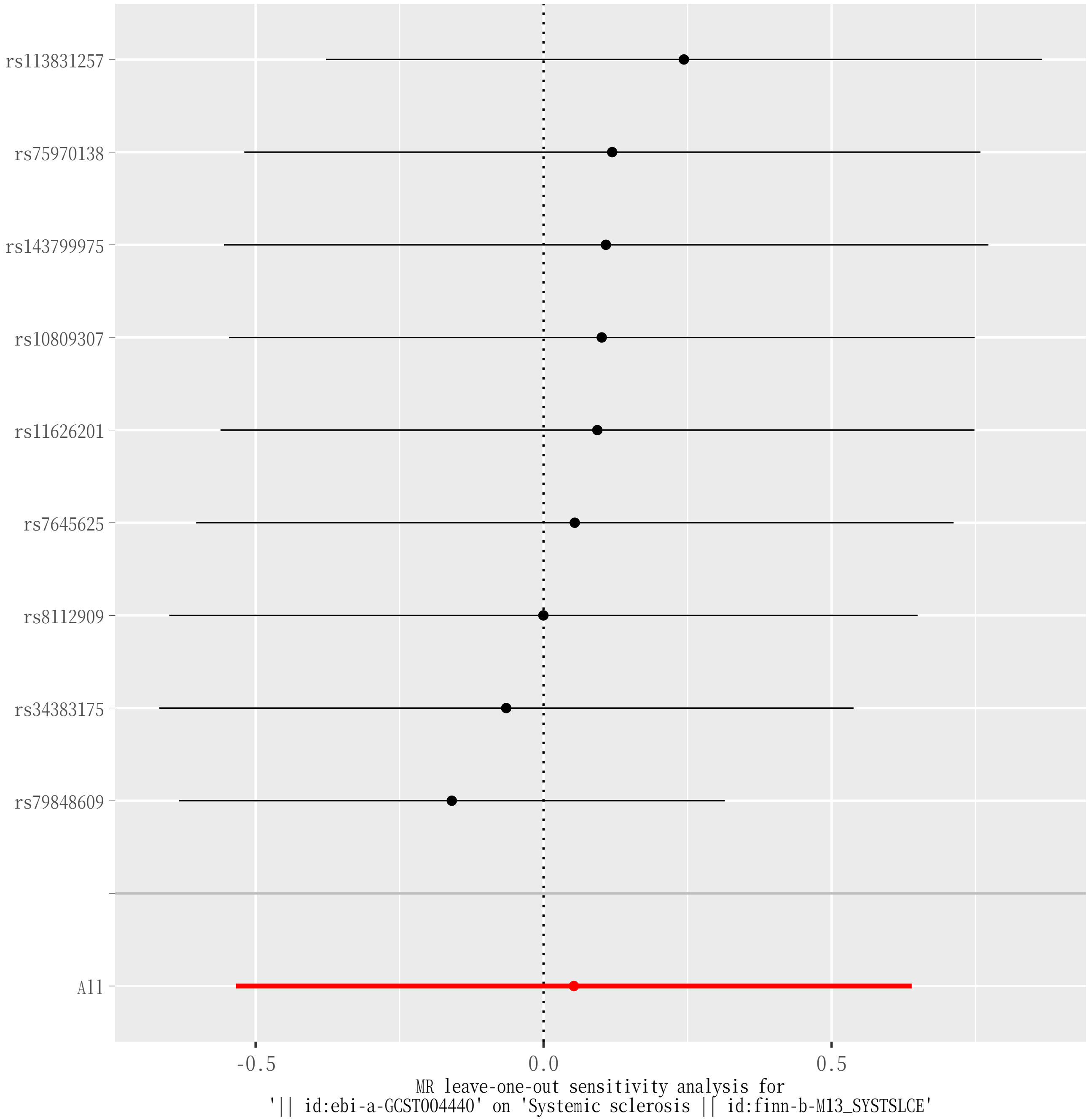
rs113831257
rs75970138
rs143799975
rs10809307
rs11626201
rs7645625
rs8112909
rs34383175
rs79848609
All
-0.5
0.0
0.5
MR leave-one-out sensitivity analysis for
'|| id:ebi-a-GCST004440' on 'Systemic sclerosis || id:finn-b-M13_SYSTSLCE'

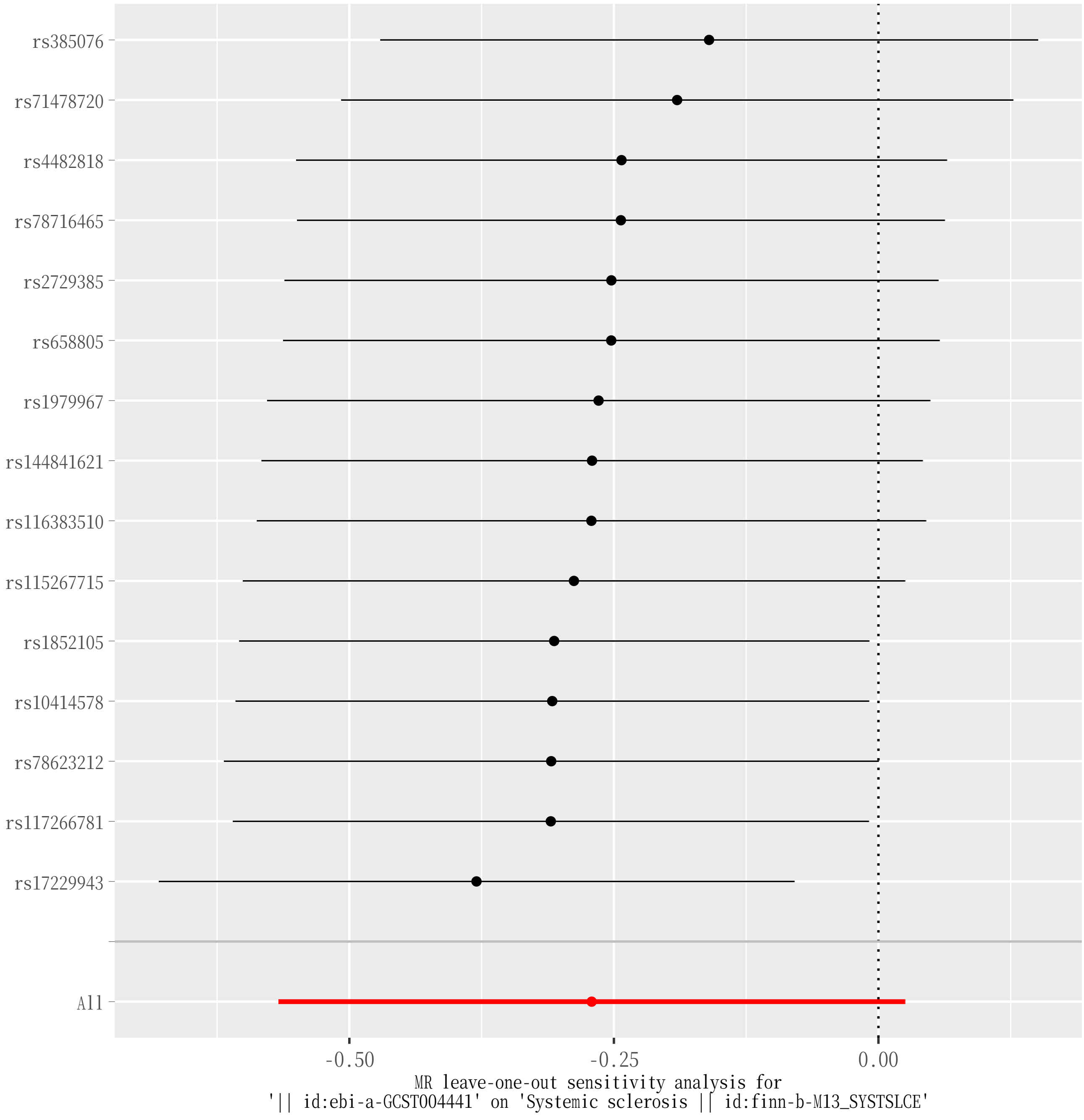

rs385076
rs71478720
rs4482818
rs78716465
rs2729385
rs658805
rs1979967
rs144841621
rs116383510
rs115267715
rs1852105
rs10414578
rs78623212
rs117266781
rs17229943
All
-0.50
-0.25
0.00
MR leave-one-out sensitivity analysis for
'|| id:ebi-a-GCST004441' on 'Systemic sclerosis || id:finn-b-M13_SYSTSLCE'

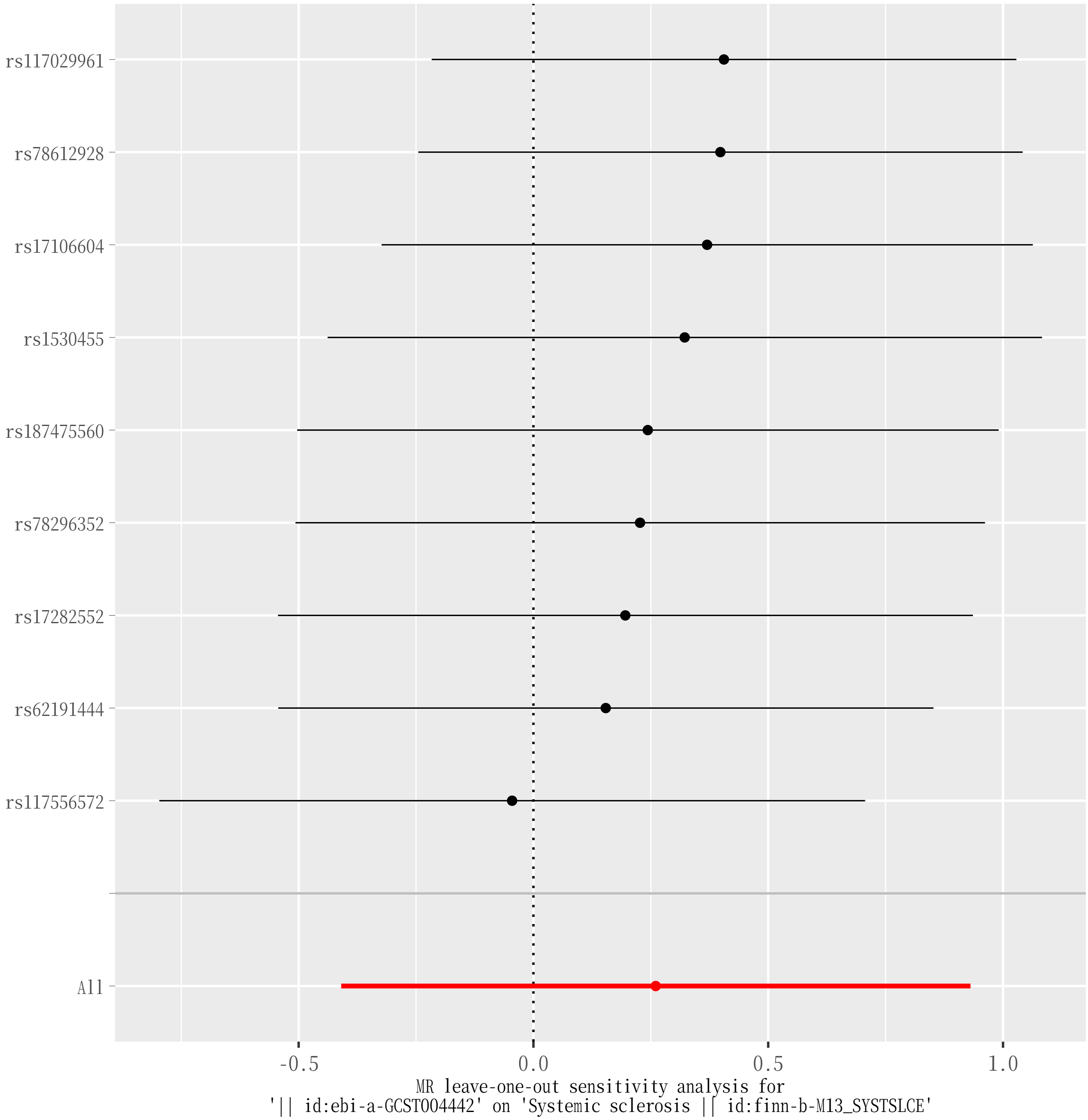
rs117029961
rs78612928
rs17106604
rs1530455
rs187475560
rs78296352
rs17282552
rs62191444
rs117556572
All
-0.5
0.0
0.5
1.0
MR leave-one-out sensitivity analysis for
'|| id:ebi-a-GCST004442' on 'Systemic sclerosis || id:finn-b-M13_SYSTSLCE'

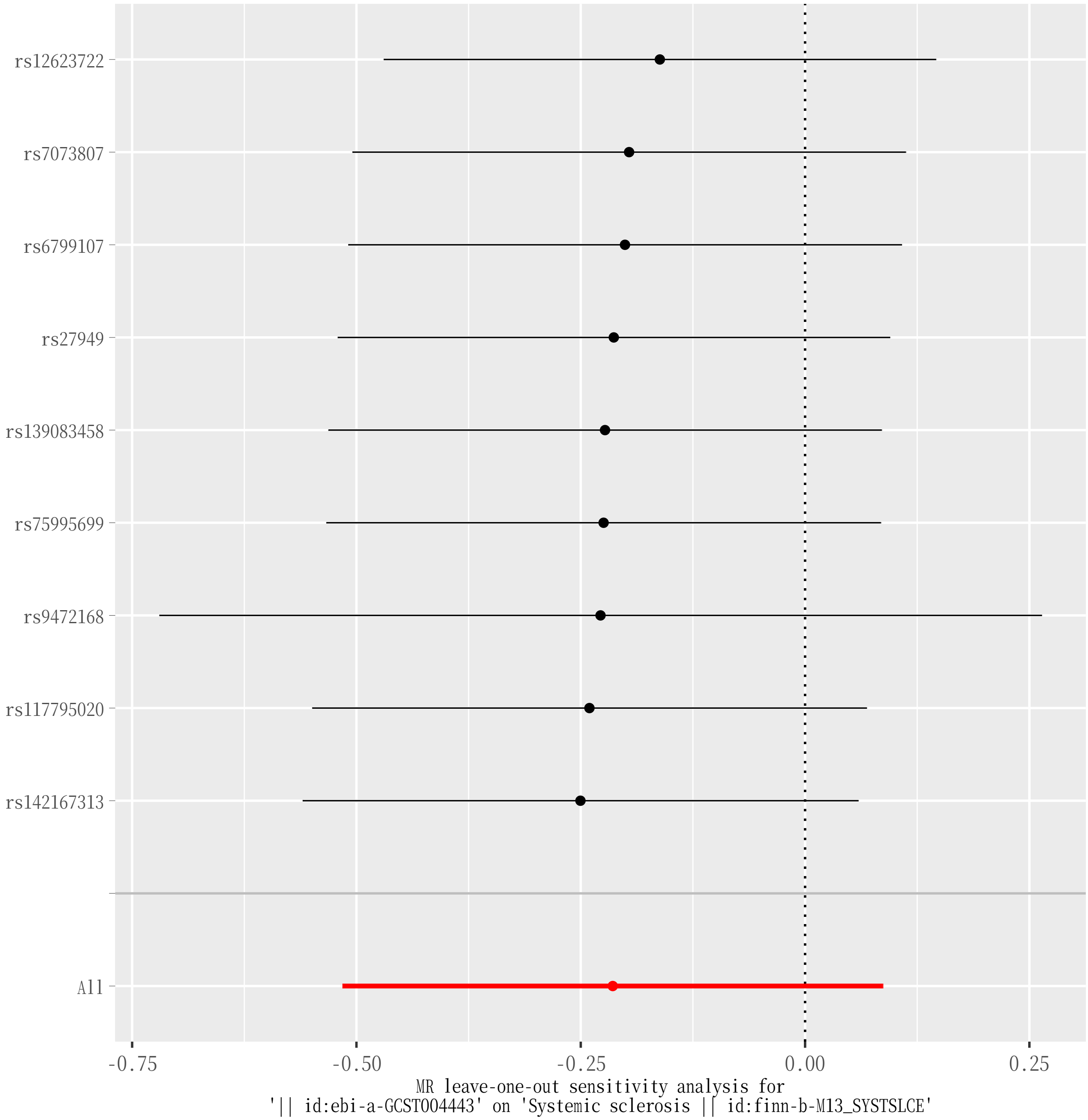
rs12623722
rs7073807
rs6799107
rs27949
rs139083458
rs75995699
rs9472168
rs117795020
rs142167313
All
-0.75
-0.50
-0.25
0.00
0.25
MR leave-one-out sensitivity analysis for
'|| id:ebi-a-GCST004443' on 'Systemic sclerosis || id:finn-b-M13_SYSTSLCE'

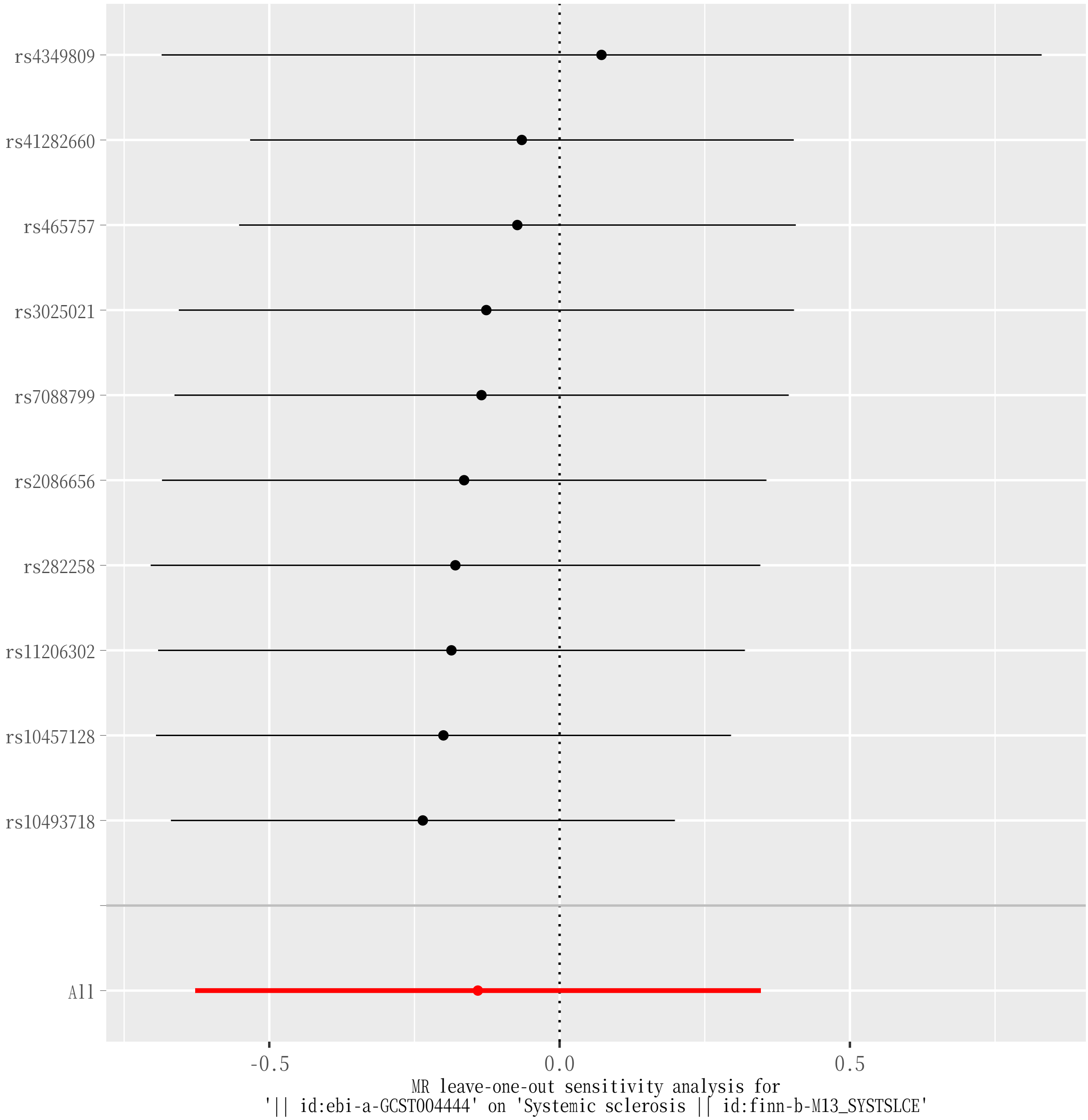

rs4349809
rs41282660
rs465757
rs3025021
rs7088799
rs2086656
rs282258
rs11206302
rs10457128
rs10493718
All
-0.5
0.0
0.5
MR leave-one-out sensitivity analysis for
'|| id:ebi-a-GCST004444' on 'Systemic sclerosis || id:finn-b-M13_SYSTSLCE'

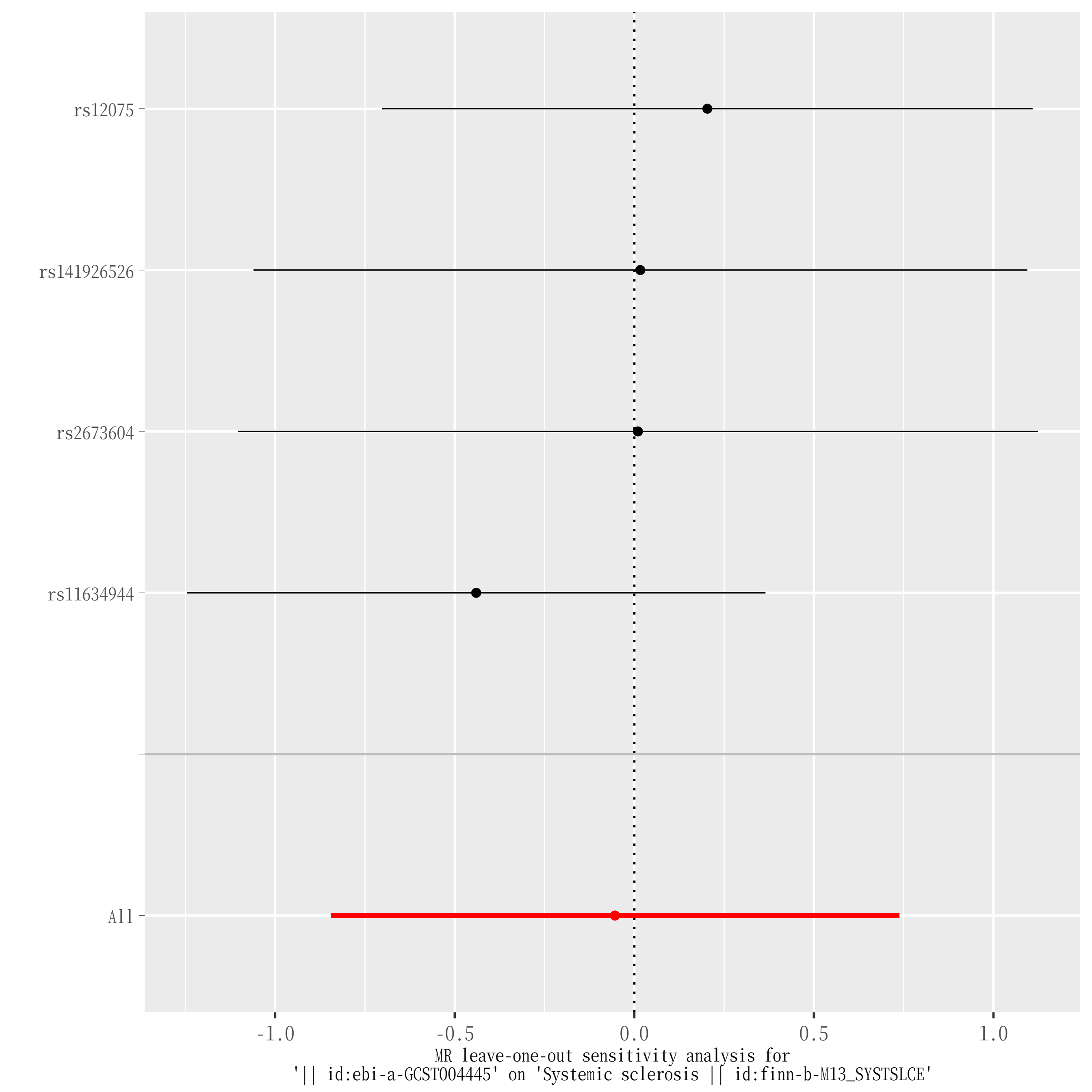
rs12075
rs141926526
rs2673604
rs11634944
All
-1.0
-0.5
0.0
0.5
1.0
MR leave-one-out sensitivity analysis for
'|| id:ebi-a-GCST004445' on 'Systemic sclerosis || id:finn-b-M13_SYSTSLCE'

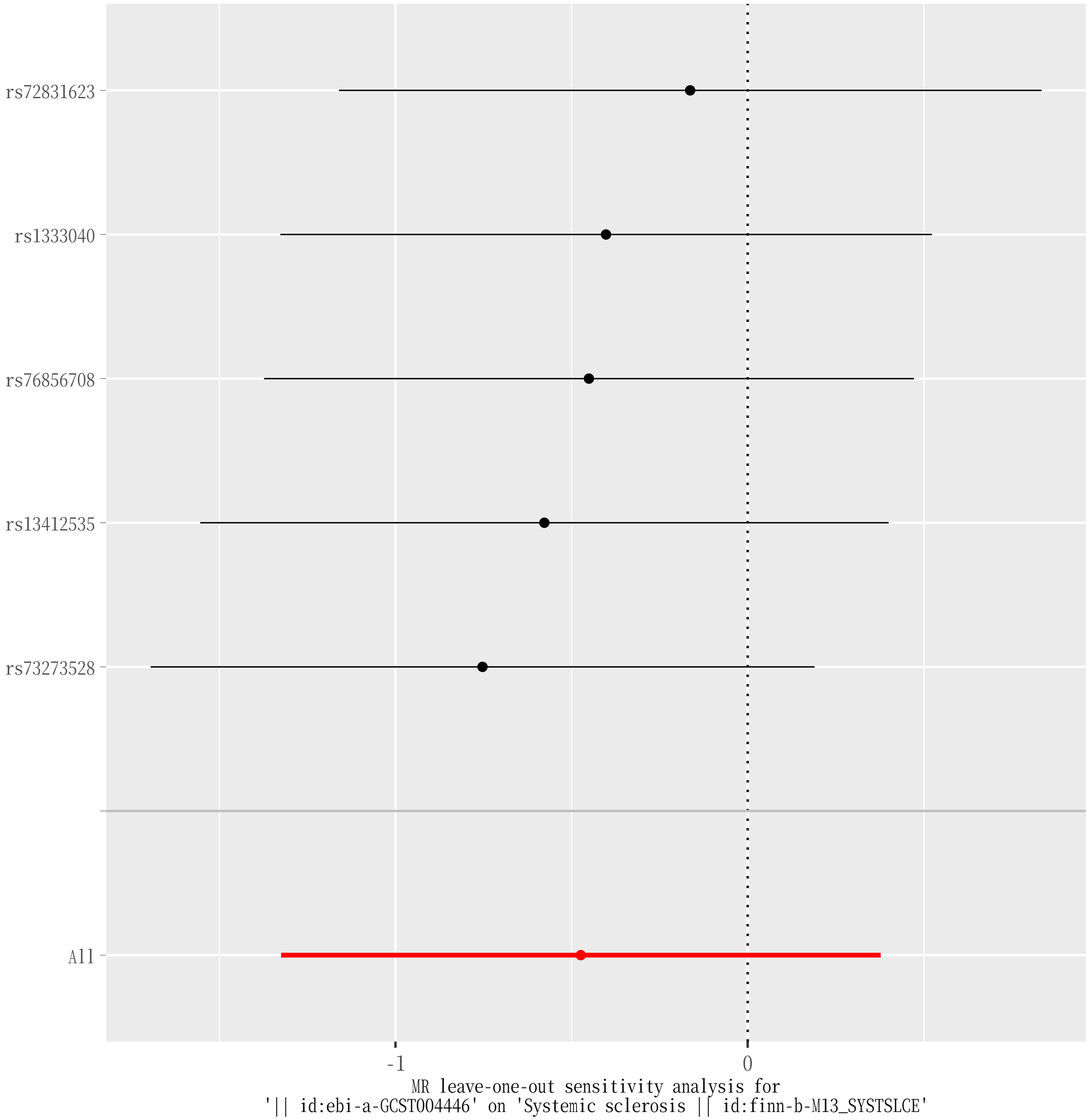

rs72831623
rs1333040
rs76856708
rs13412535
rs73273528
All
-1
0
MR leave-one-out sensitivity analysis for
'|| id:ebi-a-GCST004446' on 'Systemic sclerosis || id:finn-b-M13_SYSTSLCE'

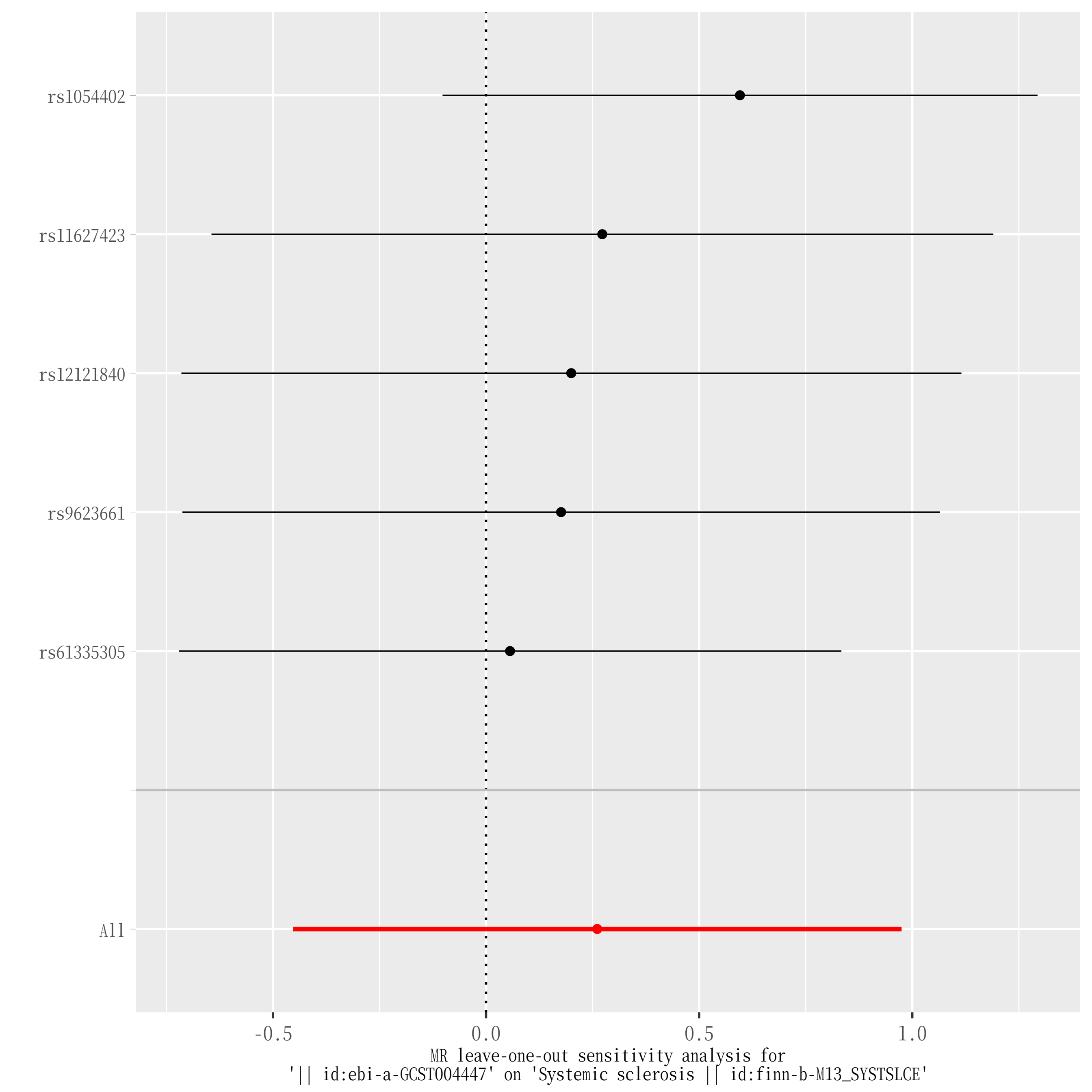
rs1054402
rs11627423
rs12121840
rs9623661
rs61335305
All
-0.5
0.0
0.5
1.0
MR leave-one-out sensitivity analysis for
'|| id:ebi-a-GCST004447' on 'Systemic sclerosis || id:finn-b-M13_SYSTSLCE'

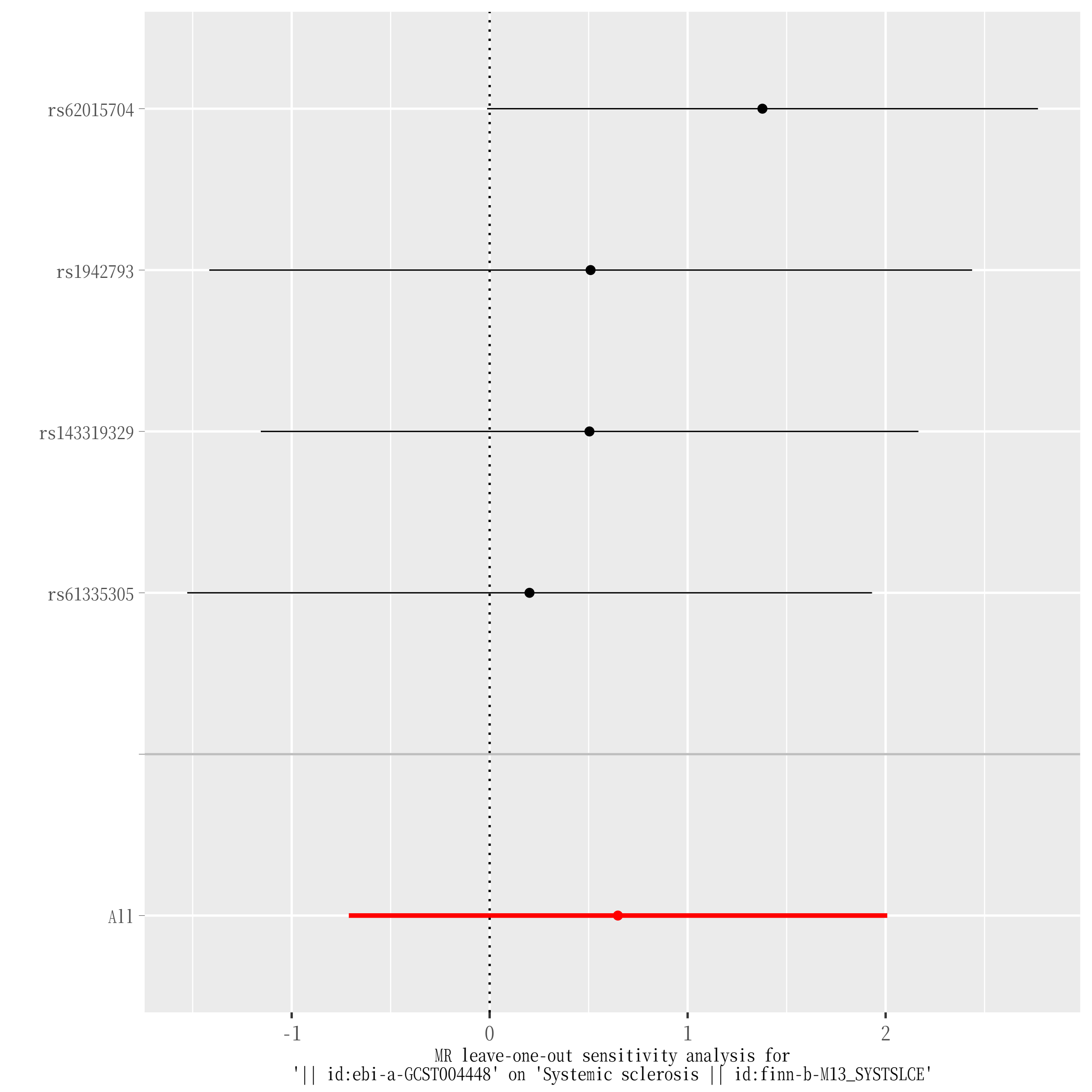
rs62015704
rs1942793
rs143319329
rs61335305
All
-1
0
1
2
MR leave-one-out sensitivity analysis for
'|| id:ebi-a-GCST004448' on 'Systemic sclerosis || id:finn-b-M13_SYSTSLCE'

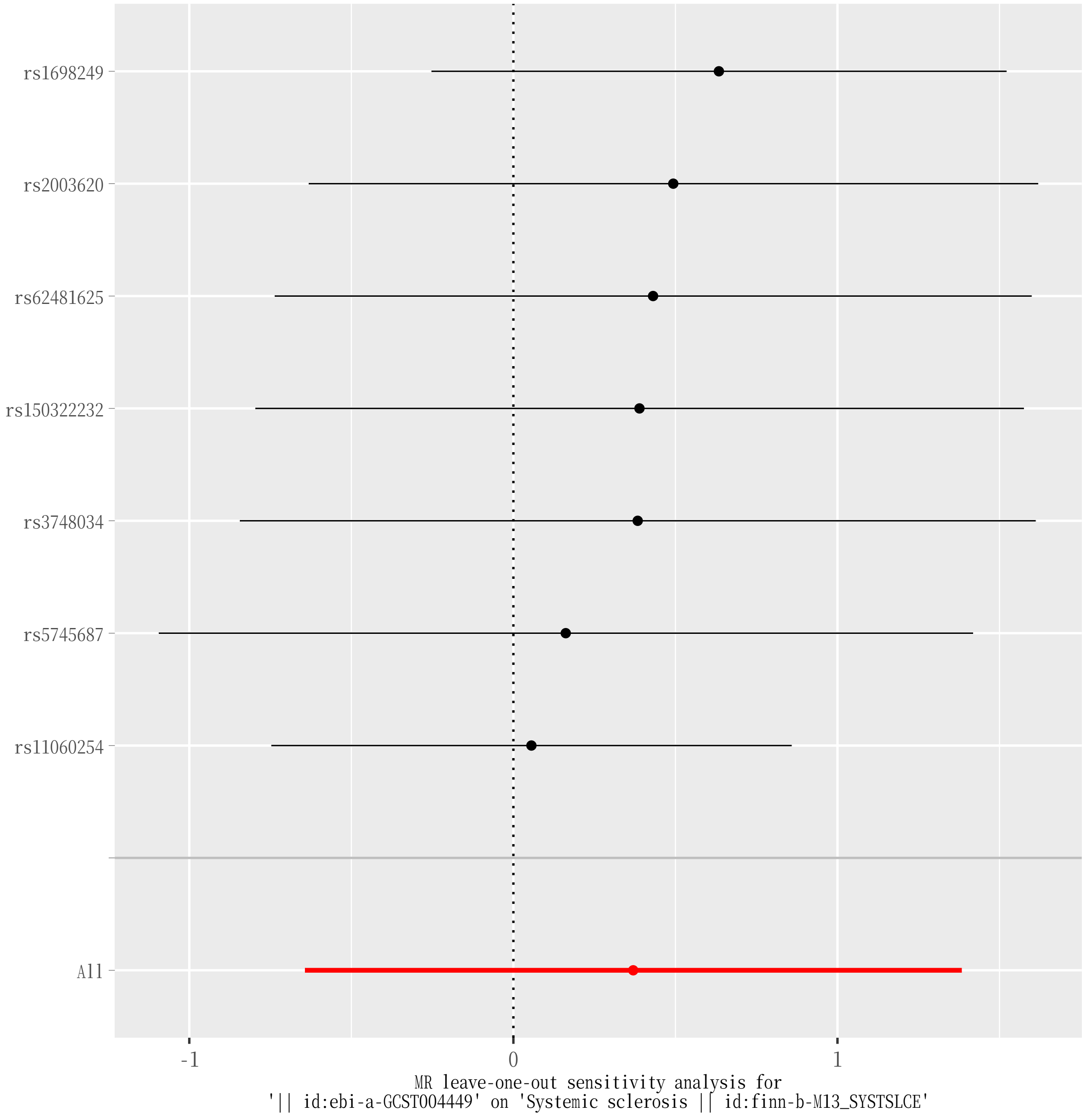
rs1698249
rs2003620
rs62481625
rs150322232
rs3748034
rs5745687
rs11060254
All
-1
0
1
MR leave-one-out sensitivity analysis for
'|| id:ebi-a-GCST004449' on 'Systemic sclerosis || id:finn-b-M13_SYSTSLCE'

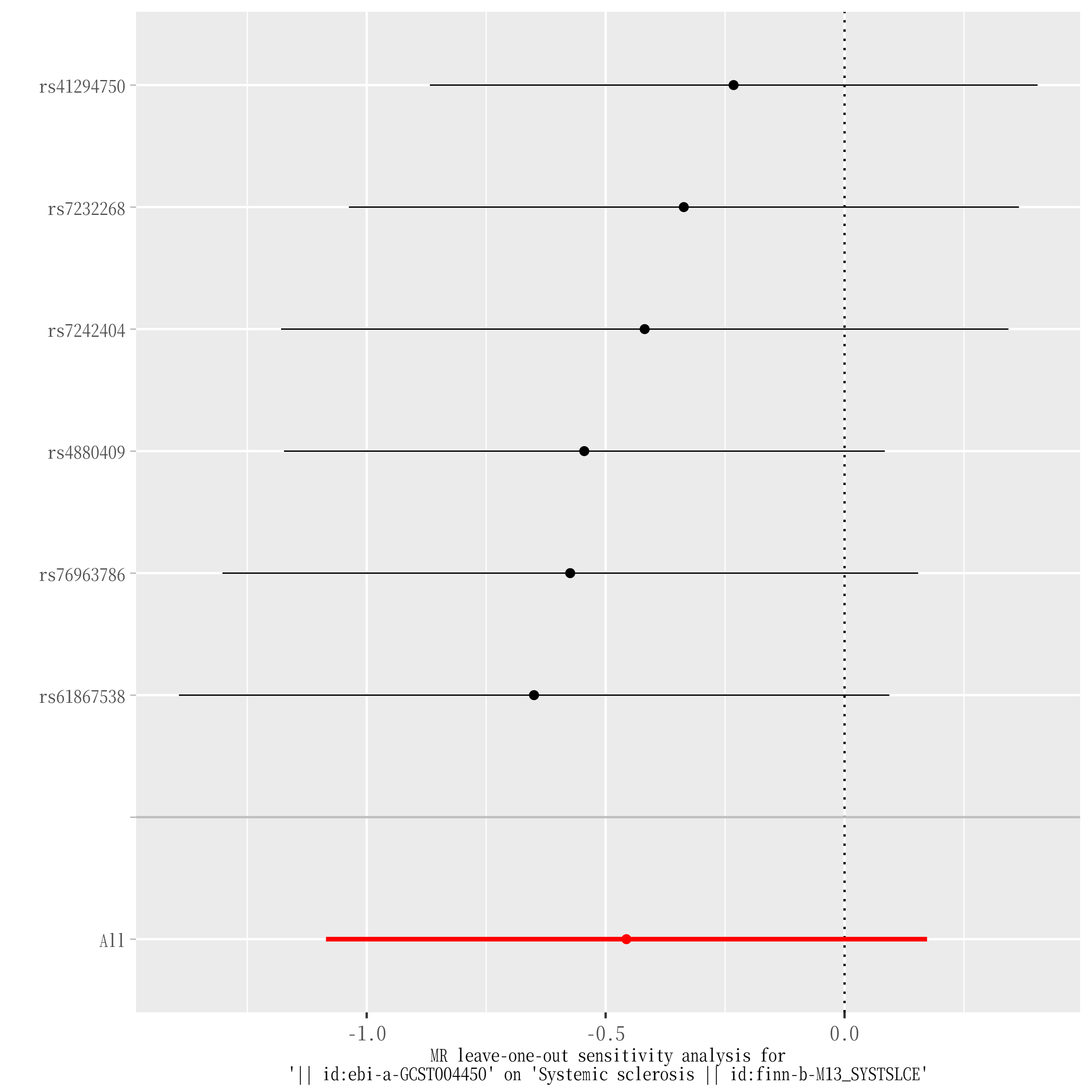
rs41294750
rs7232268
rs7242404
rs4880409
rs76963786
rs61867538
All
-1.0
-0.5
0.0
MR leave-one-out sensitivity analysis for
'|| id:ebi-a-GCST004450' on 'Systemic sclerosis || id:finn-b-M13_SYSTSLCE'

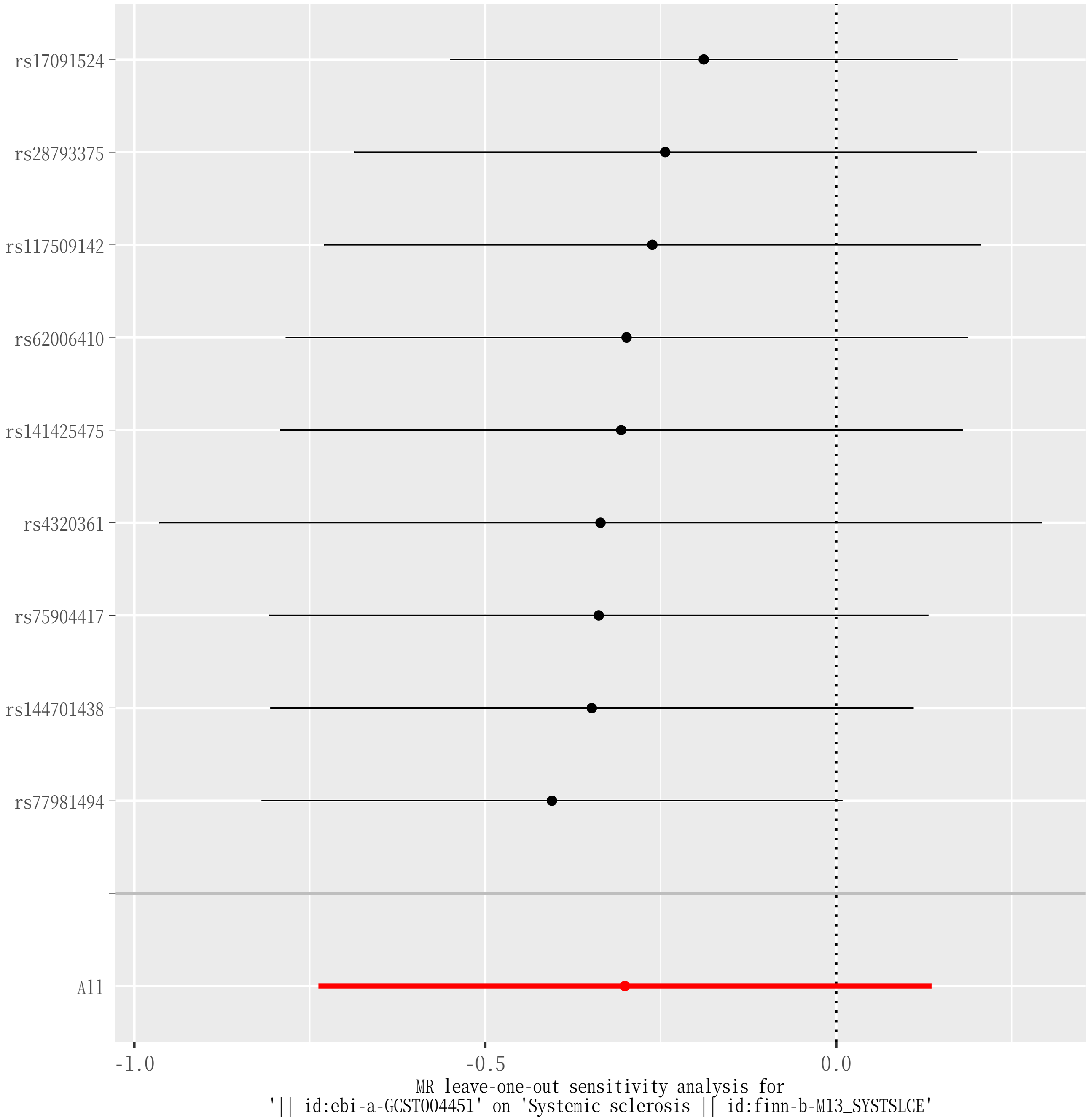
rs17091524
rs28793375
rs117509142
rs62006410
rs141425475
rs4320361
rs75904417
rs144701438
rs77981494
All
-1.0
-0.5
0.0
MR leave-one-out sensitivity analysis for
'|| id:ebi-a-GCST004451' on 'Systemic sclerosis || id:finn-b-M13_SYSTSLCE'

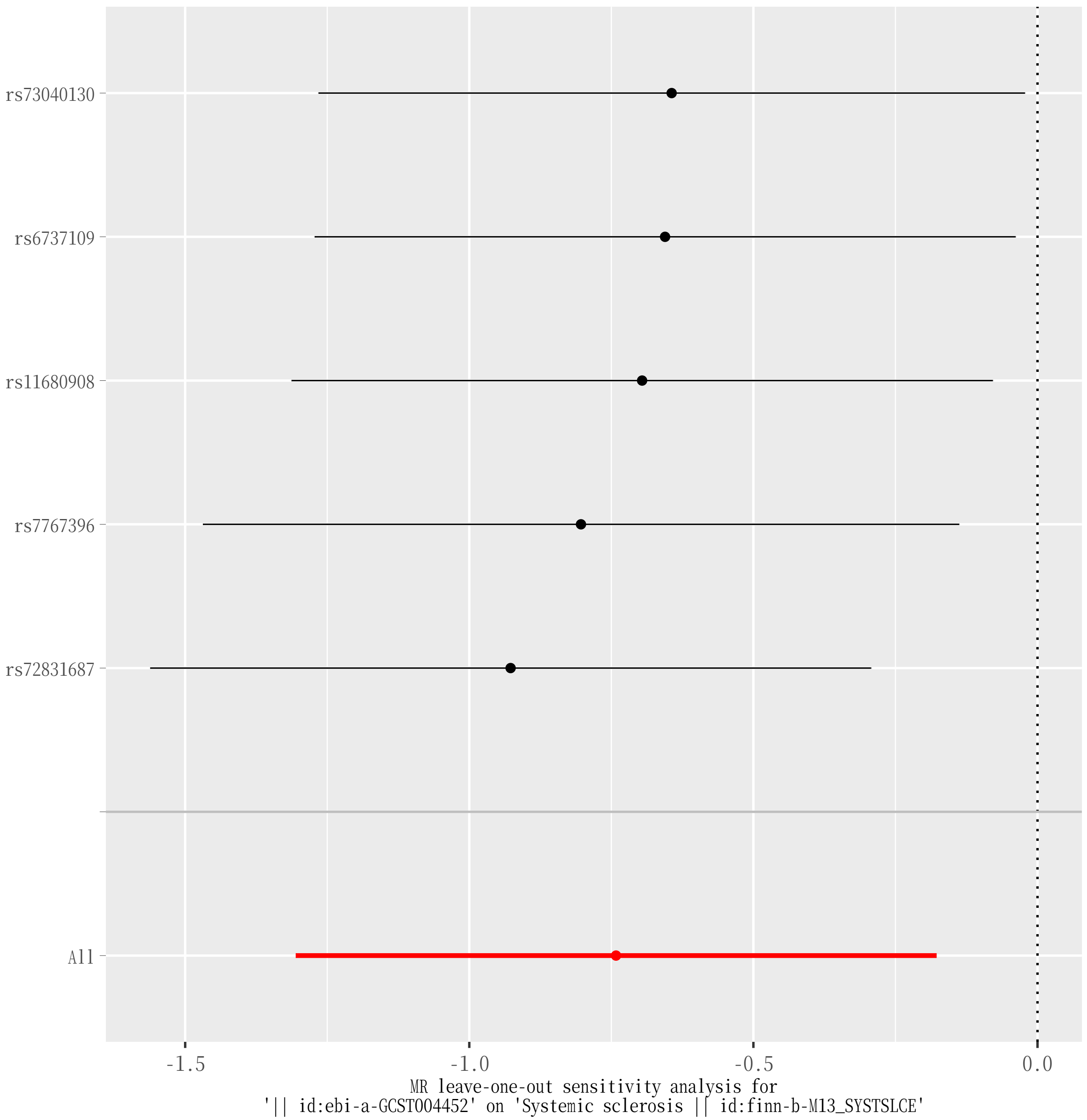
rs73040130
rs6737109
rs11680908
rs7767396
rs72831687
All
-1.5
-1.0
-0.5
0.0
MR leave-one-out sensitivity analysis for
'|| id:ebi-a-GCST004452' on 'Systemic sclerosis || id:finn-b-M13_SYSTSLCE'

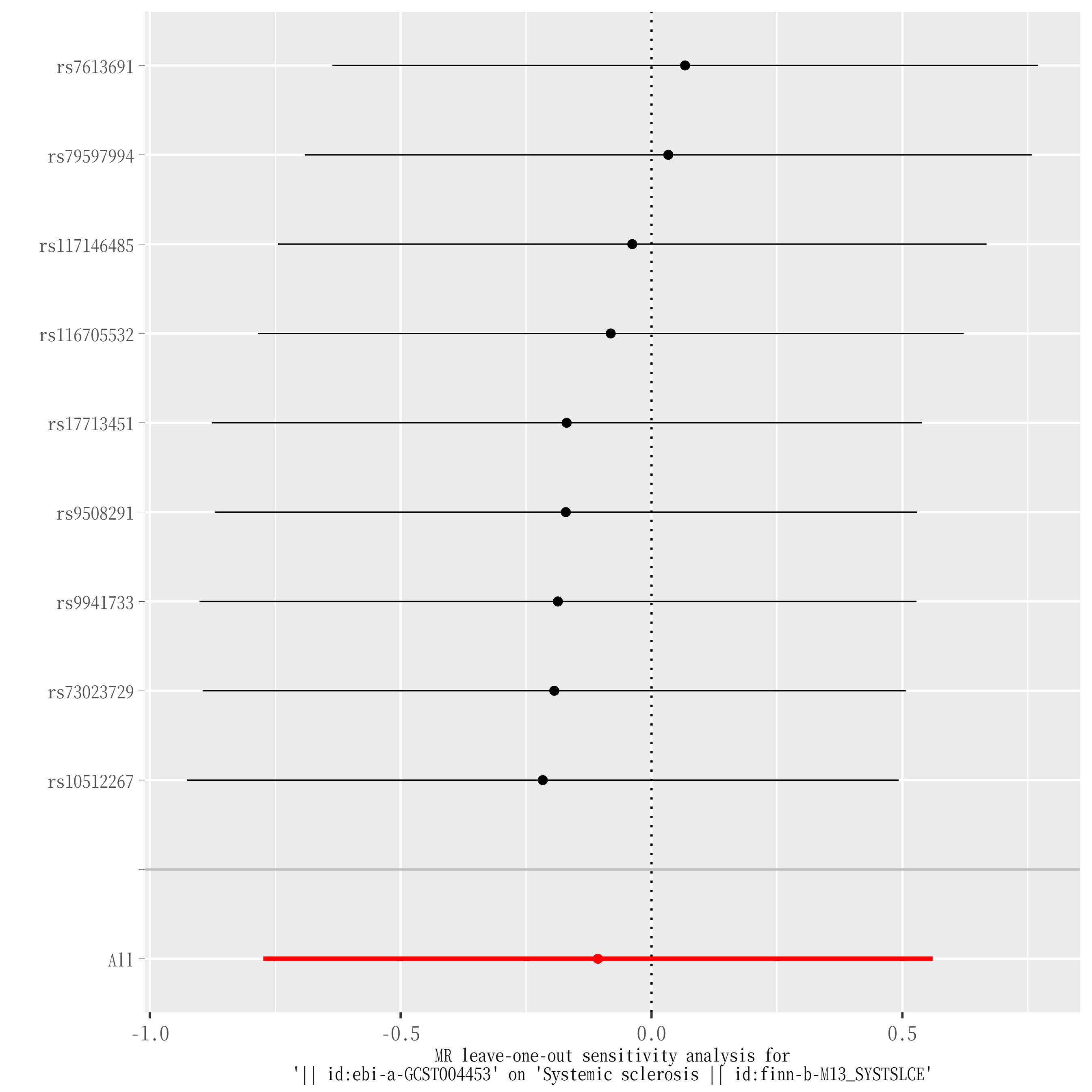
rs7613691
rs79597994
rs117146485
rs116705532
rs17713451
rs9508291
rs9941733
rs73023729
rs10512267
All
-1.0
-0.5
0.0
0.5
MR leave-one-out sensitivity analysis for
'|| id:ebi-a-GCST004453' on 'Systemic sclerosis || id:finn-b-M13_SYSTSLCE'

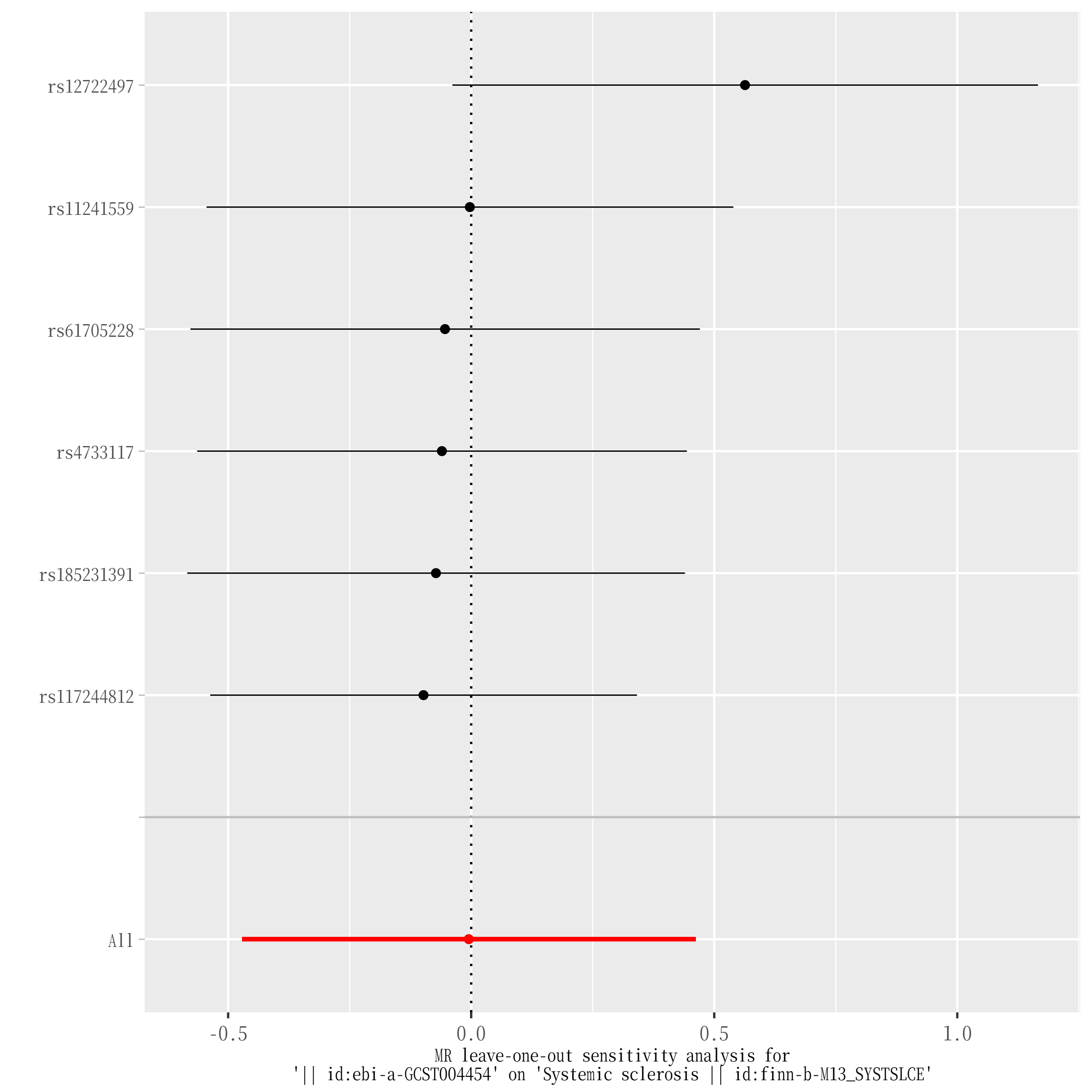
rs12722497
rs11241559
rs61705228
rs4733117
rs185231391
rs117244812
All
-0.5
0.0
0.5
1.0
MR leave-one-out sensitivity analysis for
'|| id:ebi-a-GCST004454' on 'Systemic sclerosis || id:finn-b-M13_SYSTSLCE'

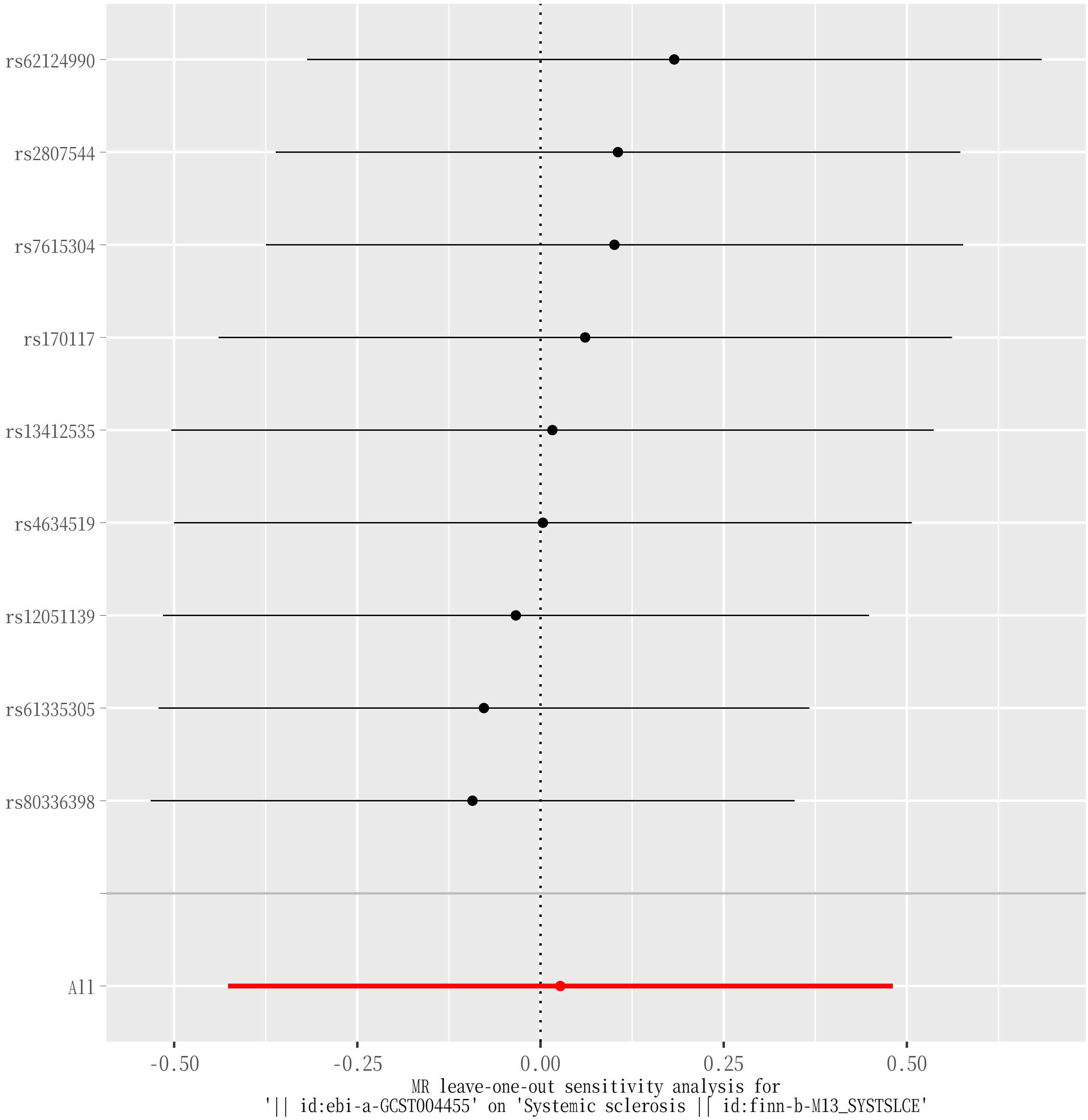
rs62124990
rs2807544
rs7615304
rs170117
rs13412535
rs4634519
rs12051139
rs61335305
rs80336398
All
-0.50
-0.25
0.00
0.25
0.50
MR leave-one-out sensitivity analysis for
'|| id:ebi-a-GCST004455' on 'Systemic sclerosis || id:finn-b-M13_SYSTSLCE'

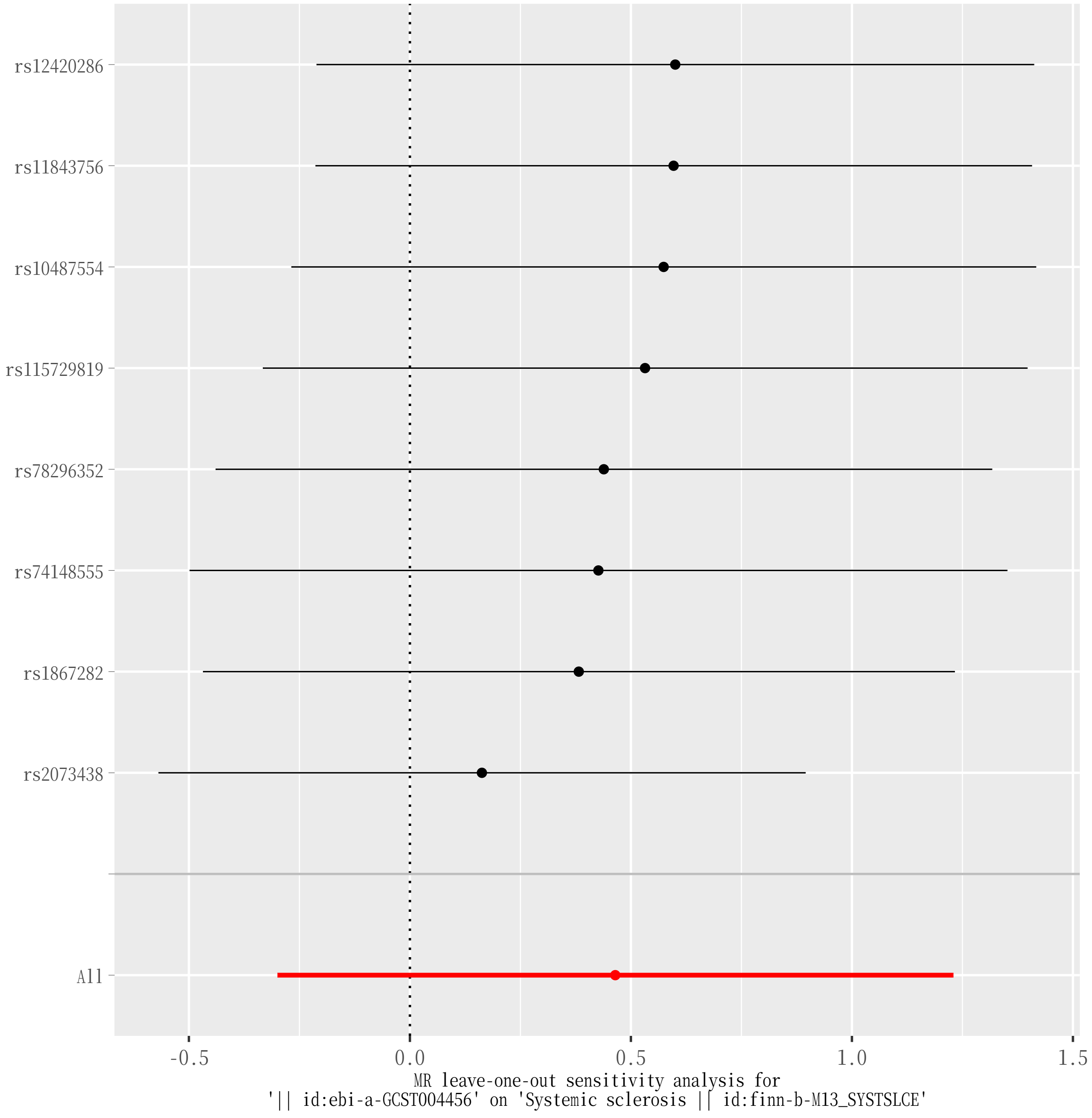
rs12420286
rs11843756
rs10487554
rs115729819
rs78296352
rs74148555
rs1867282
rs2073438
All
-0.5
0.0
0.5
1.0
1.5
MR leave-one-out sensitivity analysis for
'|| id:ebi-a-GCST004456' on 'Systemic sclerosis || id:finn-b-M13_SYSTSLCE'

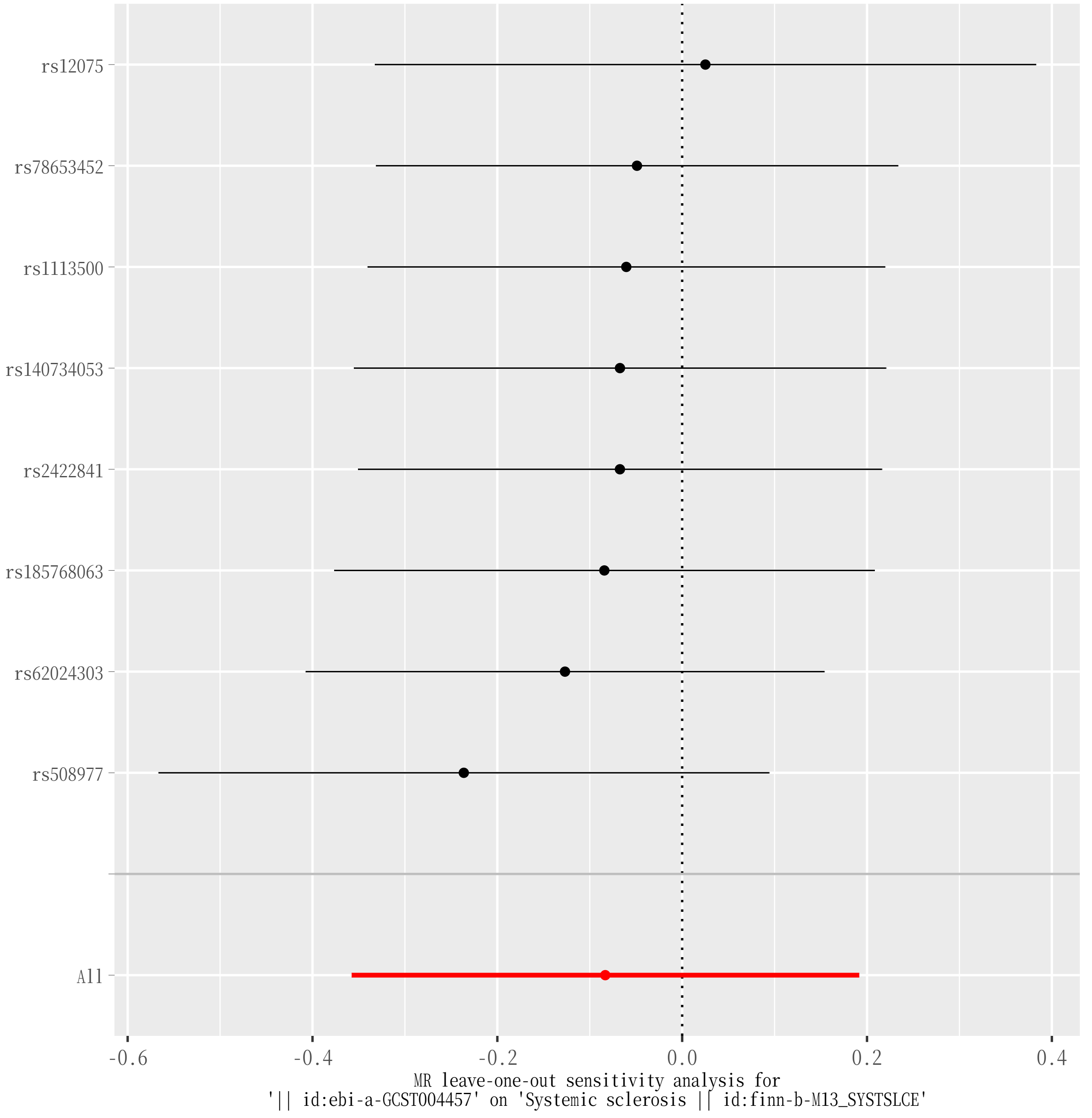
rs12075
rs78653452
rs1113500
rs140734053
rs2422841
rs185768063
rs62024303
rs508977
All
-0.6
-0.4
-0.2
0.0
0.2
0.4
MR leave-one-out sensitivity analysis for
'|| id:ebi-a-GCST004457' on 'Systemic sclerosis || id:finn-b-M13_SYSTSLCE'

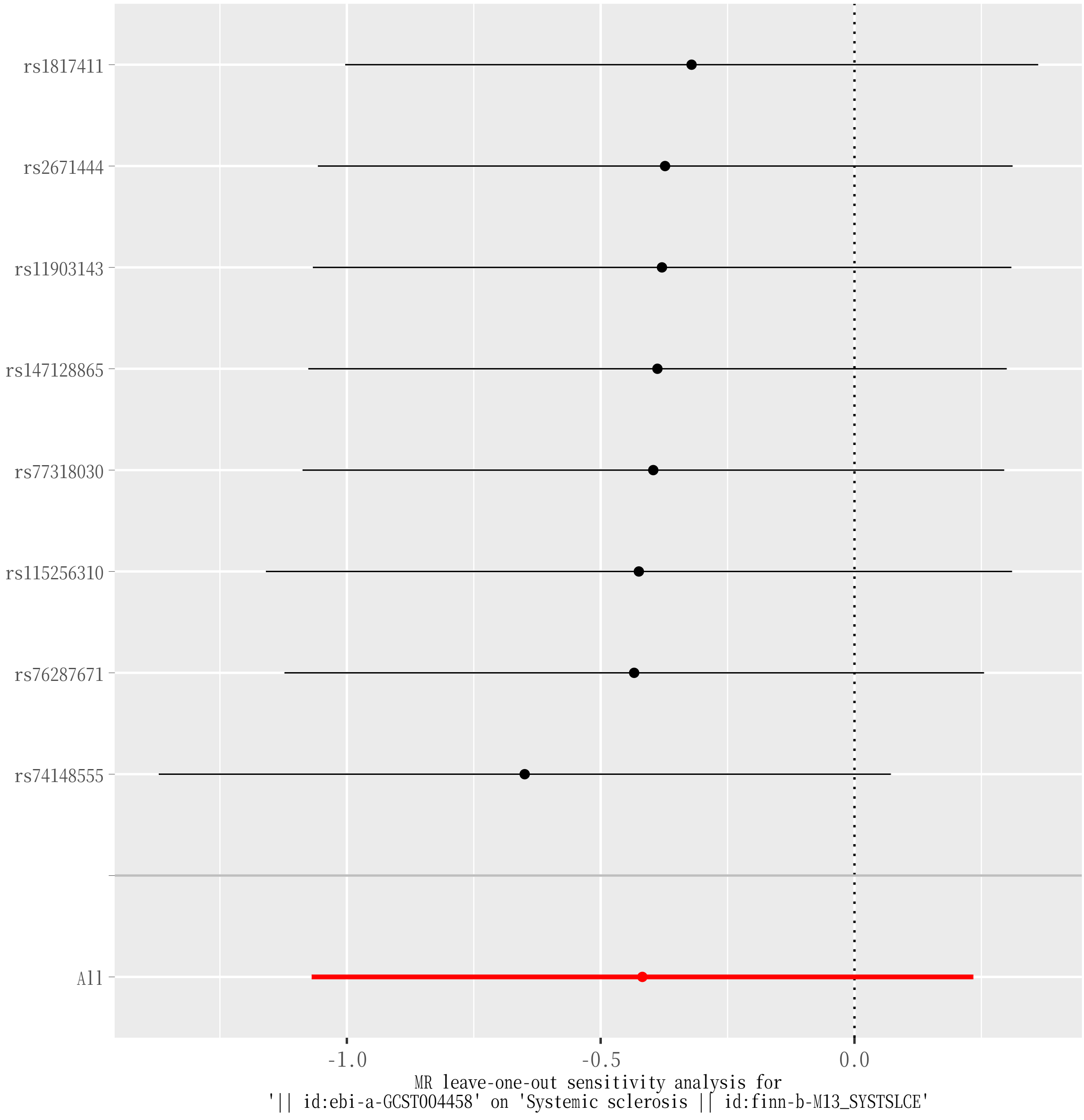

rs1817411
rs2671444
rs11903143
rs147128865
rs77318030
rs115256310
rs76287671
rs74148555
All
-1.0
-0.5
0.0
MR leave-one-out sensitivity analysis for
'|| id:ebi-a-GCST004458' on 'Systemic sclerosis || id:finn-b-M13_SYSTSLCE'

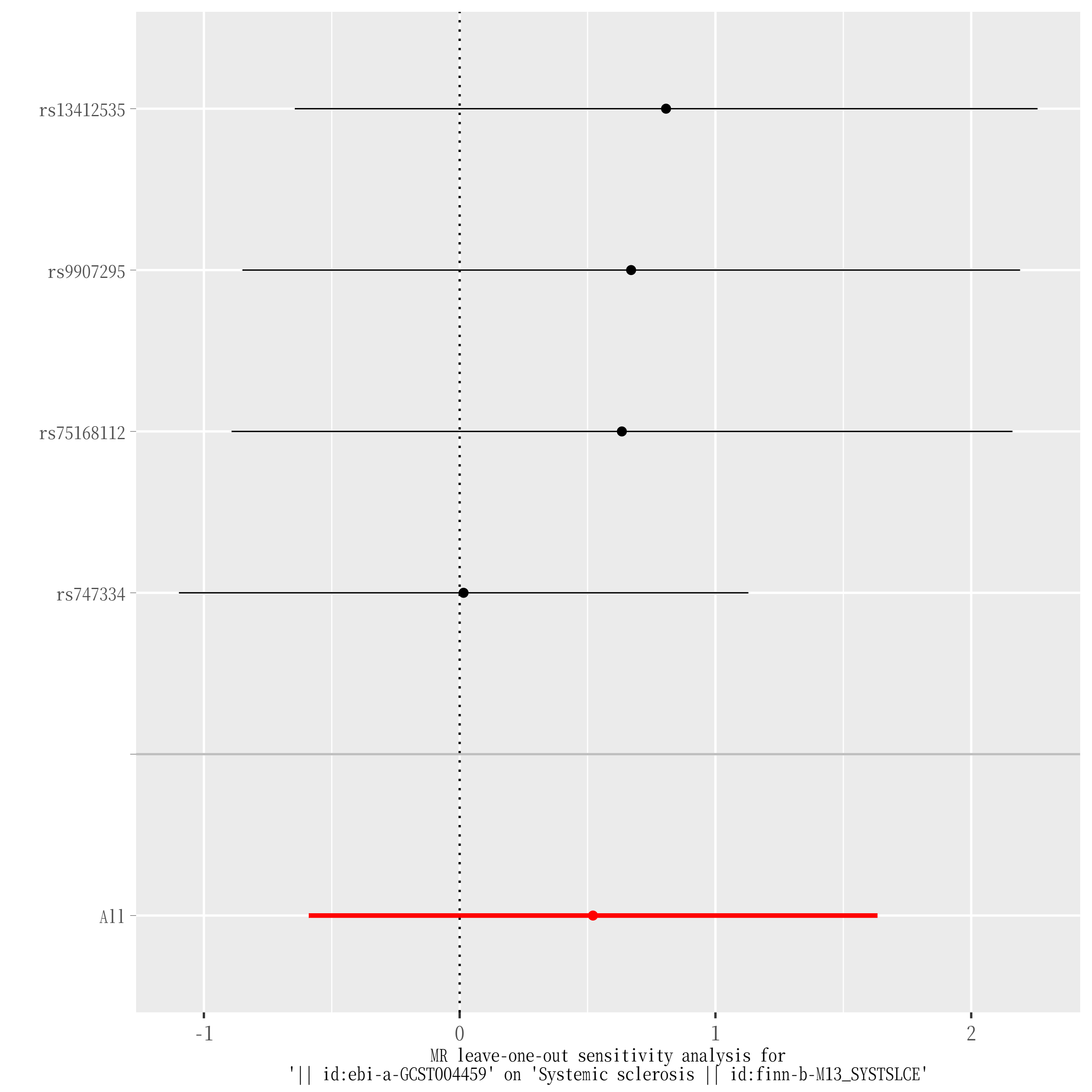
rs13412535
rs9907295
rs75168112
rs747334
All
-1
0
1
2
MR leave-one-out sensitivity analysis for
'|| id:ebi-a-GCST004459' on 'Systemic sclerosis || id:finn-b-M13_SYSTSLCE'

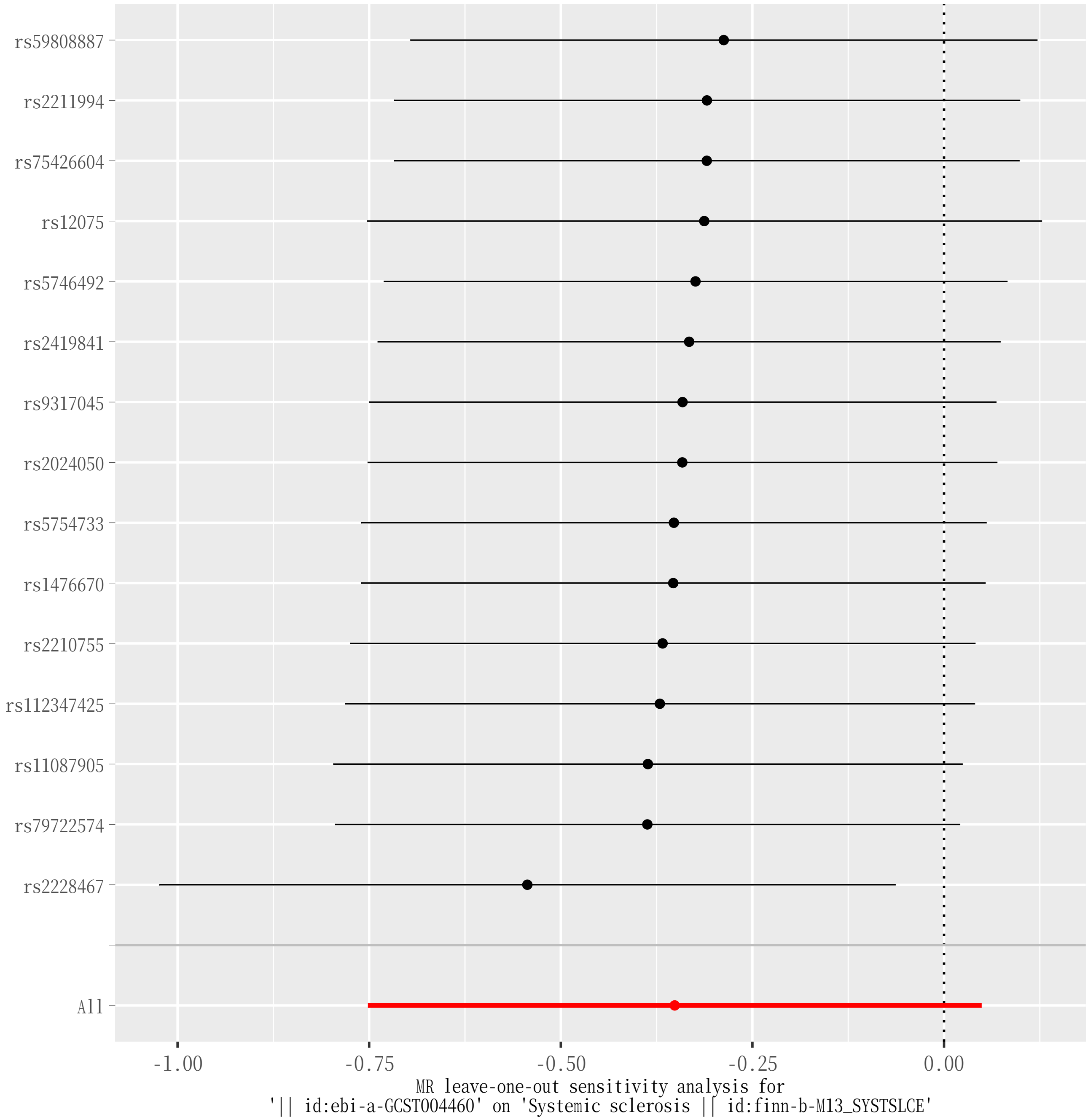
rs59808887
rs2211994
rs75426604
rs12075
rs5746492
rs2419841
rs9317045
rs2024050
rs5754733
rs1476670
rs2210755
rs112347425
rs11087905
rs79722574
rs2228467
All
-1.00
-0.75
-0.50
-0.25
0.00
MR leave-one-out sensitivity analysis for
'|| id:ebi-a-GCST004460' on 'Systemic sclerosis || id:finn-b-M13_SYSTSLCE'
