# Supplementary material for: Associations of the circulating levels of cytokines with risk of systemic sclerosis: a bidirectional Mendelian randomized study
Source: Front Immunol. 2024 Feb 28;15:1330560. doi: 10.3389/fimmu.2024.1330560 (PMC10933062; doi:10.3389/fimmu.2024.1330560)
Supplement: Supplementary file 3 [file DataSheet_2.pdf]

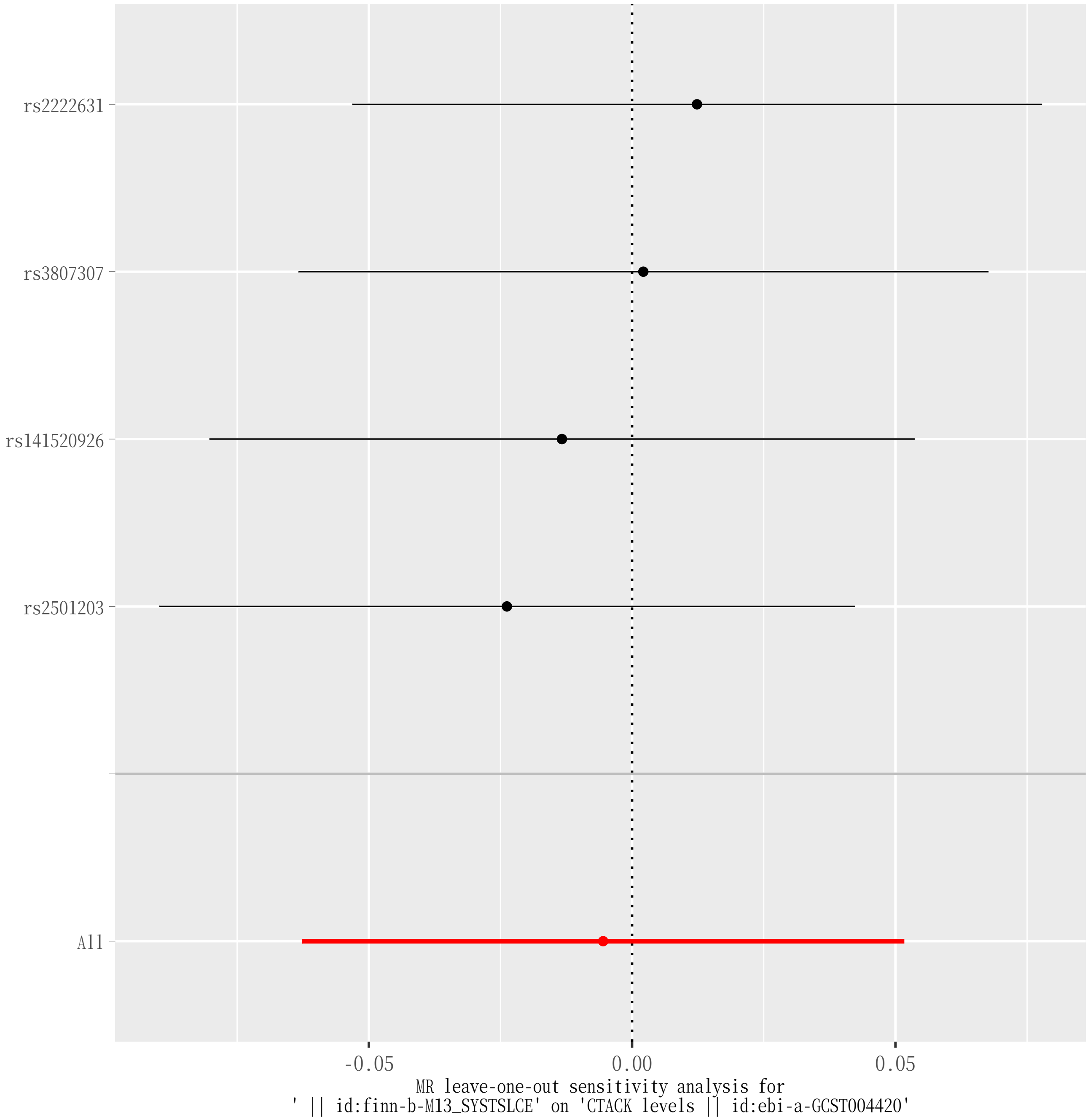
rs2222631
rs3807307
rs141520926
rs2501203
All
-0.05
0.00
0.05
MR leave-one-out sensitivity analysis for
' || id:finn-b-M13_SYSTSLCE' on 'CTACK levels || id:ebi-a-GCST004420'

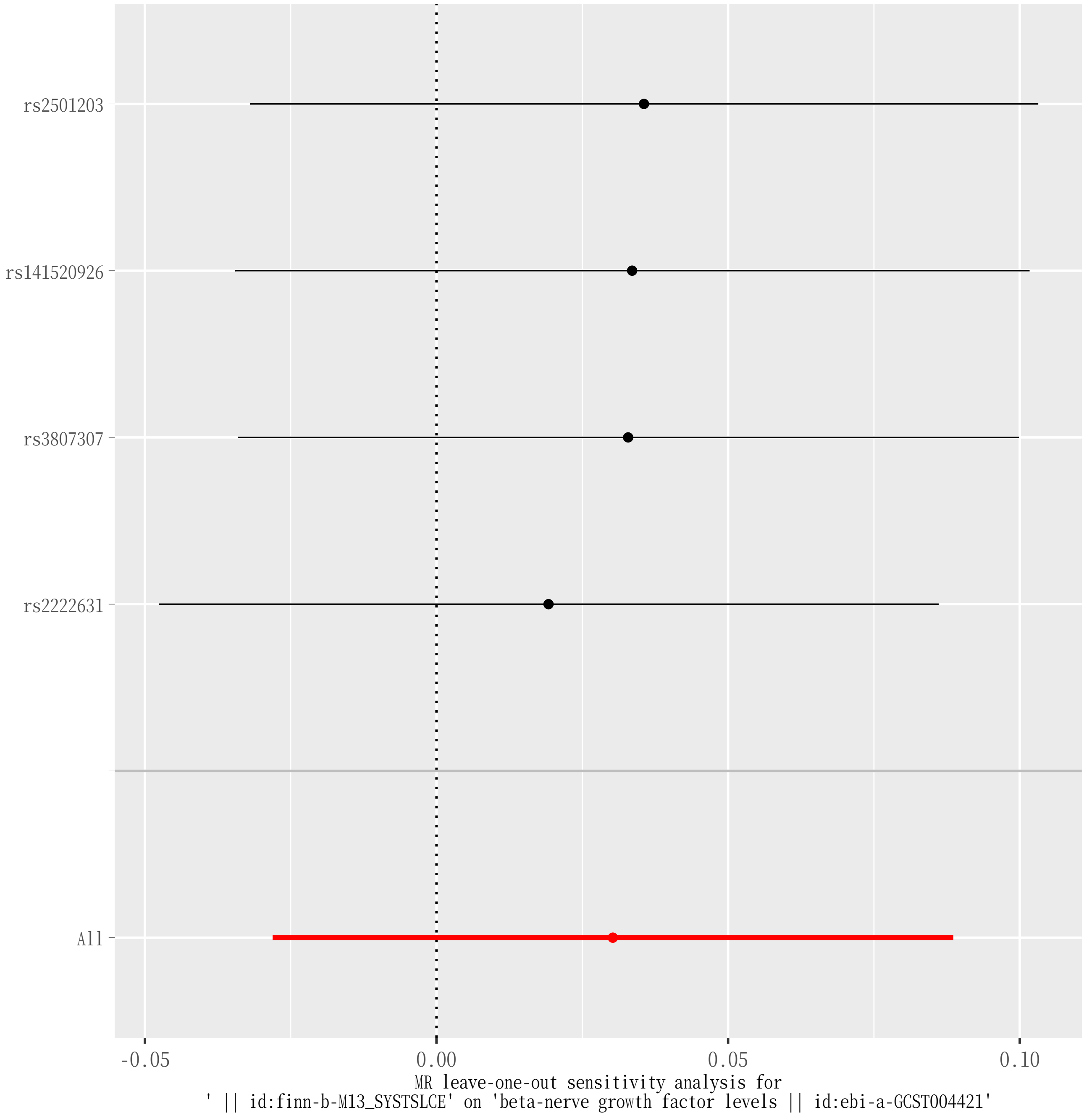
rs2501203
rs141520926
rs3807307
rs2222631
All
-0.05
0.00
0.05
0.10
MR leave-one-out sensitivity analysis for
' || id:finn-b-M13_SYSTSLCE' on 'beta-nerve growth factor levels || id:ebi-a-GCST004421'

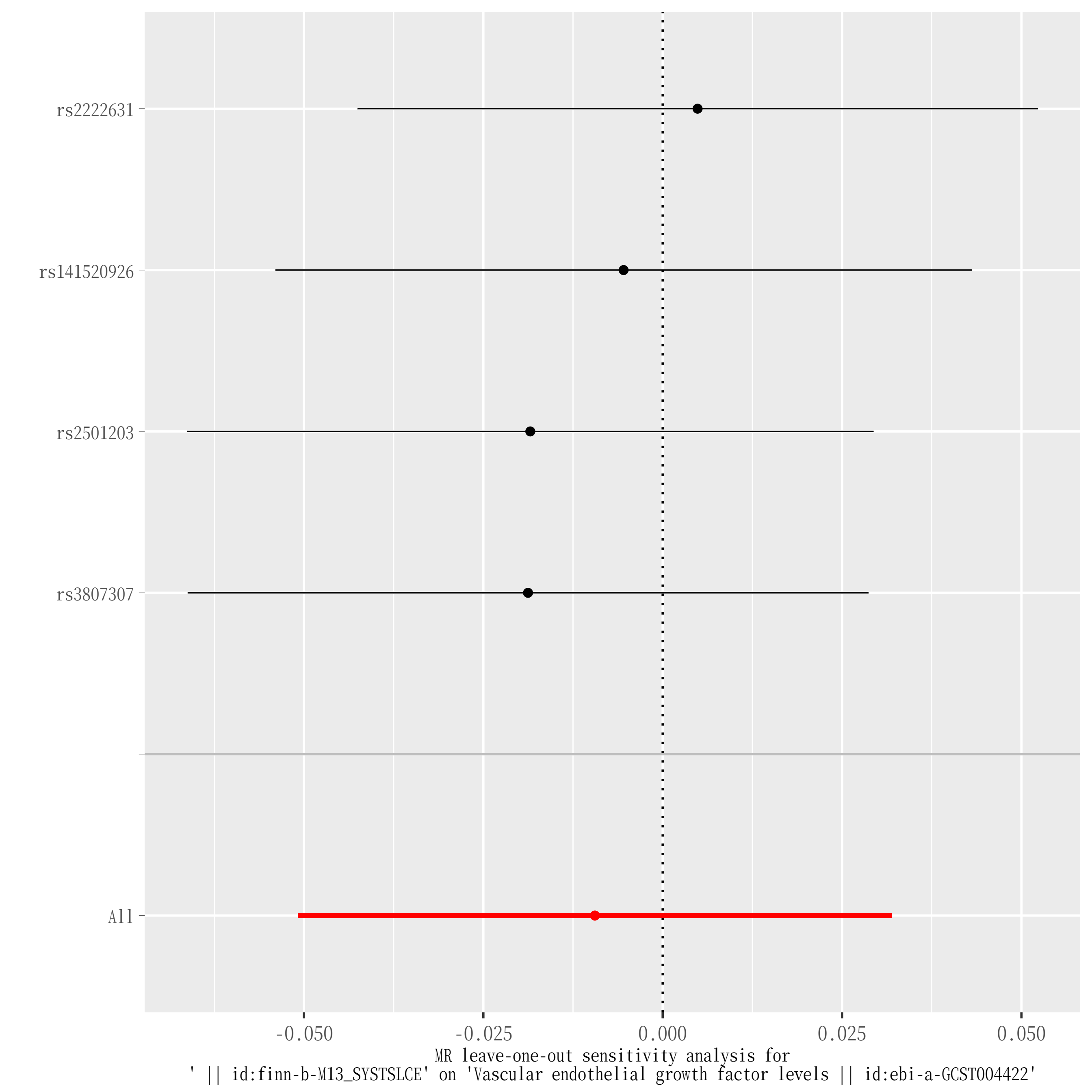
rs2222631
rs141520926
rs2501203
rs3807307
All
-0.050
-0.025
0.000
0.025
0.050
MR leave-one-out sensitivity analysis for
' || id:finn-b-M13_SYSTSLCE' on 'Vascular endothelial growth factor levels || id:ebi-a-GCST004422'

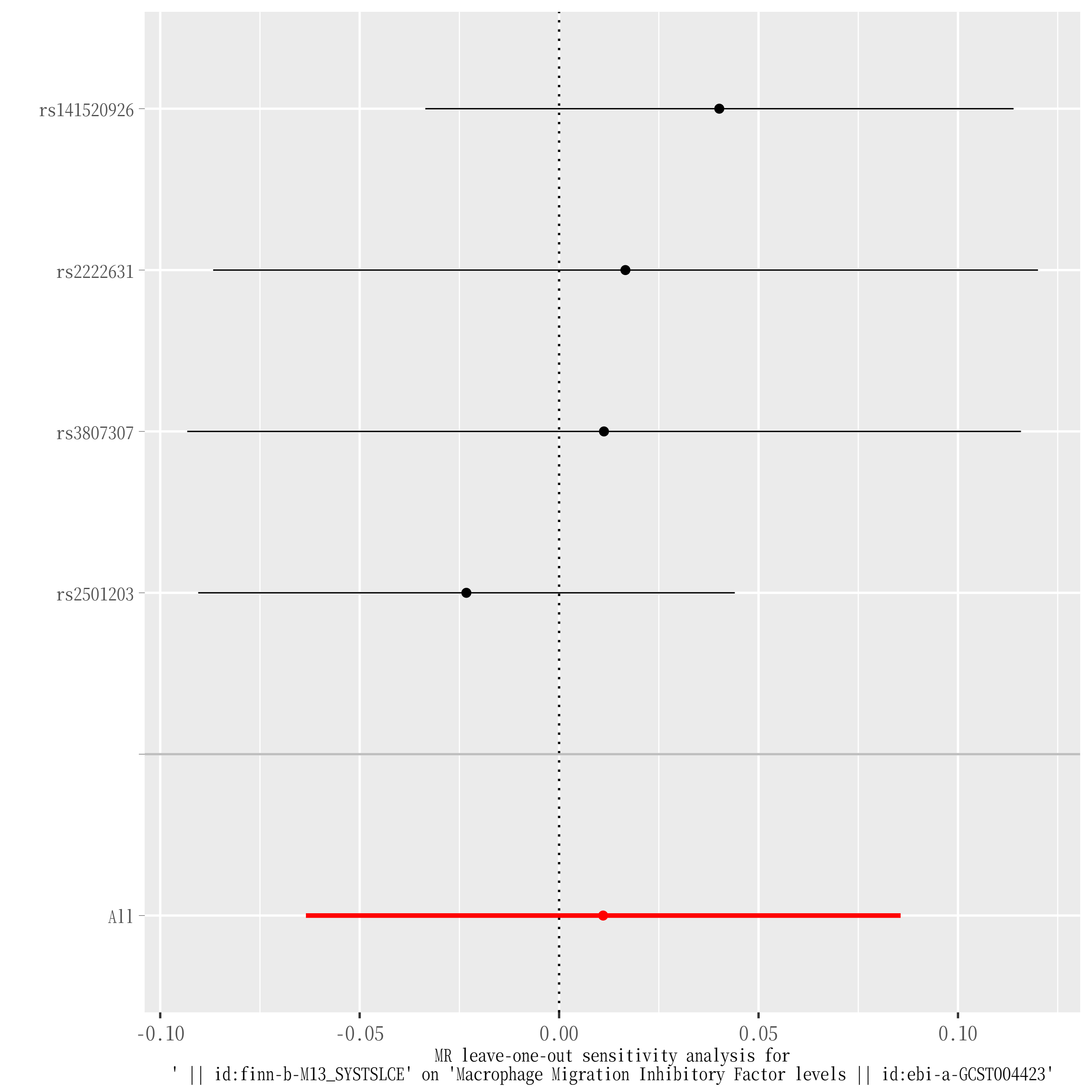

rs141520926
rs2222631
rs3807307
rs2501203
All
-0.10
-0.05
0.00
0.05
0.10
MR leave-one-out sensitivity analysis for
' || id:finn-b-M13_SYSTSLCE' on 'Macrophage Migration Inhibitory Factor levels || id:ebi-a-GCST004423'

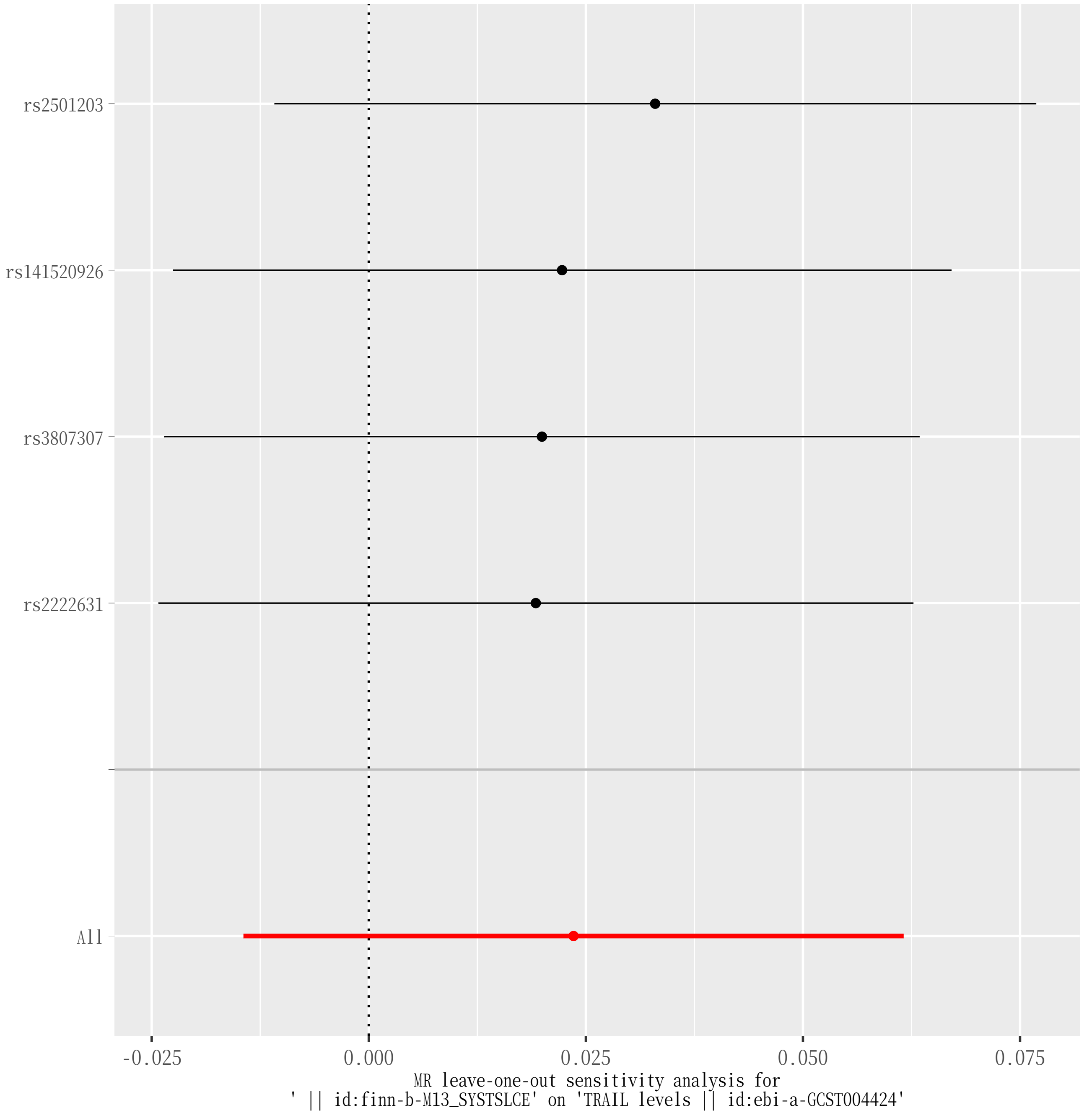

rs2501203
rs141520926
rs3807307
rs2222631
All
-0.025
0.000
0.025
0.050
0.075
MR leave-one-out sensitivity analysis for
' || id:finn-b-M13_SYSTSLCE' on 'TRAIL levels || id:ebi-a-GCST004424'

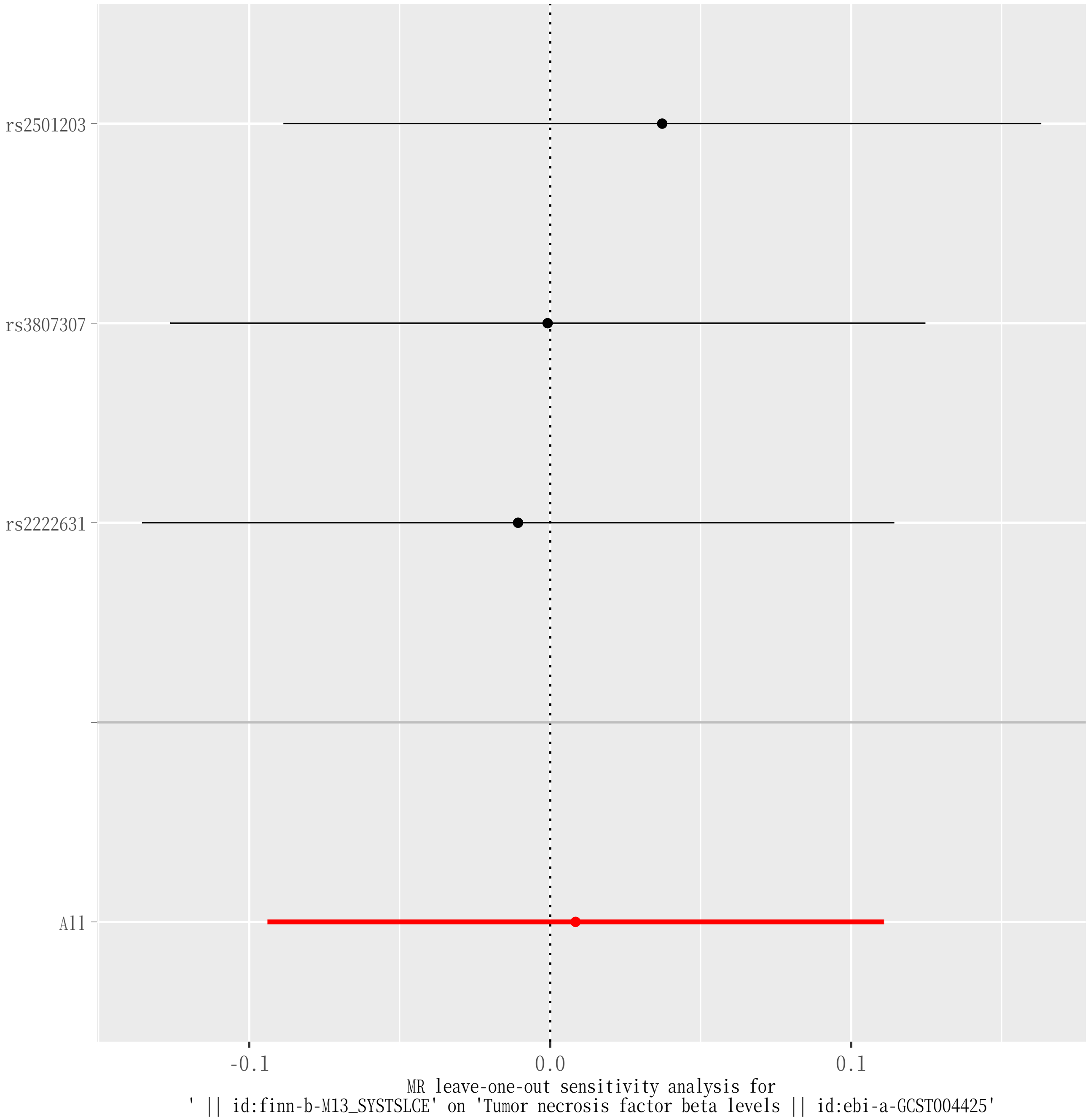

rs2501203
rs3807307
rs2222631
All
-0.1
0.0
0.1
MR leave-one-out sensitivity analysis for
' || id:finn-b-M13_SYSTSLCE' on 'Tumor necrosis factor beta levels || id:ebi-a-GCST004425'

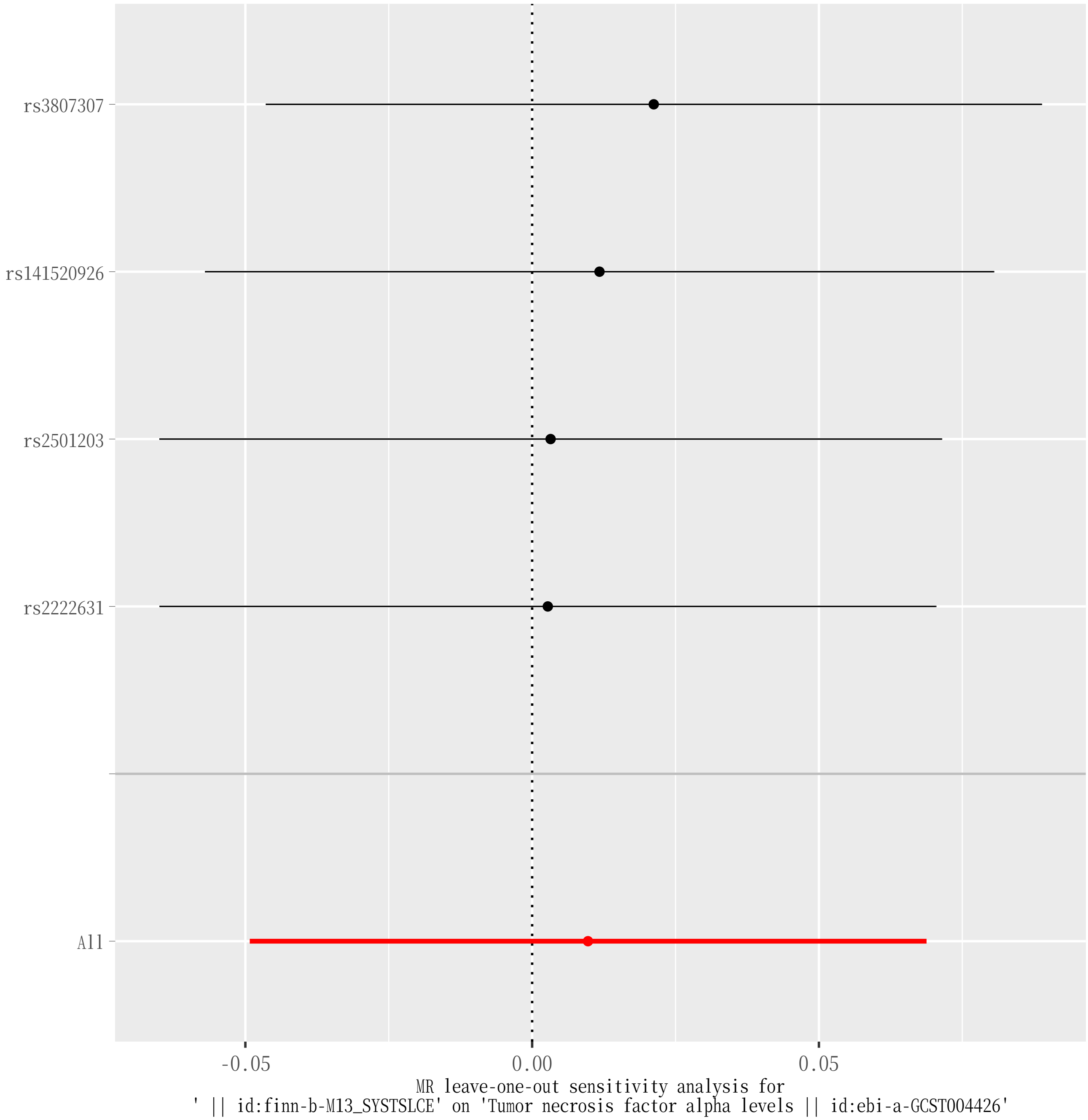

rs3807307
rs141520926
rs2501203
rs2222631
All
-0.05
0.00
0.05
MR leave-one-out sensitivity analysis for
' || id:finn-b-M13_SYSTSLCE' on 'Tumor necrosis factor alpha levels || id:ebi-a-GCST004426'

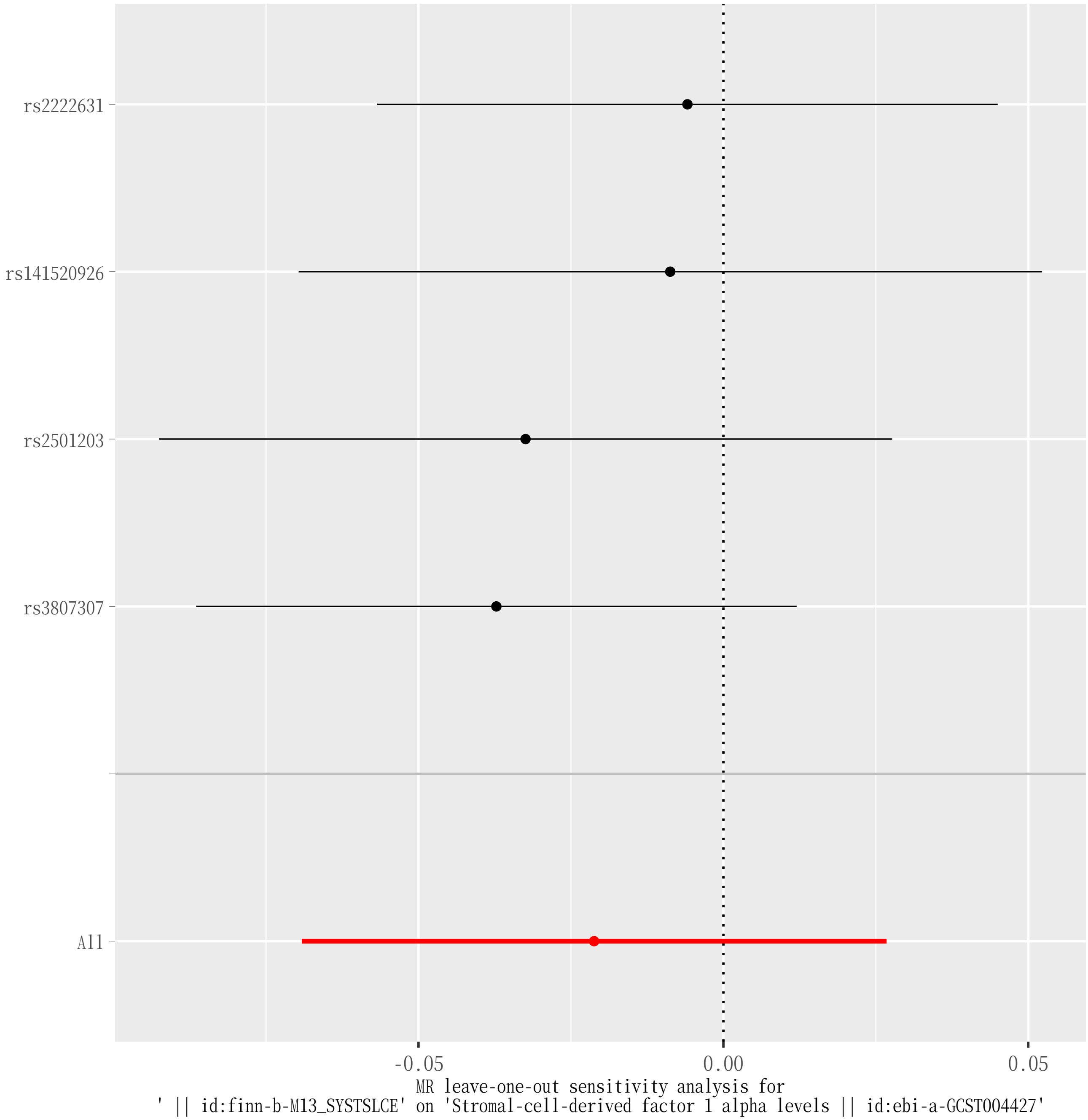

rs2222631
rs141520926
rs2501203
rs3807307
All
-0.05
0.00
0.05
MR leave-one-out sensitivity analysis for
' || id:finn-b-M13_SYSTSLCE' on 'Stromal-cell-derived factor 1 alpha levels || id:ebi-a-GCST004427'

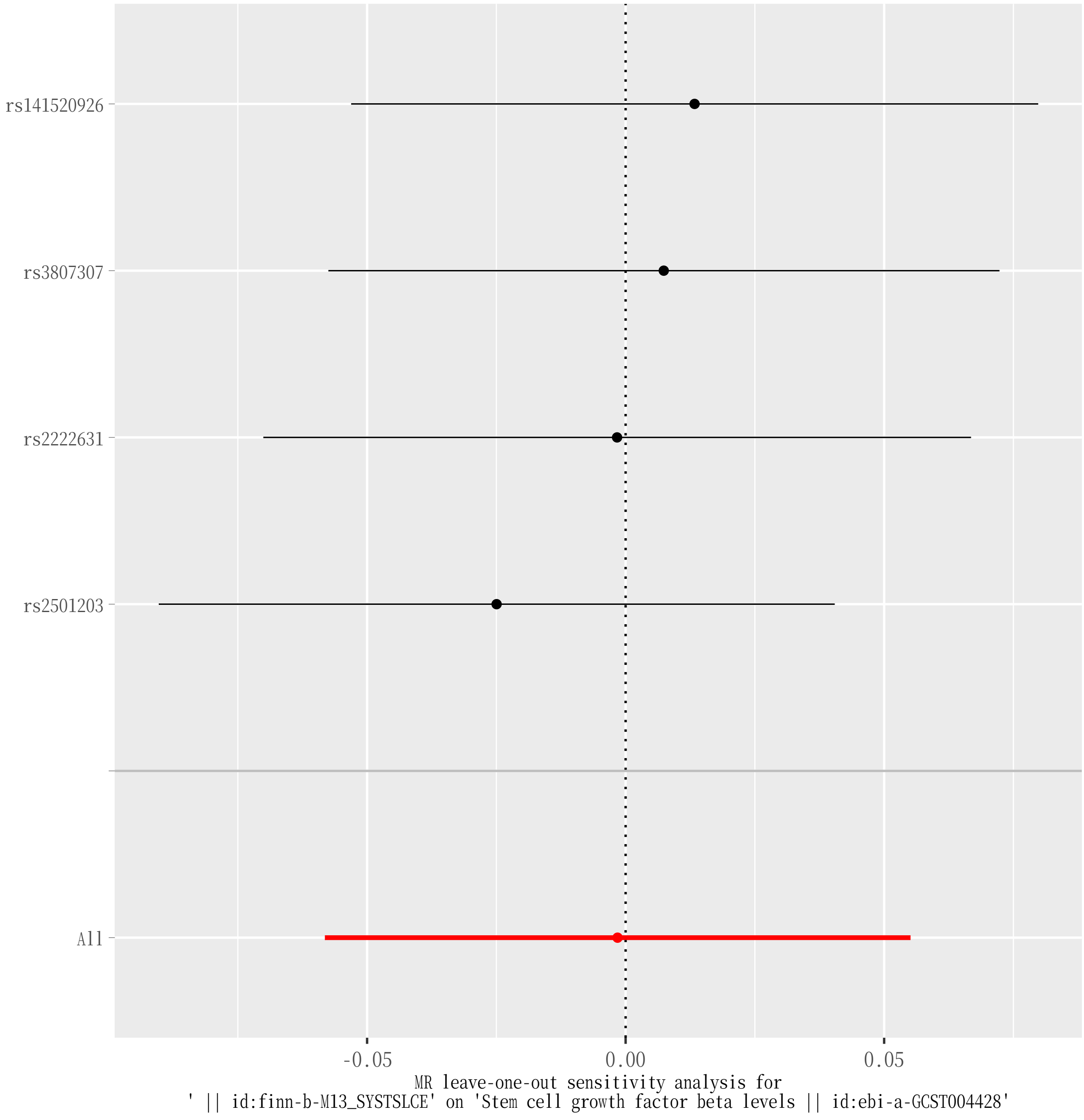
rs141520926
rs3807307
rs2222631
rs2501203
All
-0.05
0.00
0.05
MR leave-one-out sensitivity analysis for
' || id:finn-b-M13_SYSTSLCE' on 'Stem cell growth factor beta levels || id:ebi-a-GCST004428'

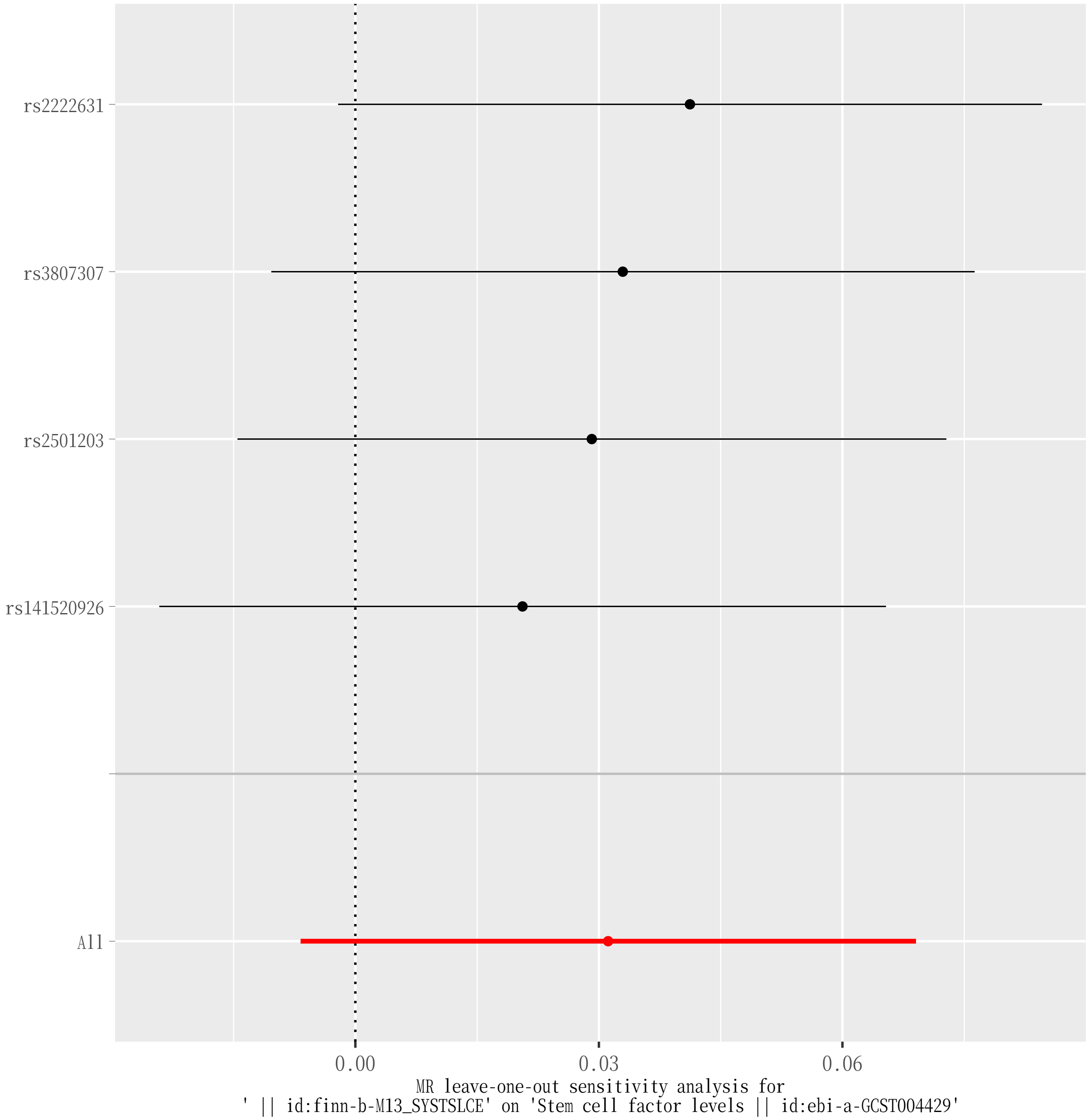

rs2222631
rs3807307
rs2501203
rs141520926
All
0.00
0.03
0.06
MR leave-one-out sensitivity analysis for
' || id:finn-b-M13_SYSTSLCE' on 'Stem cell factor levels || id:ebi-a-GCST004429'

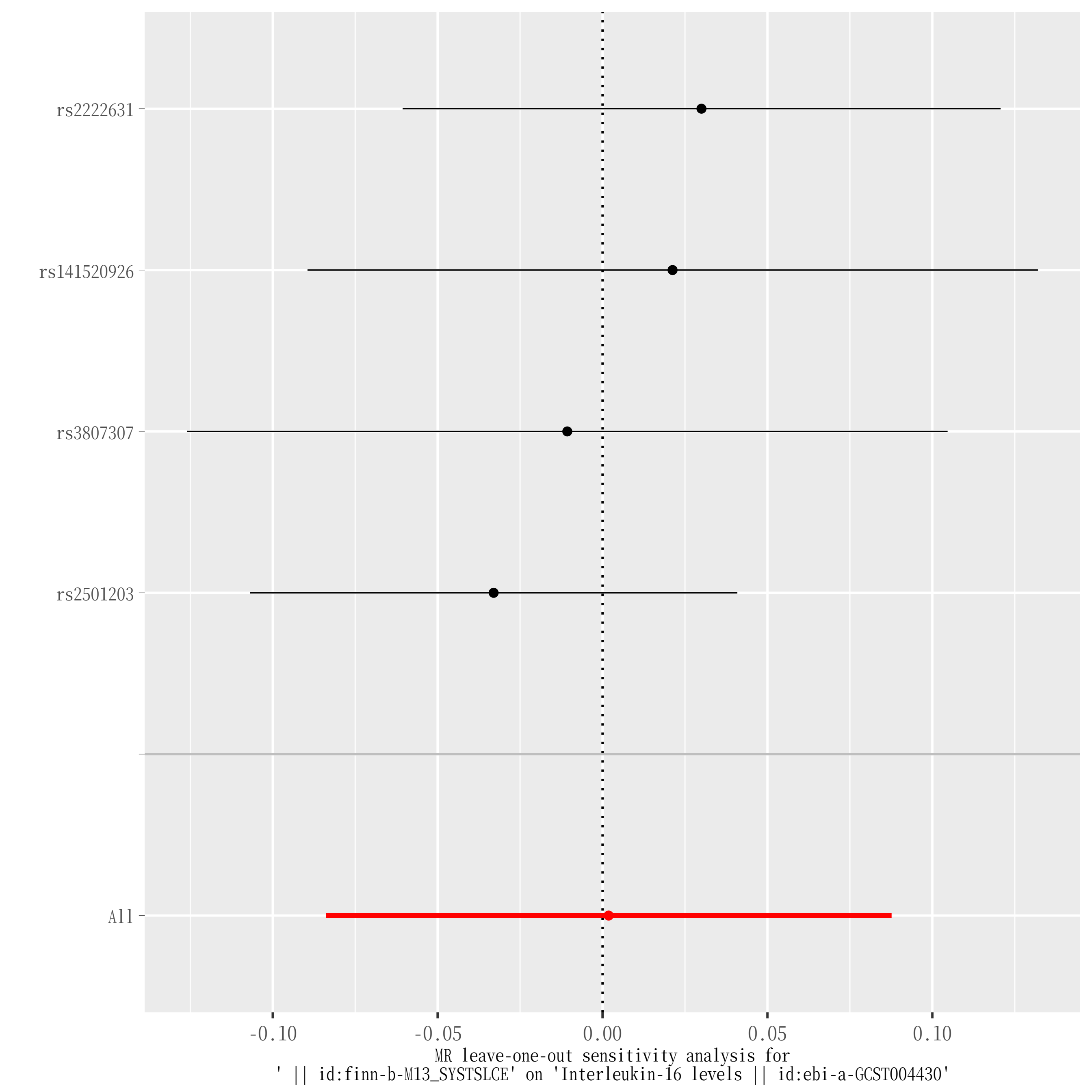
rs2222631
rs141520926
rs3807307
rs2501203
All
-0.10
-0.05
0.00
0.05
0.10
MR leave-one-out sensitivity analysis for
' || id:finn-b-M13_SYSTSLCE' on 'Interleukin-16 levels || id:ebi-a-GCST004430'

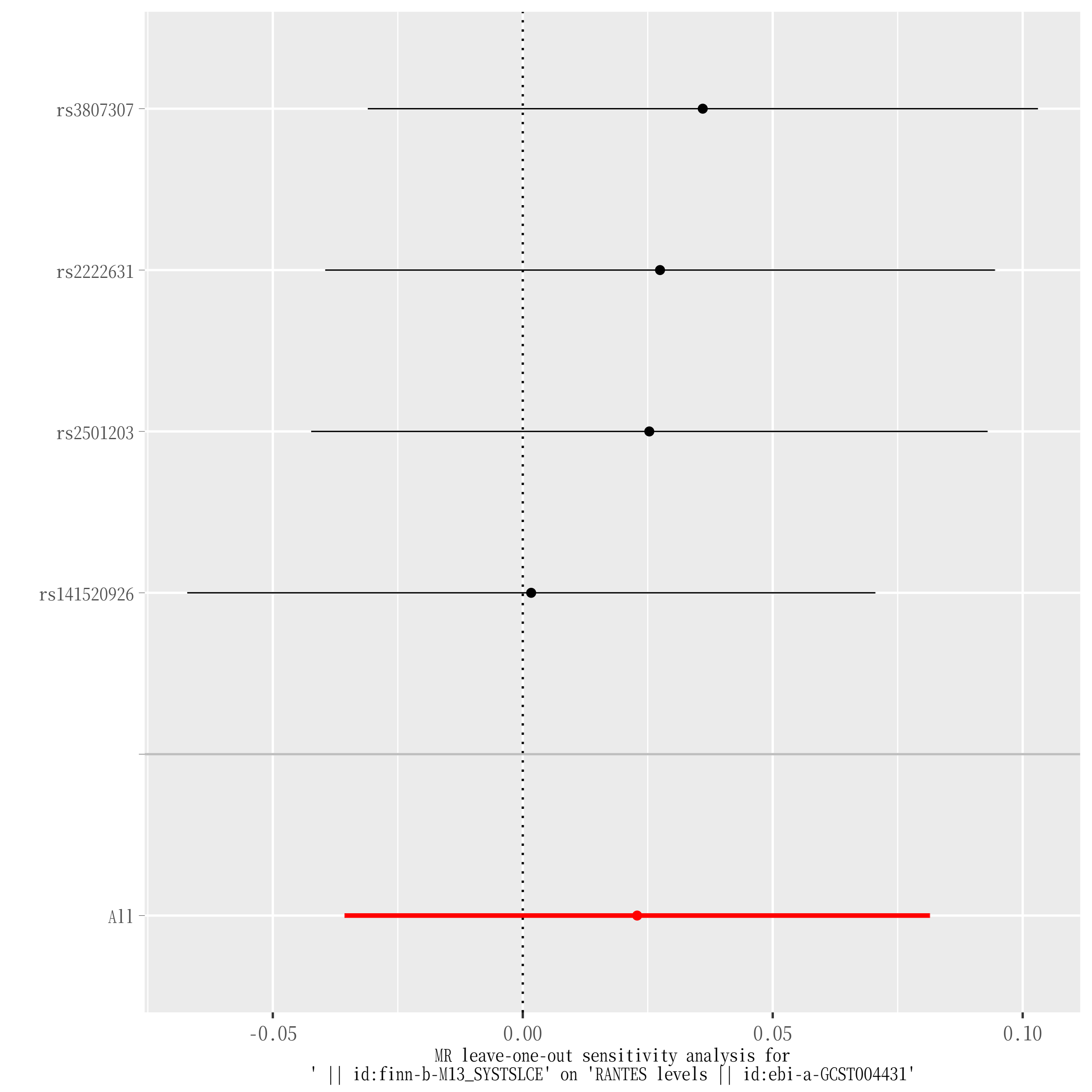

rs3807307
rs2222631
rs2501203
rs141520926
All
-0.05
0.00
0.05
0.10
MR leave-one-out sensitivity analysis for
' || id:finn-b-M13_SYSTSLCE' on 'RANTES levels || id:ebi-a-GCST004431'

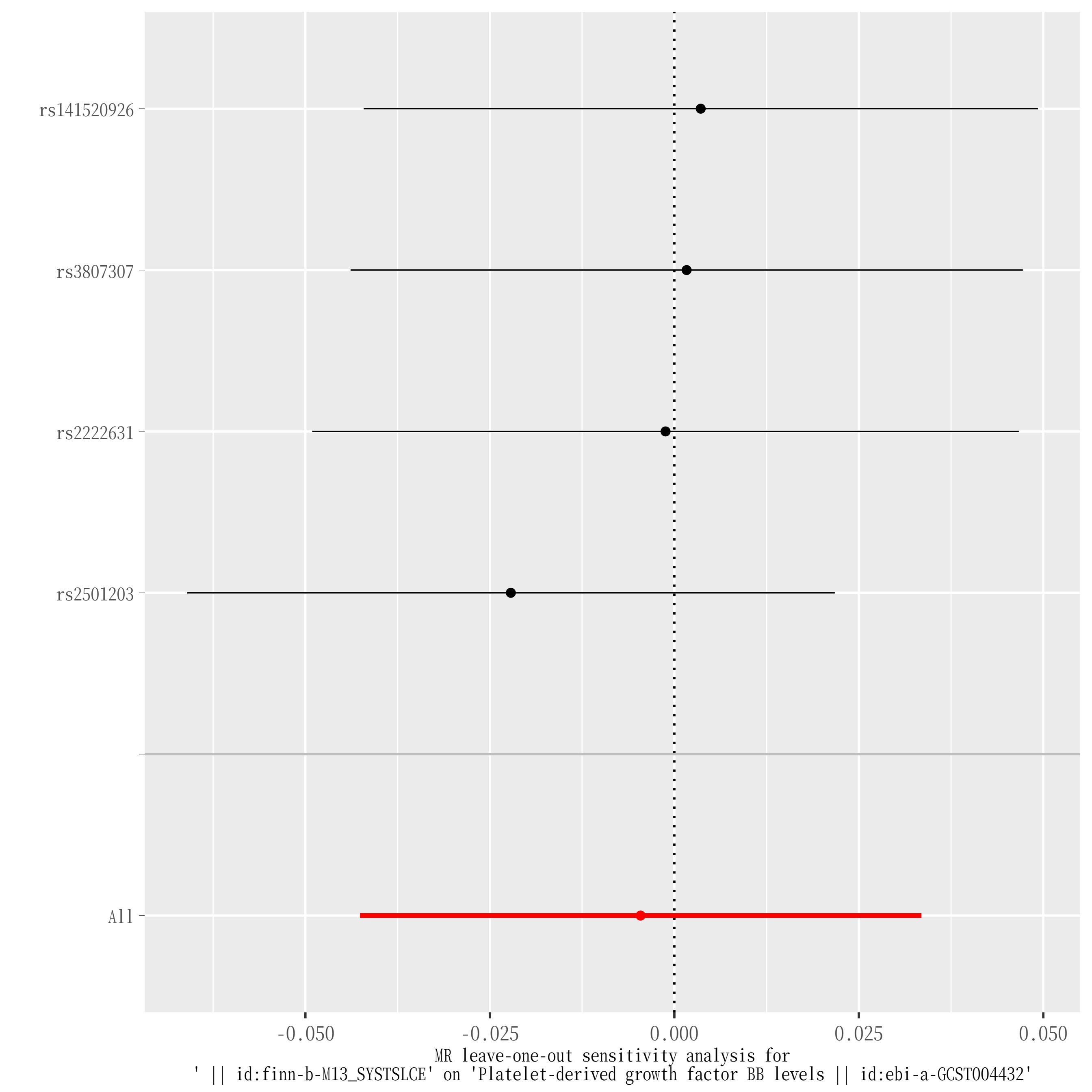

rs141520926
rs3807307
rs2222631
rs2501203
All
-0.050
-0.025
0.000
0.025
0.050
MR leave-one-out sensitivity analysis for
' || id:finn-b-M13_SYSTSLCE' on 'Platelet-derived growth factor BB levels || id:ebi-a-GCST004432'

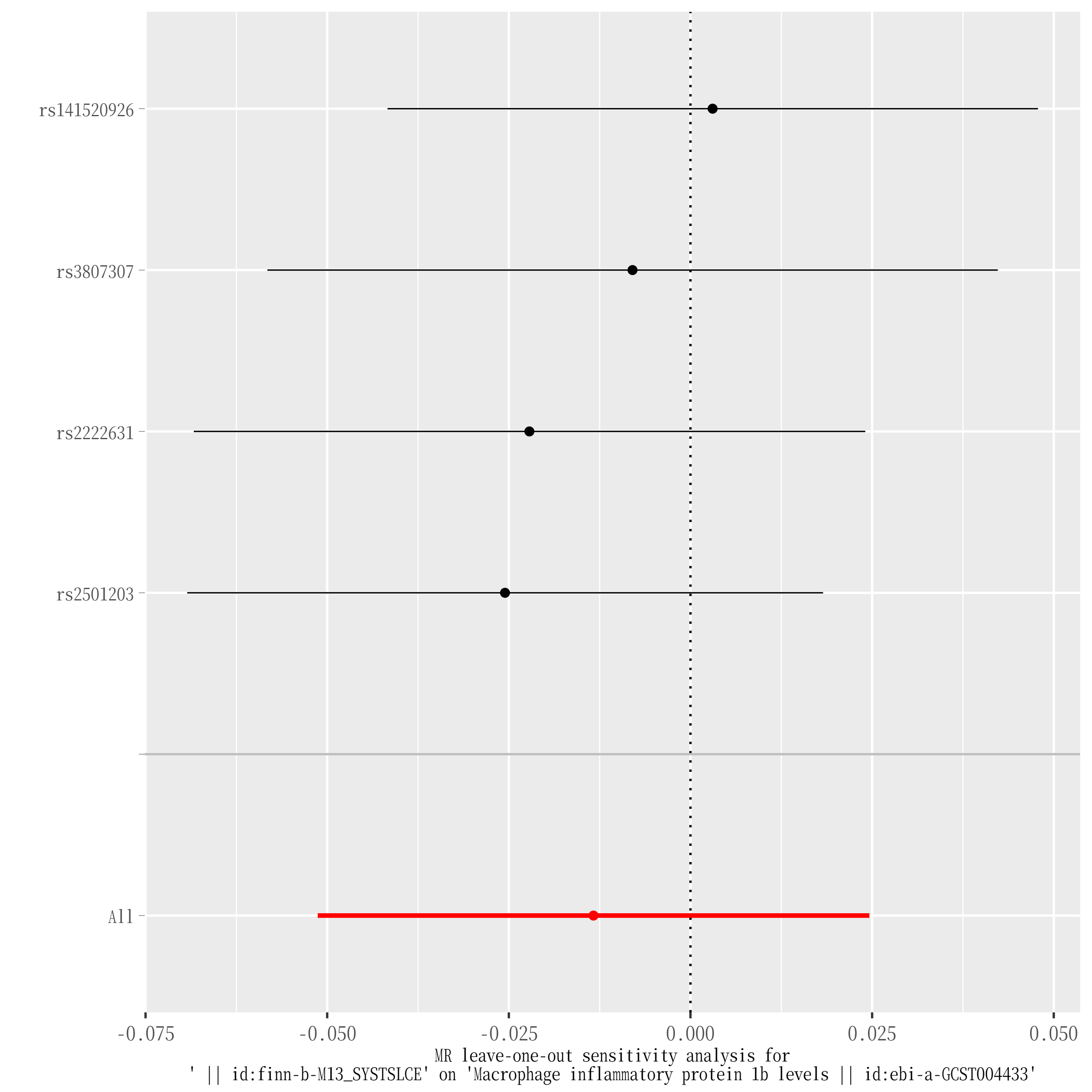

rs141520926
rs3807307
rs2222631
rs2501203
All
-0.075
-0.050
-0.025
0.000
0.025
0.050
MR leave-one-out sensitivity analysis for
' || id:finn-b-M13_SYSTSLCE' on 'Macrophage inflammatory protein 1b levels || id:ebi-a-GCST004433'

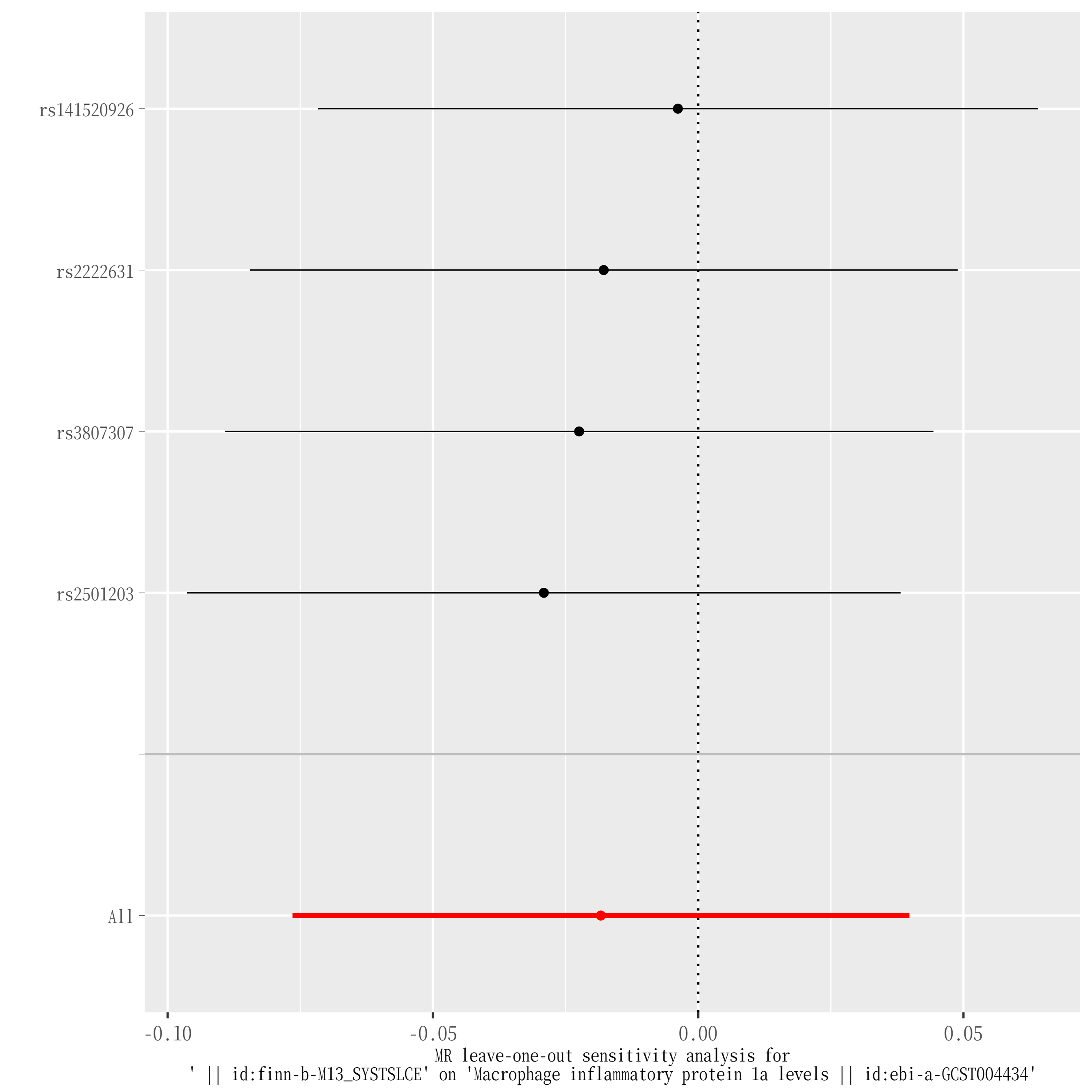
rs141520926
rs2222631
rs3807307
rs2501203
All
-0.10
-0.05
0.00
0.05
MR leave-one-out sensitivity analysis for
' || id:finn-b-M13_SYSTSLCE' on 'Macrophage inflammatory protein 1a levels || id:ebi-a-GCST004434'

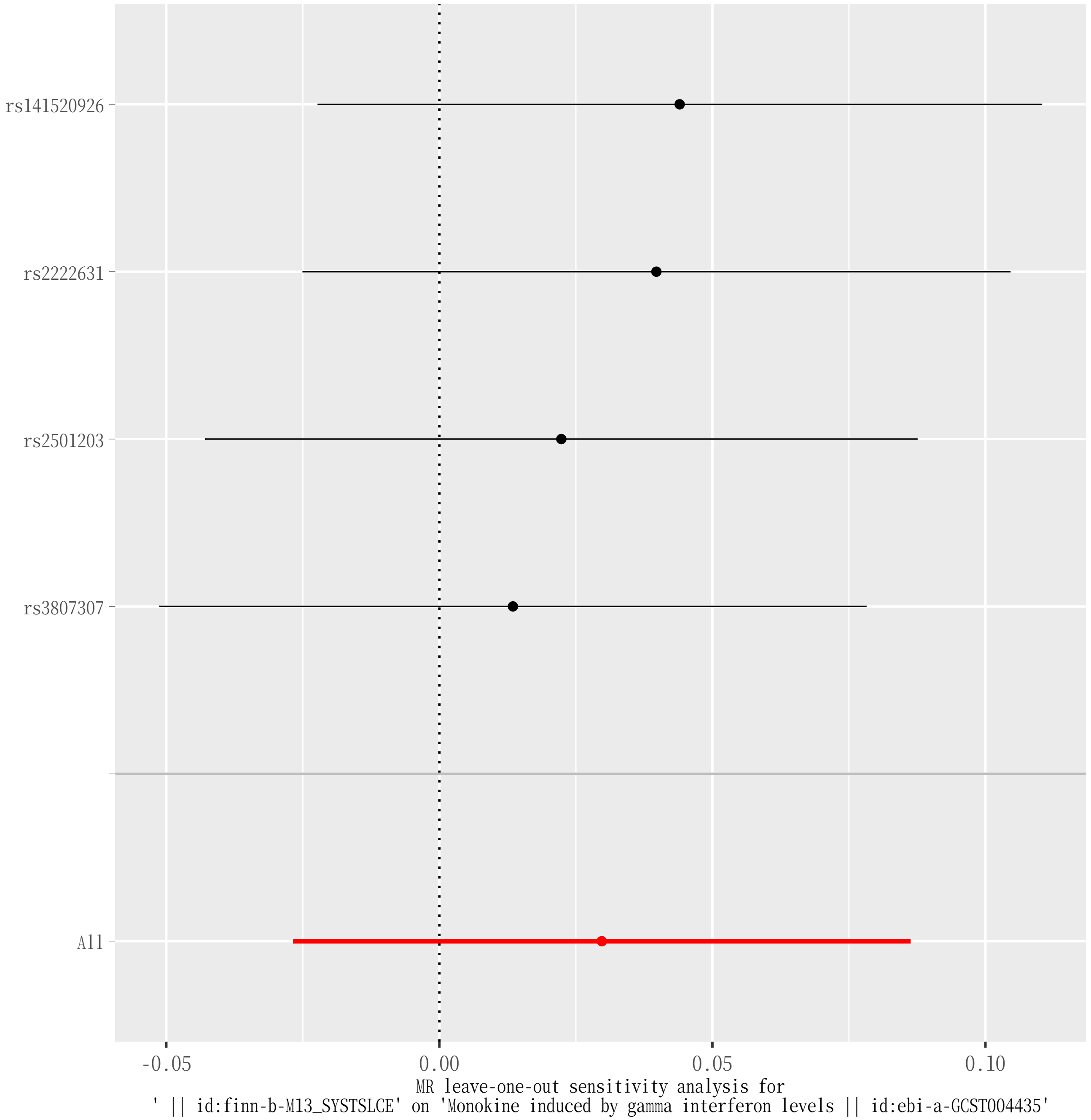

rs141520926
rs2222631
rs2501203
rs3807307
All
-0.05
0.00
0.05
0.10
MR leave-one-out sensitivity analysis for
' || id:finn-b-M13_SYSTSLCE' on 'Monokine induced by gamma interferon levels || id:ebi-a-GCST004435'

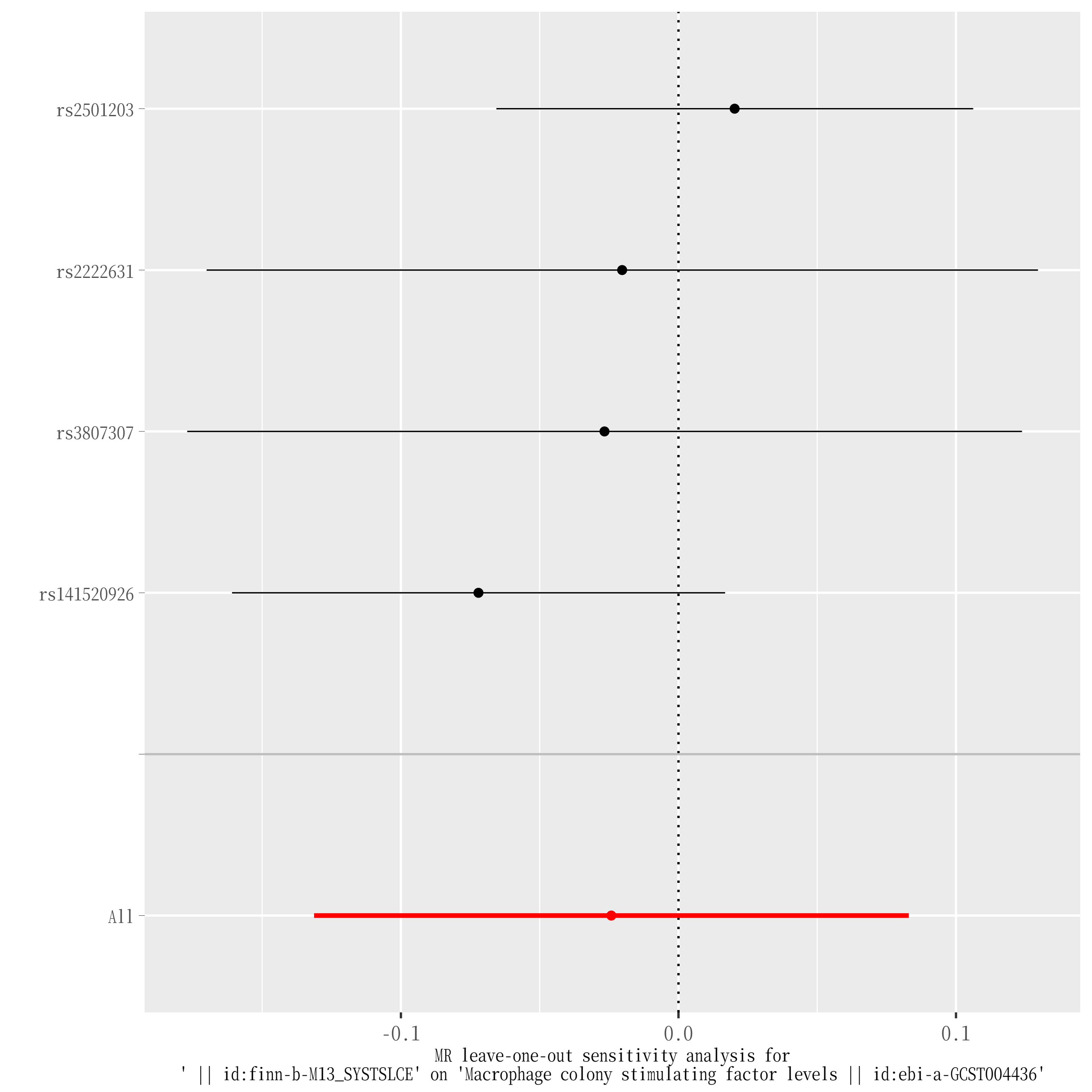
rs2501203
rs2222631
rs3807307
rs141520926
All
-0.1
0.0
0.1
MR leave-one-out sensitivity analysis for
' || id:finn-b-M13_SYSTSLCE' on 'Macrophage colony stimulating factor levels || id:ebi-a-GCST004436'

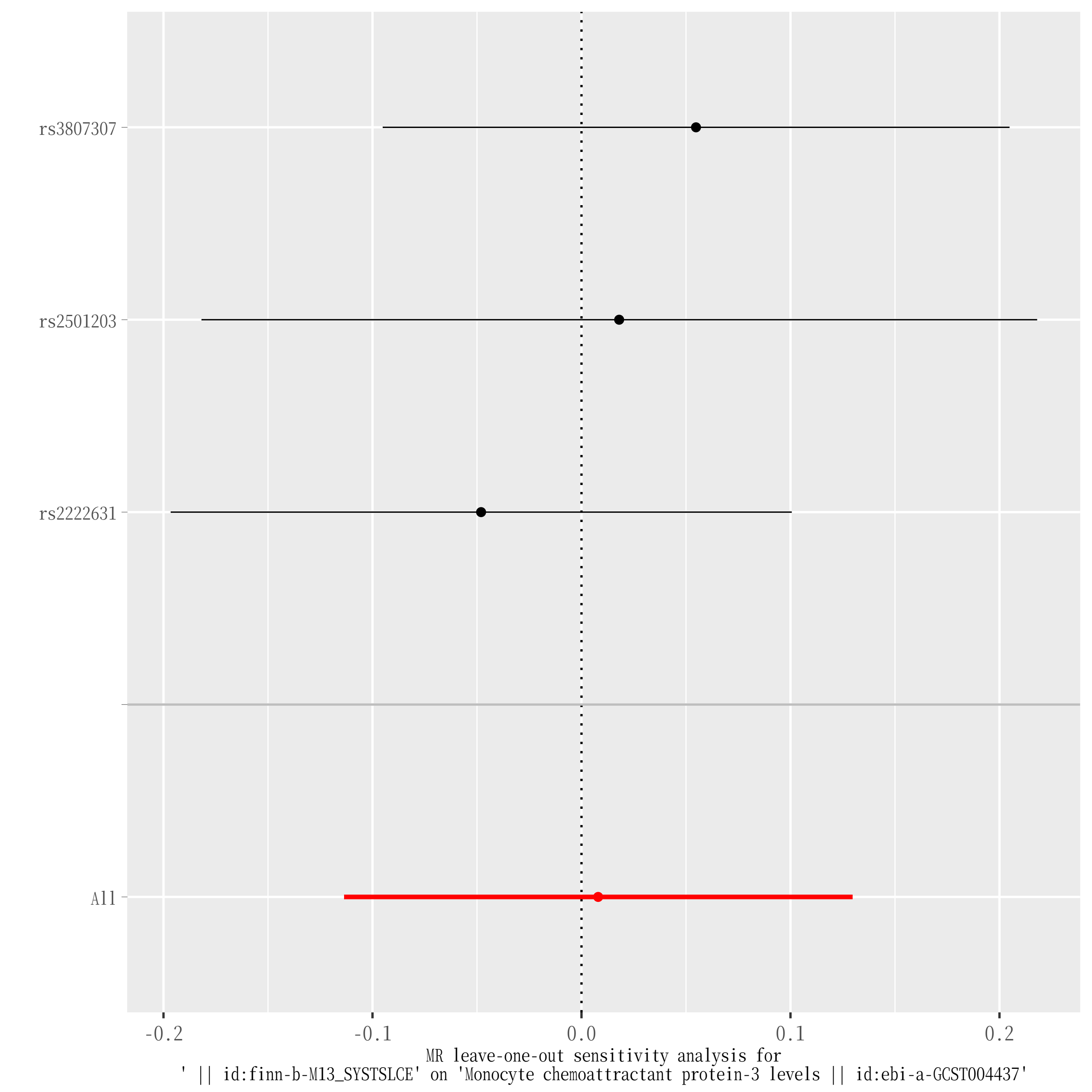

rs3807307
rs2501203
rs2222631
All
-0.2
-0.1
0.0
0.1
0.2
MR leave-one-out sensitivity analysis for
' || id:finn-b-M13_SYSTSLCE' on 'Monocyte chemoattractant protein-3 levels || id:ebi-a-GCST004437'

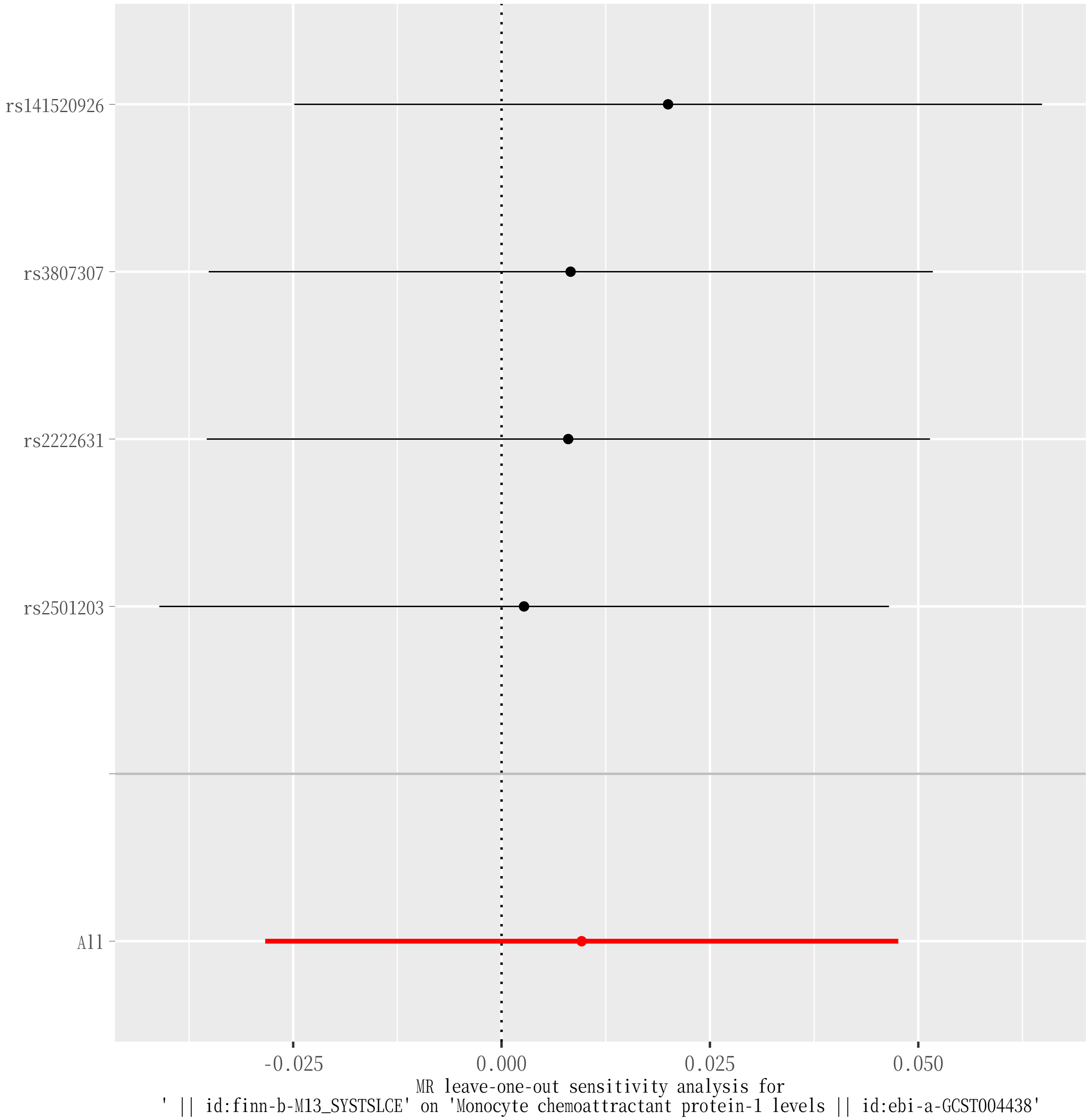
rs141520926
rs3807307
rs2222631
rs2501203
All
-0.025
0.000
0.025
0.050
MR leave-one-out sensitivity analysis for
' || id:finn-b-M13_SYSTSLCE' on 'Monocyte chemoattractant protein-1 levels || id:ebi-a-GCST004438'

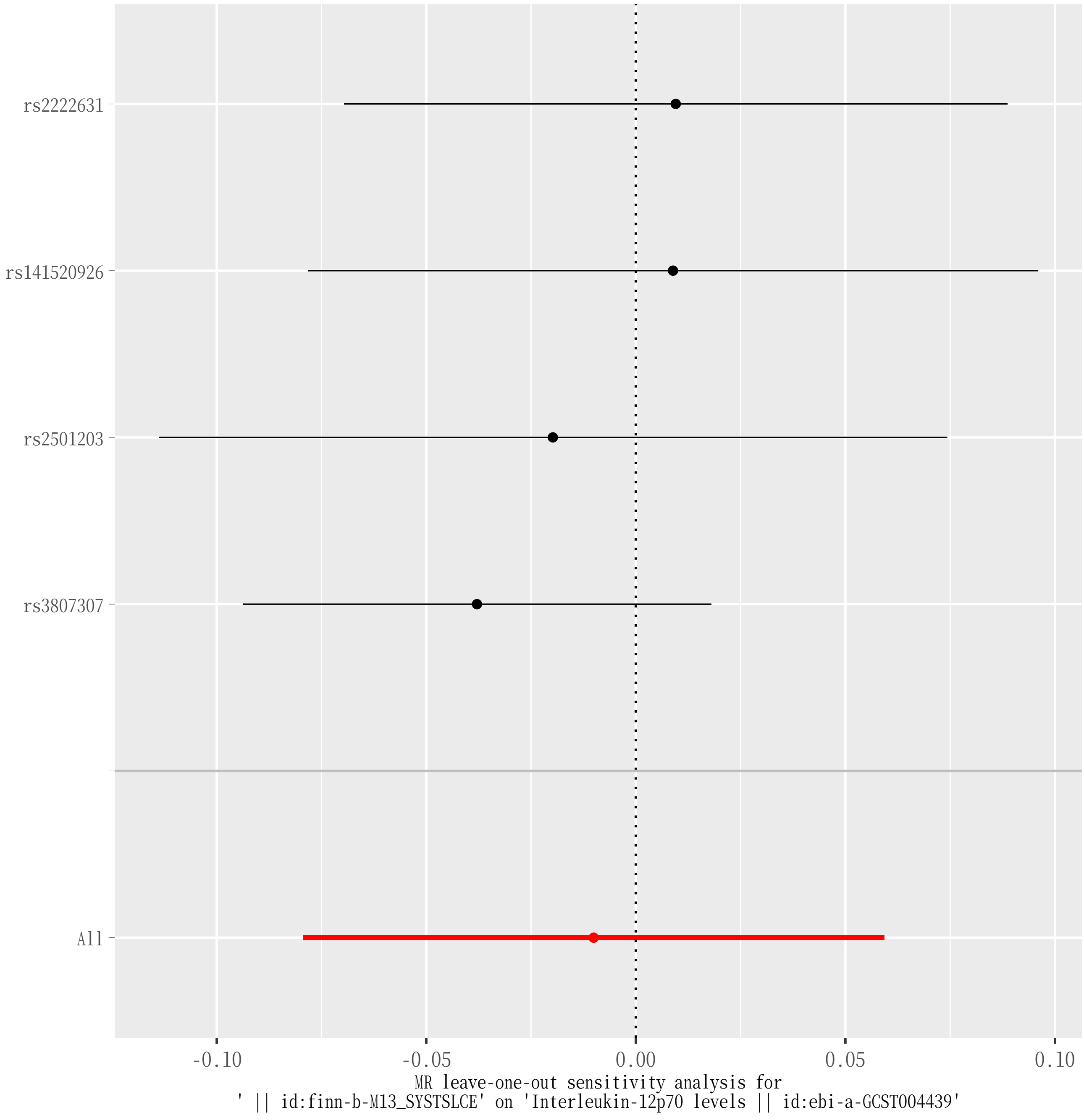
rs2222631
rs141520926
rs2501203
rs3807307
All
-0.10
-0.05
0.00
0.05
0.10
MR leave-one-out sensitivity analysis for
' || id:finn-b-M13_SYSTSLCE' on 'Interleukin-12p70 levels || id:ebi-a-GCST004439'

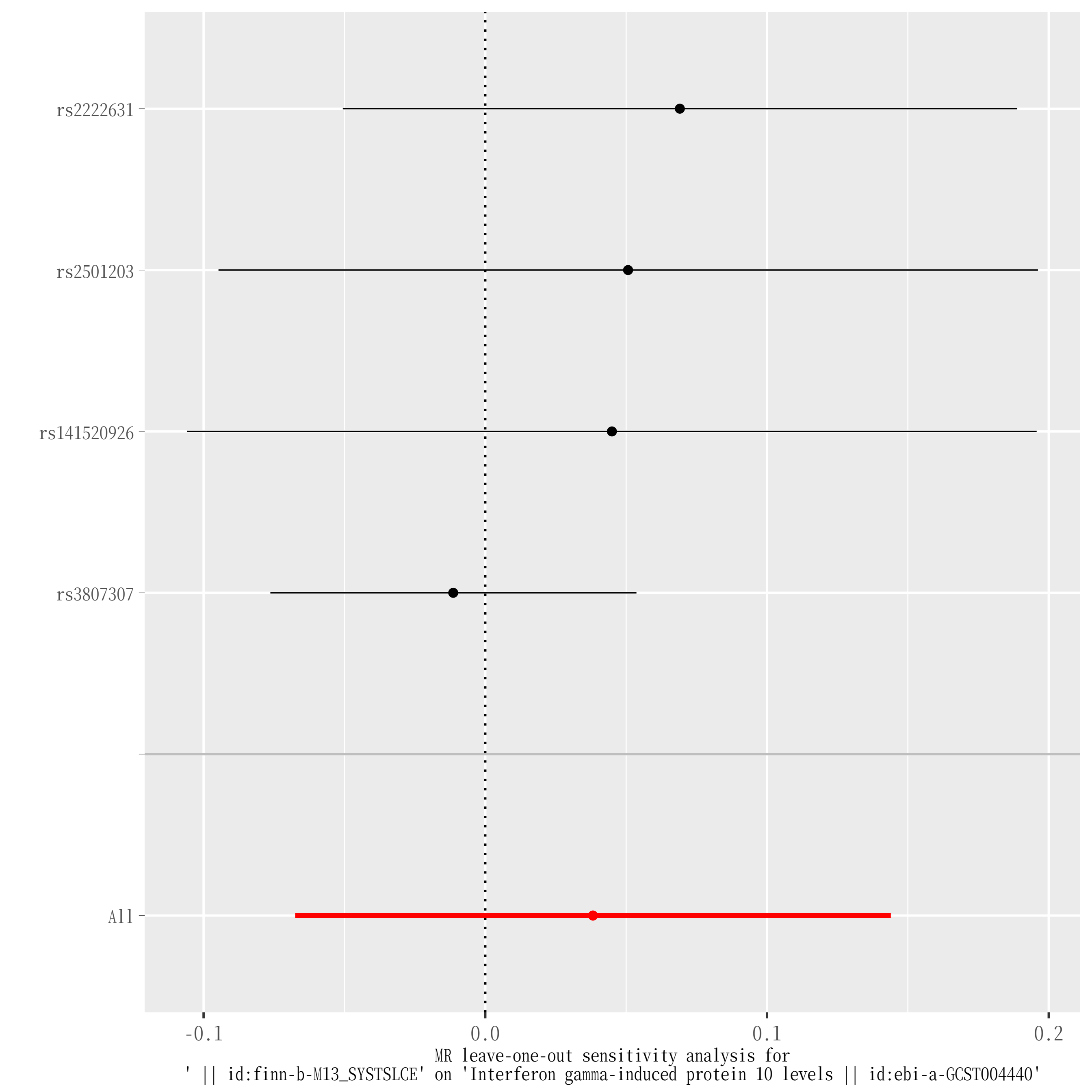

rs2222631
rs2501203
rs141520926
rs3807307
All
-0.1
0.0
0.1
0.2
MR leave-one-out sensitivity analysis for
' || id:finn-b-M13_SYSTSLCE' on 'Interferon gamma-induced protein 10 levels || id:ebi-a-GCST004440'

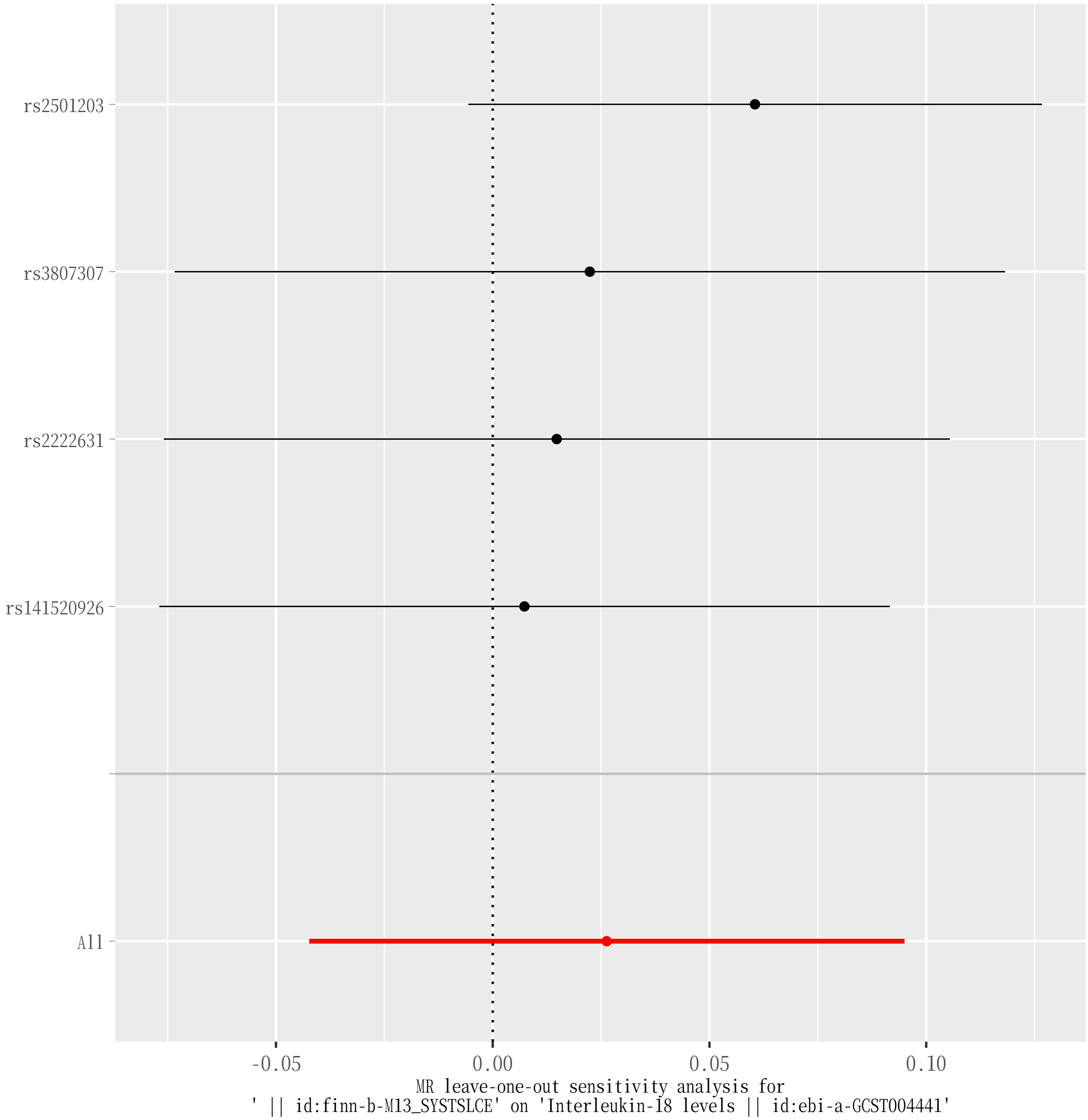
rs2501203
rs3807307
rs2222631
rs141520926
All
-0.05
0.00
0.05
0.10
MR leave-one-out sensitivity analysis for
' || id:finn-b-M13_SYSTSLCE' on 'Interleukin-18 levels || id:ebi-a-GCST004441'

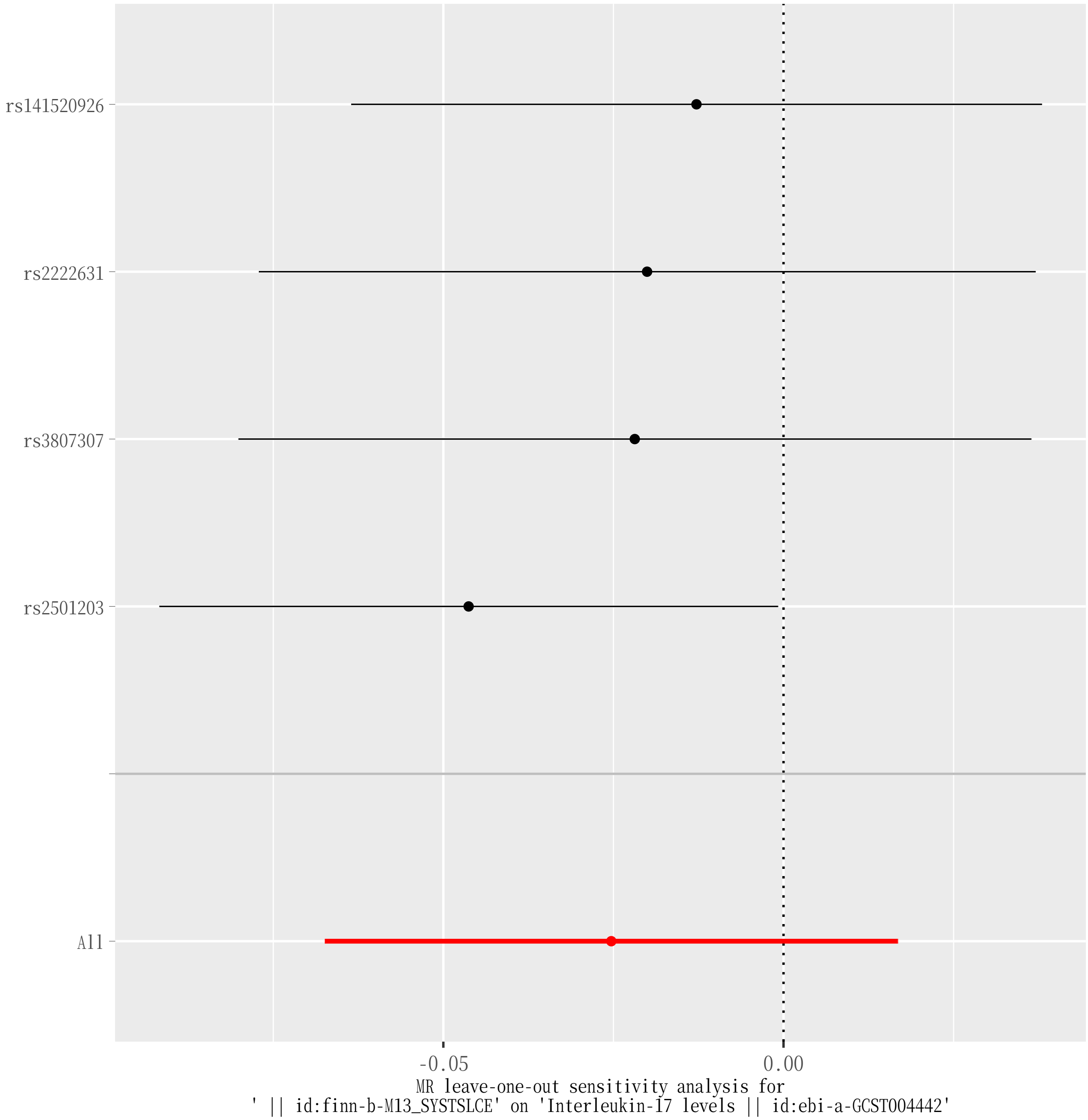
rs141520926
rs2222631
rs3807307
rs2501203
All
-0.05
0.00
MR leave-one-out sensitivity analysis for
' || id:finn-b-M13_SYSTSLCE' on 'Interleukin-17 levels || id:ebi-a-GCST004442'

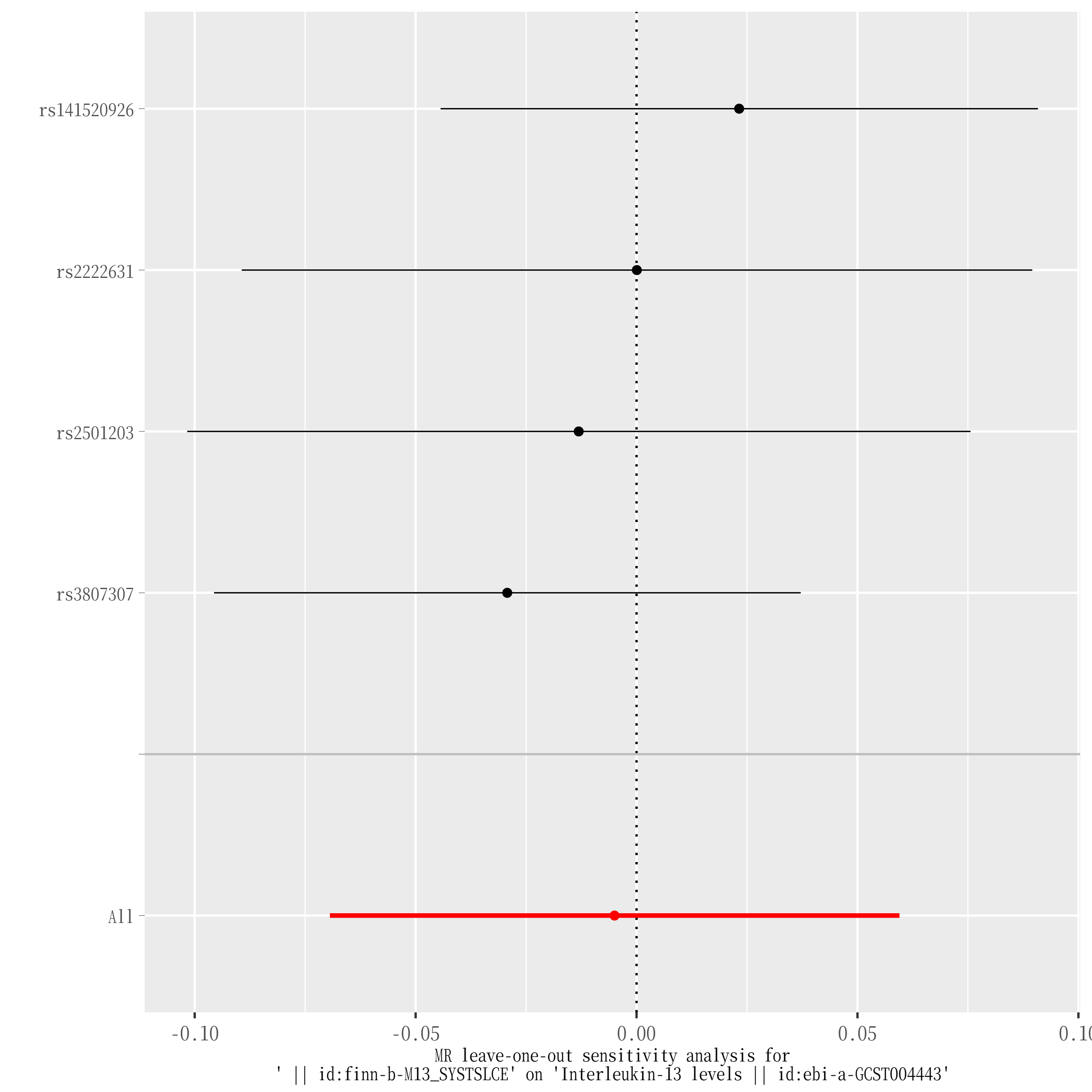
rs141520926
rs2222631
rs2501203
rs3807307
All
-0.10
-0.05
0.00
0.05
0.10
MR leave-one-out sensitivity analysis for
' || id:finn-b-M13_SYSTSLCE' on 'Interleukin-13 levels || id:ebi-a-GCST004443'

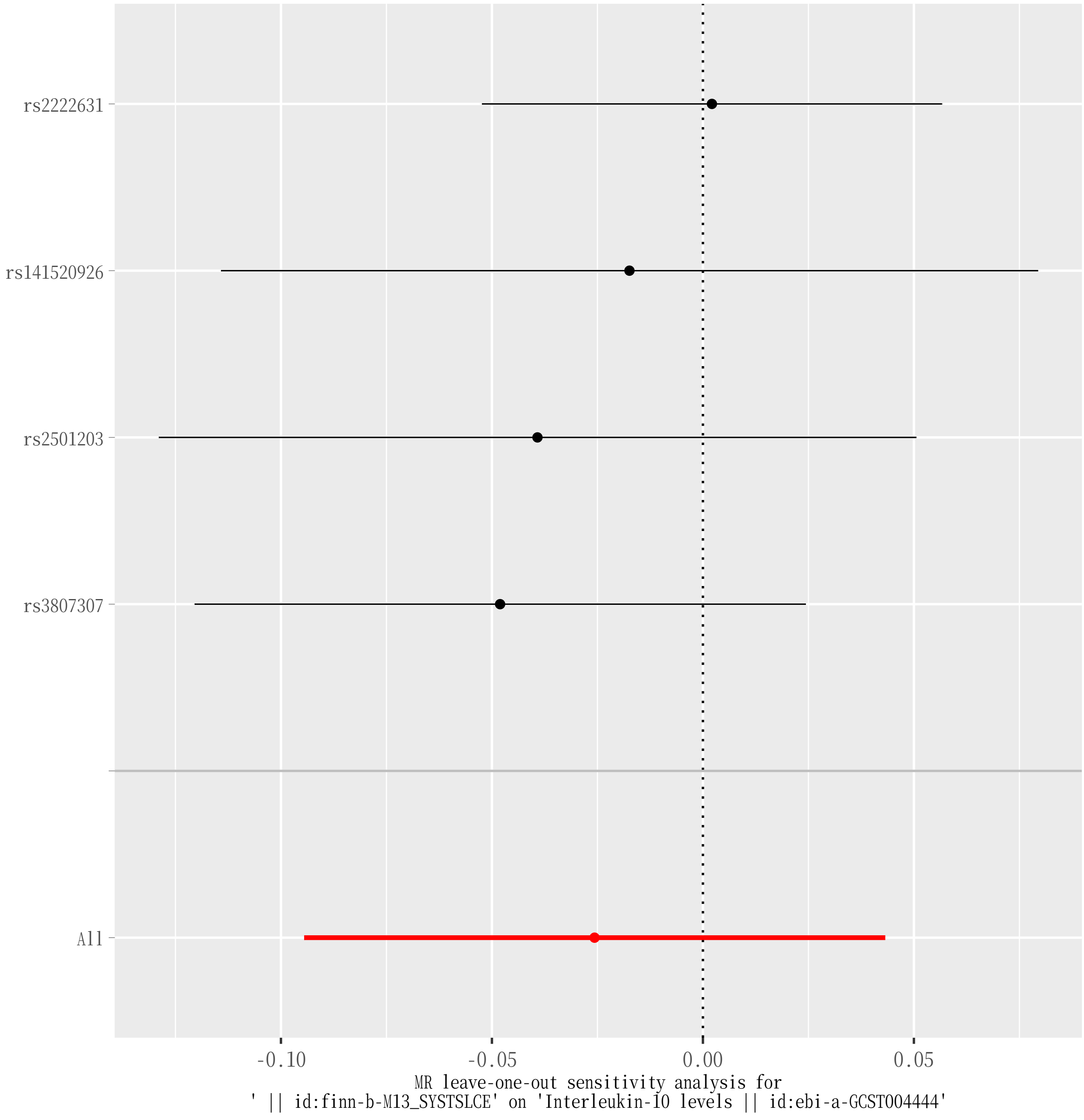

rs2222631
rs141520926
rs2501203
rs3807307
All
-0.10
-0.05
0.00
0.05
MR leave-one-out sensitivity analysis for
' || id:finn-b-M13_SYSTSLCE' on 'Interleukin-10 levels || id:ebi-a-GCST004444'

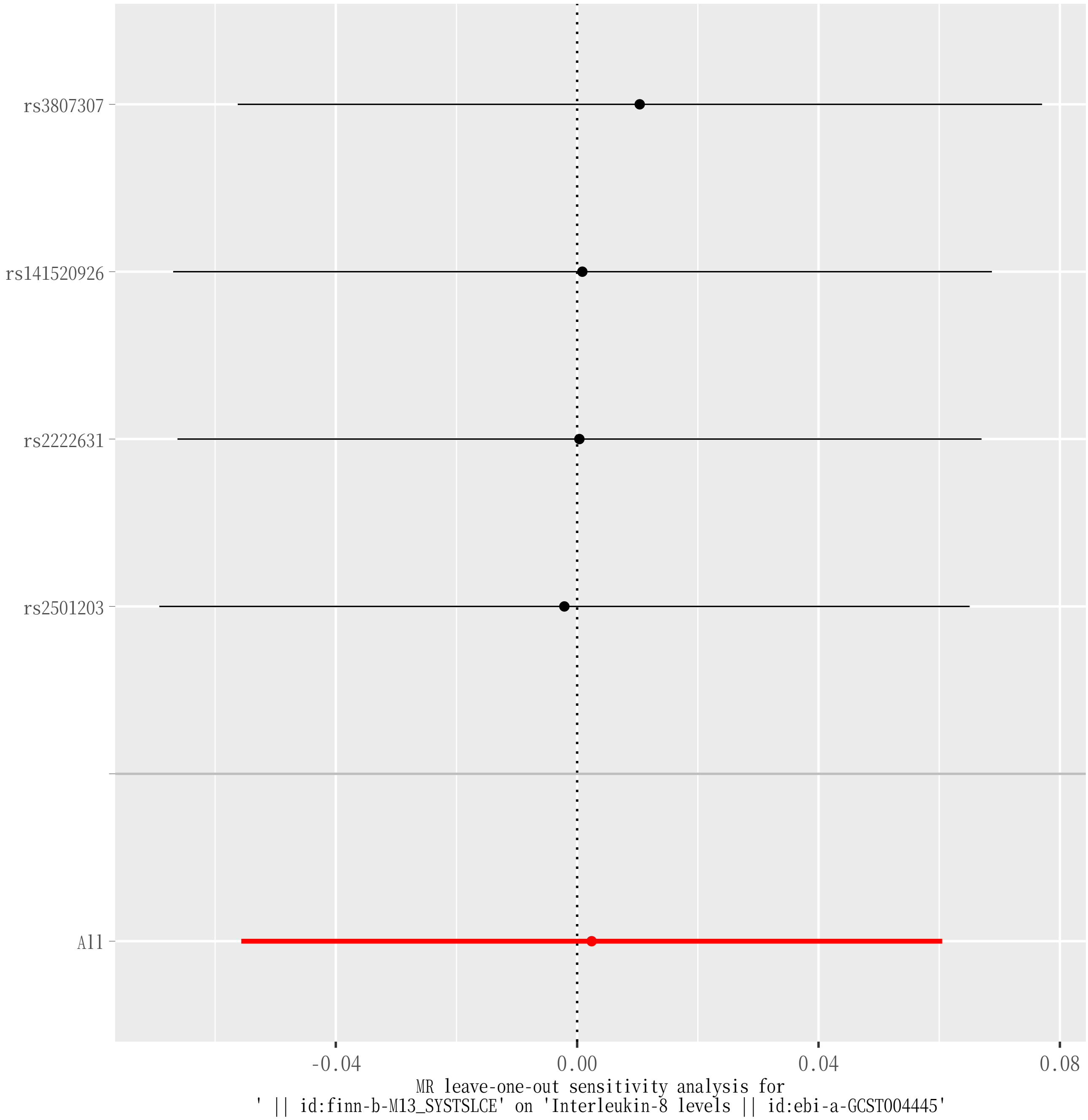
rs3807307
rs141520926
rs2222631
rs2501203
All
-0.04
0.00
0.04
0.08
MR leave-one-out sensitivity analysis for
' || id:finn-b-M13_SYSTSLCE' on 'Interleukin-8 levels || id:ebi-a-GCST004445'

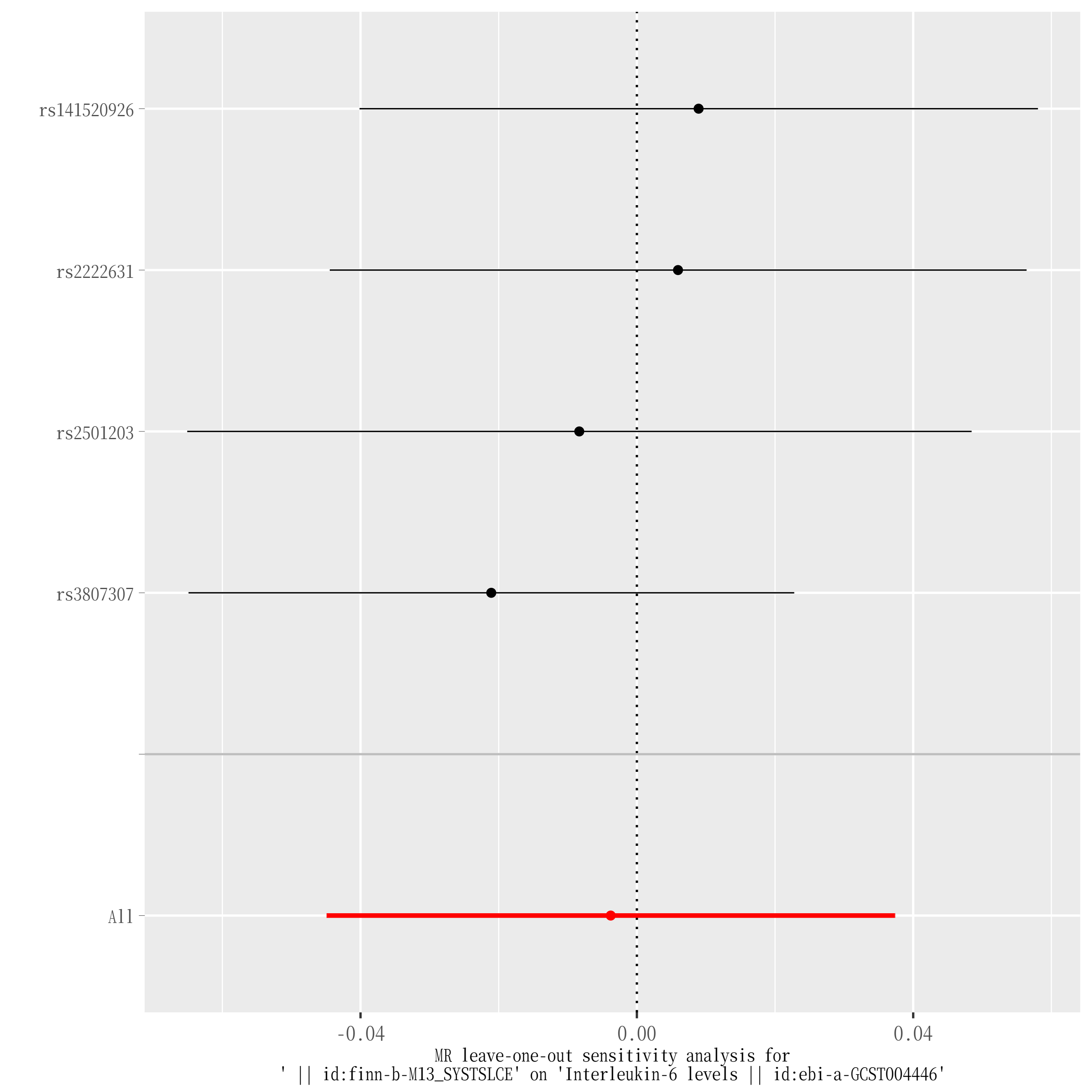
rs141520926
rs2222631
rs2501203
rs3807307
All
-0.04
0.00
0.04
MR leave-one-out sensitivity analysis for
' || id:finn-b-M13_SYSTSLCE' on 'Interleukin-6 levels || id:ebi-a-GCST004446'

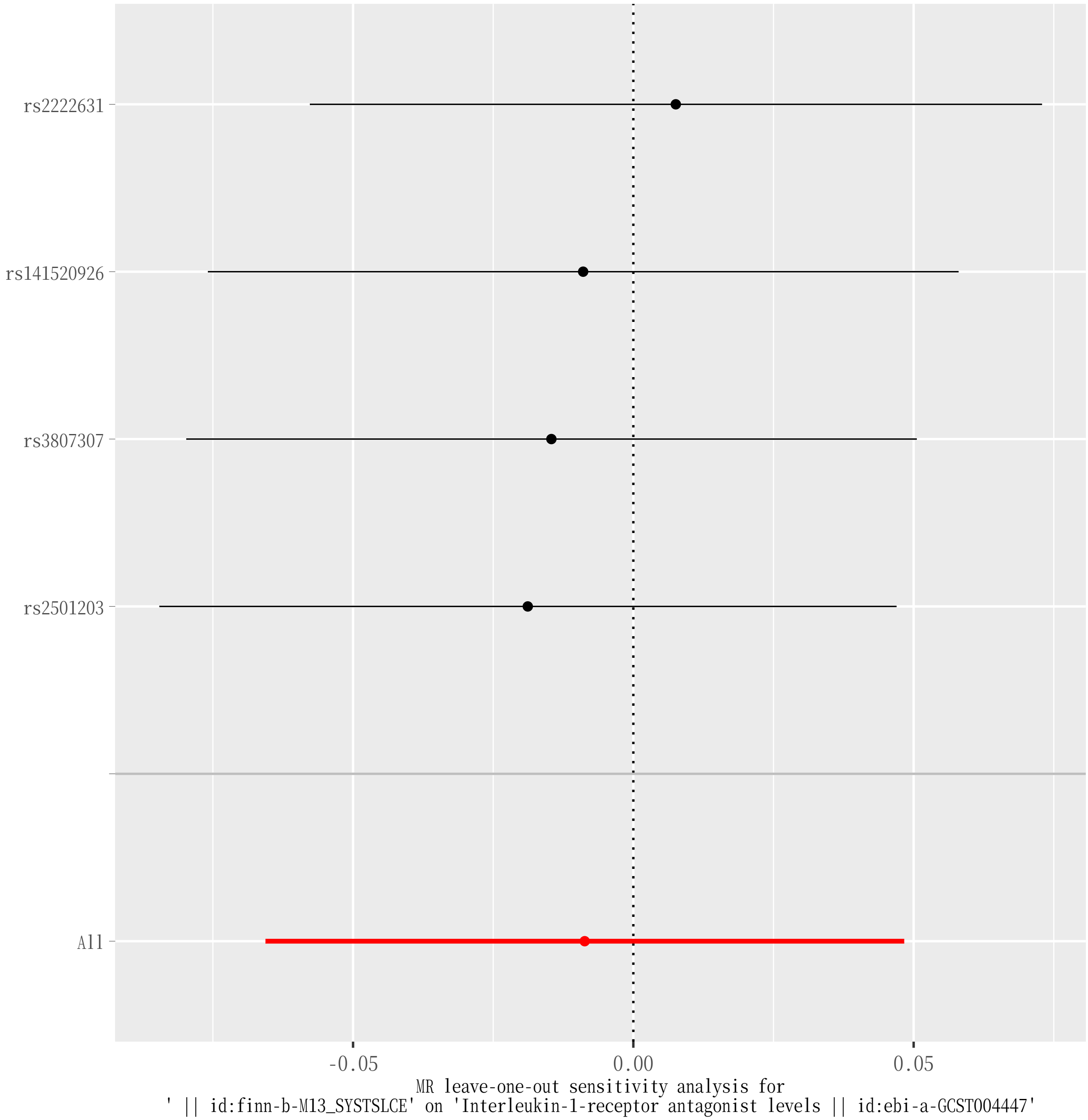
rs2222631
rs141520926
rs3807307
rs2501203
All
-0.05
0.00
0.05
MR leave-one-out sensitivity analysis for
' || id:finn-b-M13_SYSTSLCE' on 'Interleukin-1-receptor antagonist levels || id:ebi-a-GCST004447'

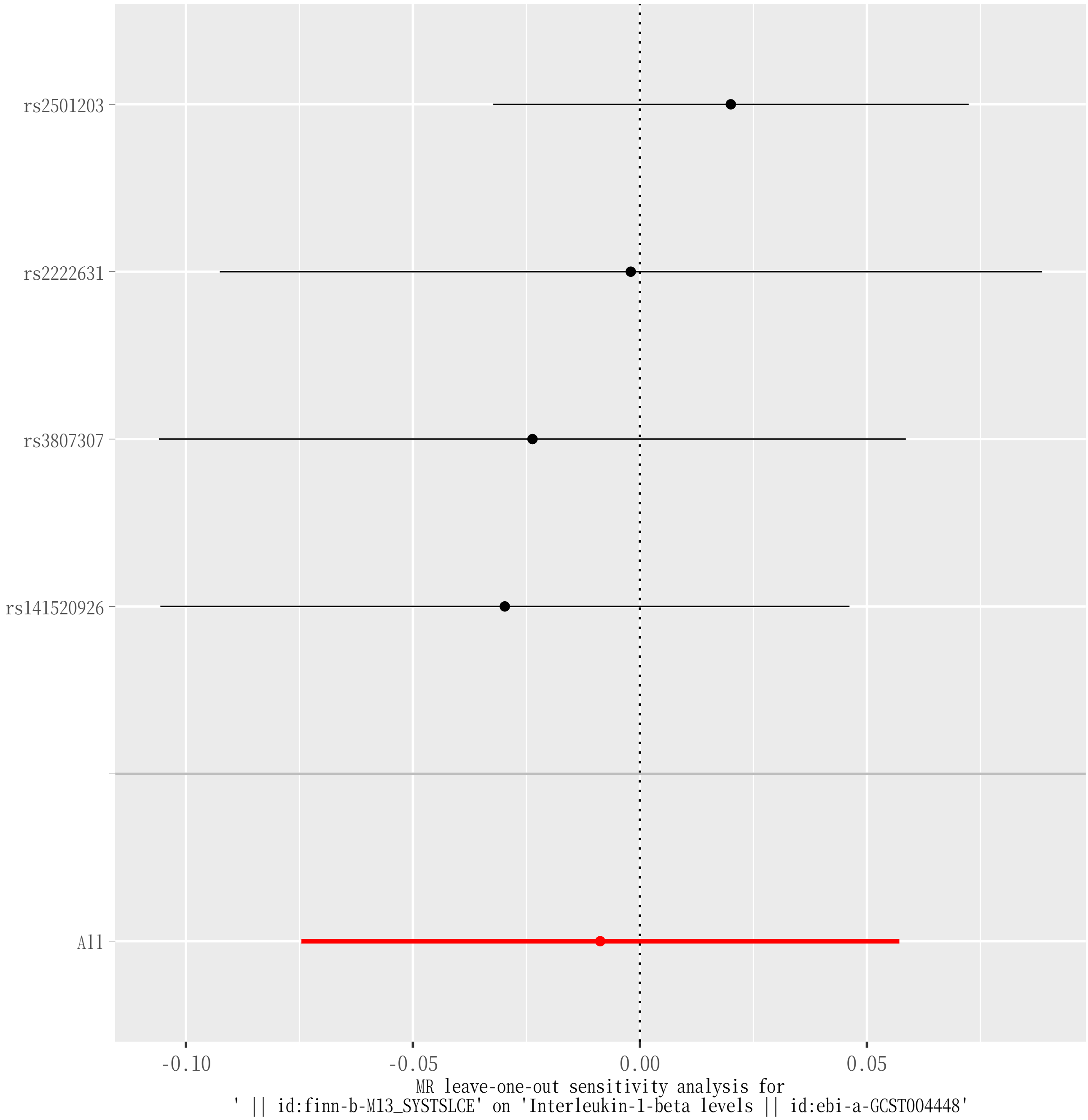
rs2501203
rs2222631
rs3807307
rs141520926
All
-0.10
-0.05
0.00
0.05
MR leave-one-out sensitivity analysis for
' || id:finn-b-M13_SYSTSLCE' on 'Interleukin-1-beta levels || id:ebi-a-GCST004448'

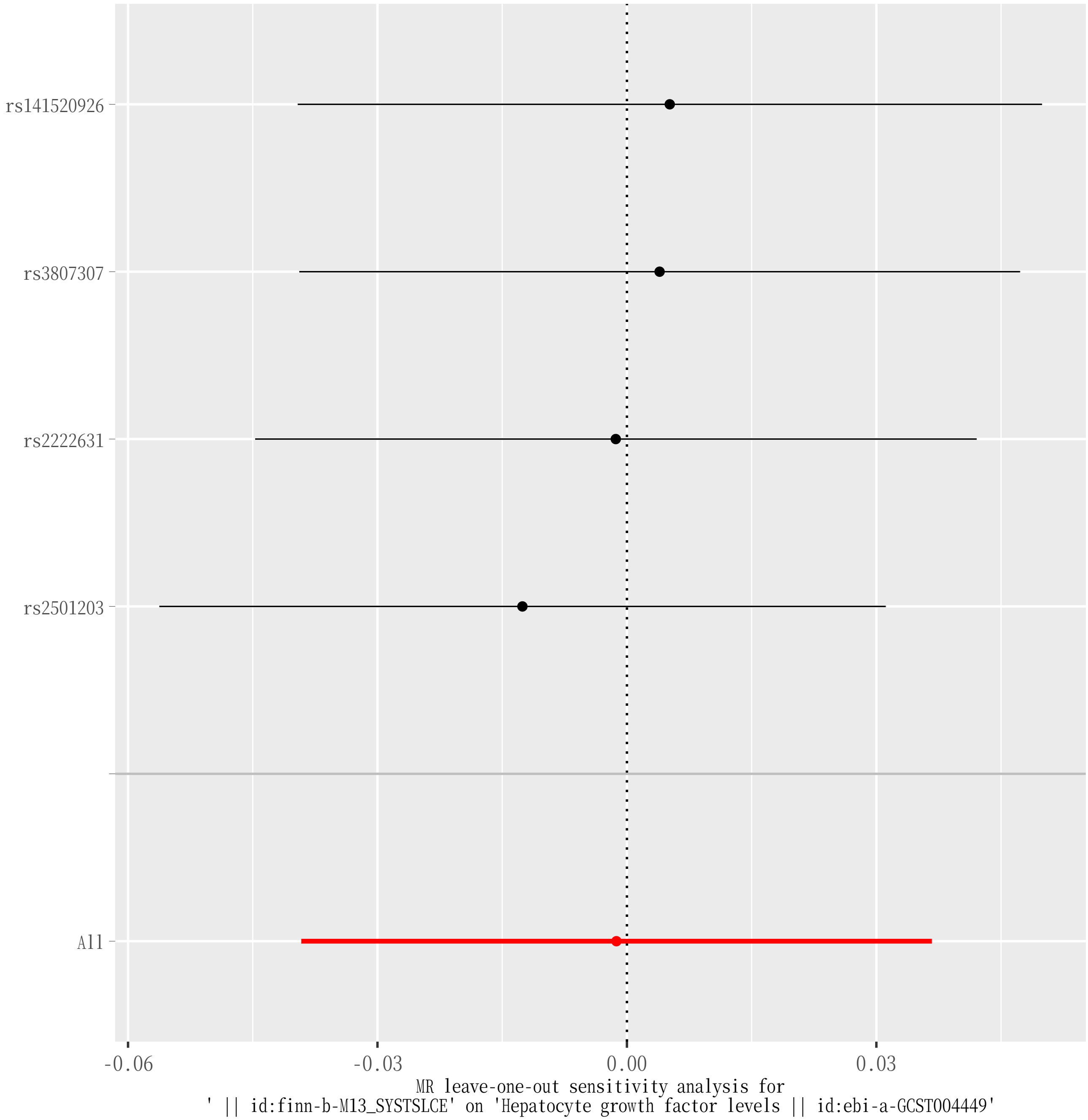
rs141520926
rs3807307
rs2222631
rs2501203
All
-0.06
-0.03
0.00
0.03
MR leave-one-out sensitivity analysis for
' || id:finn-b-M13_SYSTSLCE' on 'Hepatocyte growth factor levels || id:ebi-a-GCST004449'

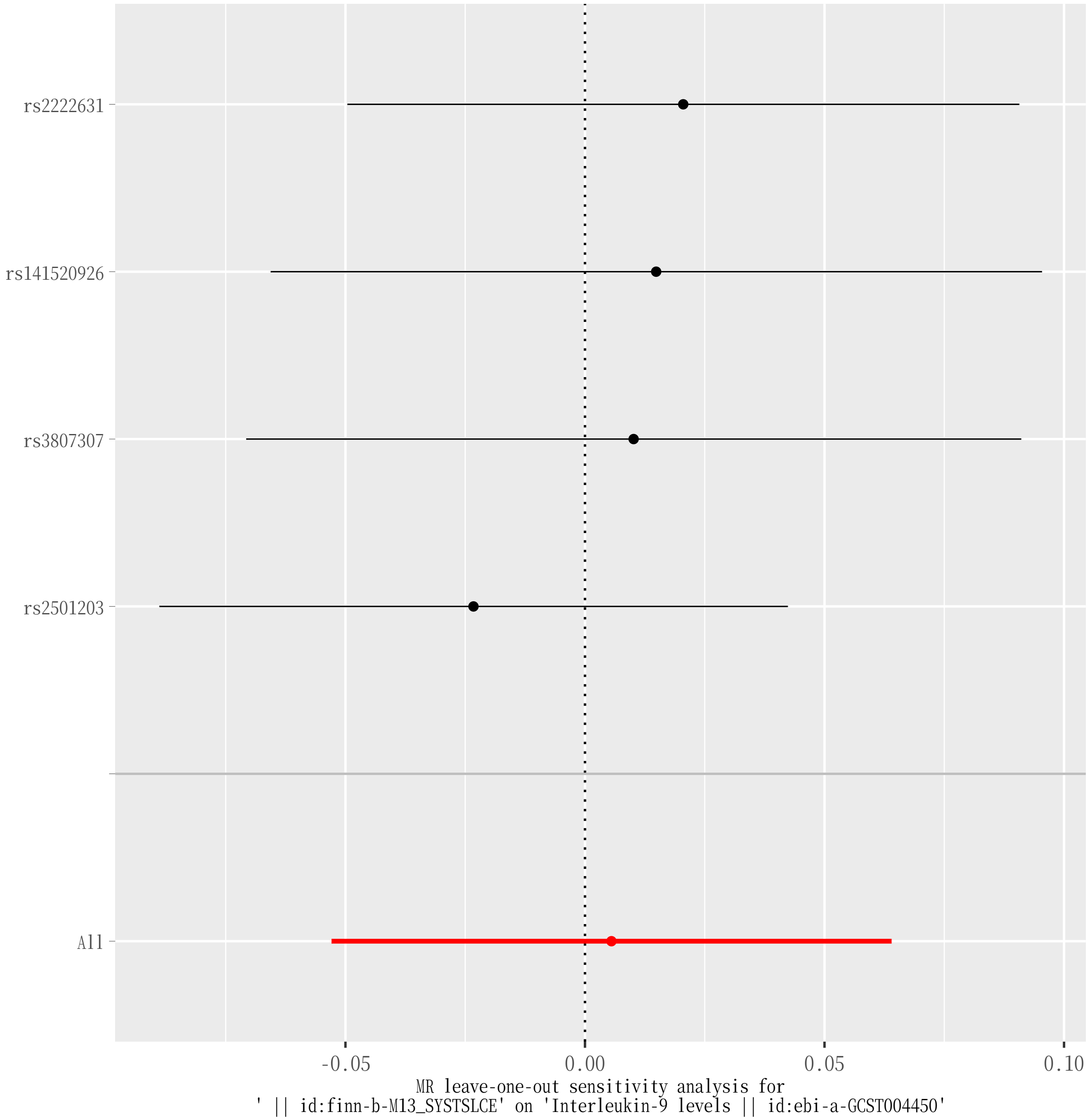
rs2222631
rs141520926
rs3807307
rs2501203
All
-0.05
0.00
0.05
0.10
MR leave-one-out sensitivity analysis for
' || id:finn-b-M13_SYSTSLCE' on 'Interleukin-9 levels || id:ebi-a-GCST004450'

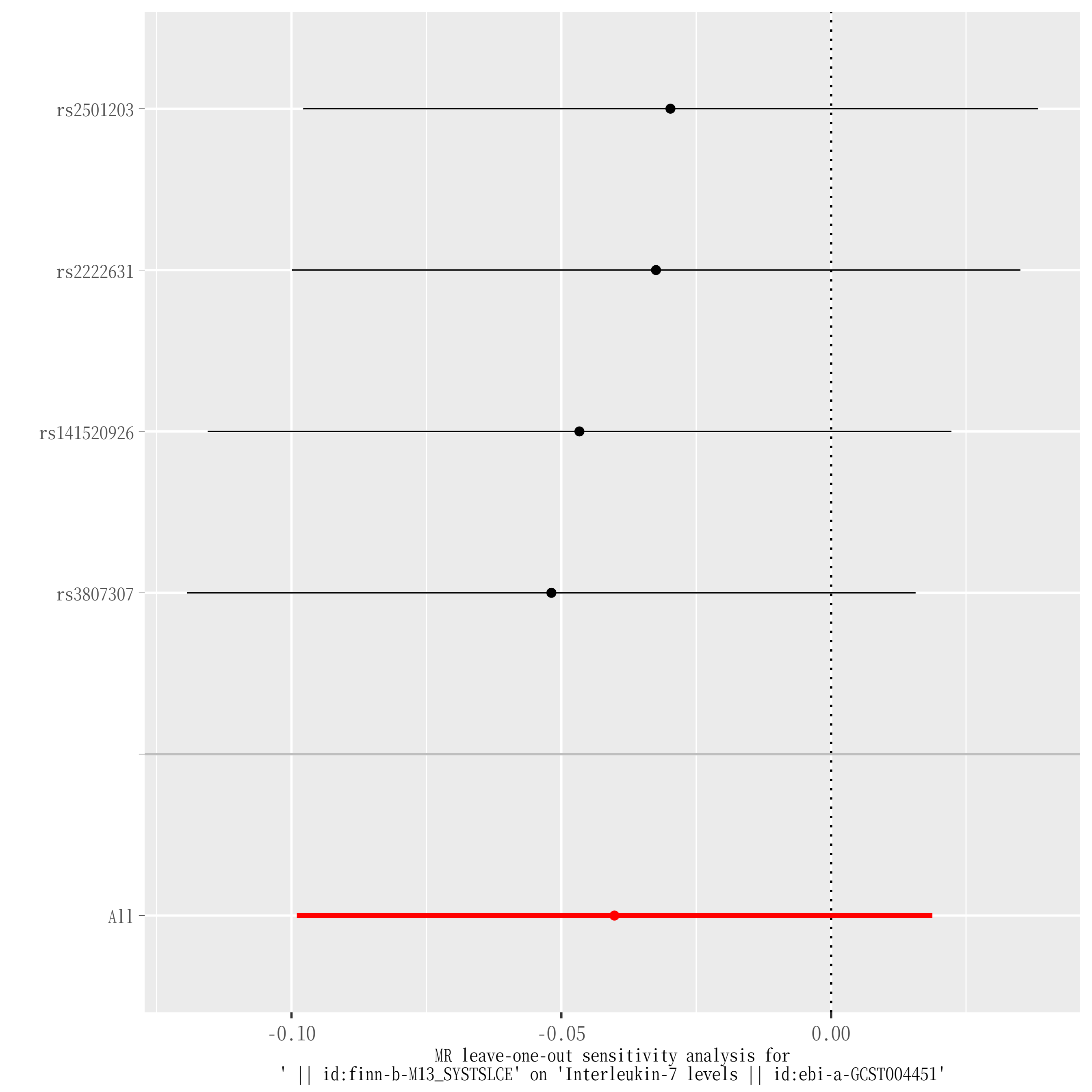
rs2501203
rs2222631
rs141520926
rs3807307
All
-0.10
-0.05
0.00
MR leave-one-out sensitivity analysis for
' || id:finn-b-M13_SYSTSLCE' on 'Interleukin-7 levels || id:ebi-a-GCST004451'

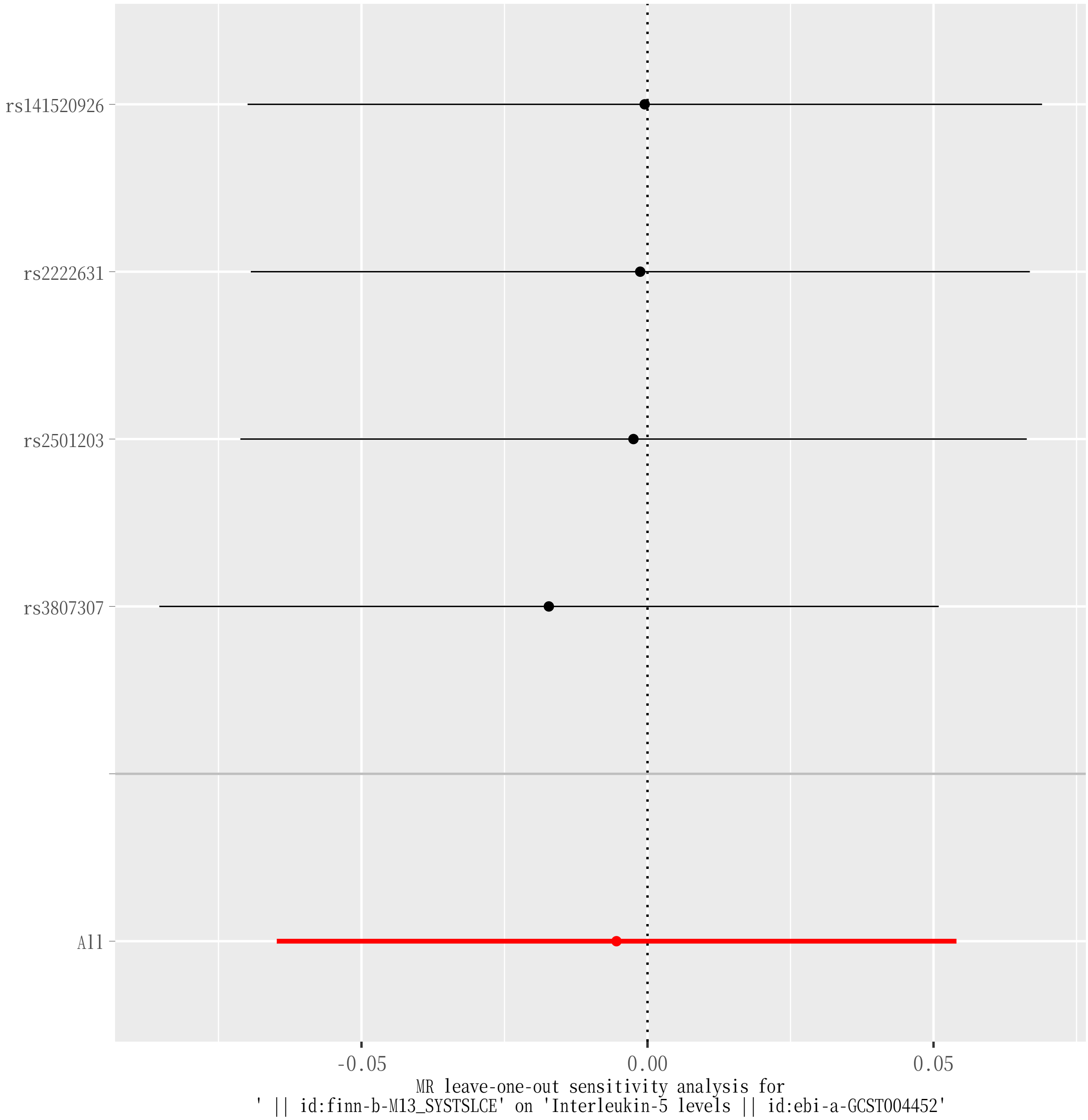
rs141520926
rs2222631
rs2501203
rs3807307
All
-0.05
0.00
0.05
MR leave-one-out sensitivity analysis for
' || id:finn-b-M13_SYSTSLCE' on 'Interleukin-5 levels || id:ebi-a-GCST004452'

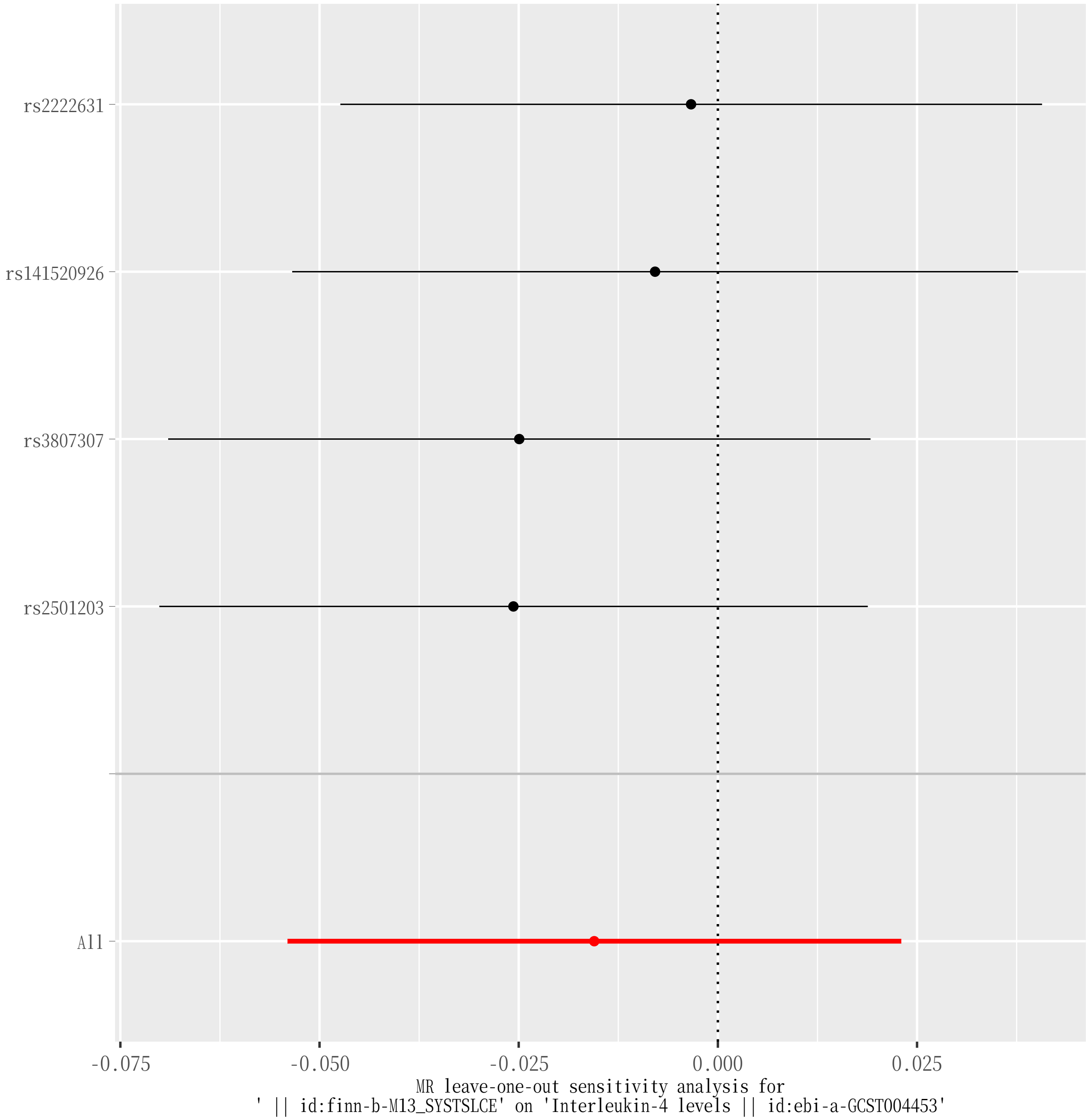

rs2222631
rs141520926
rs3807307
rs2501203
All
-0.075
-0.050
-0.025
0.000
0.025
MR leave-one-out sensitivity analysis for
' || id:finn-b-M13_SYSTSLCE' on 'Interleukin-4 levels || id:ebi-a-GCST004453'

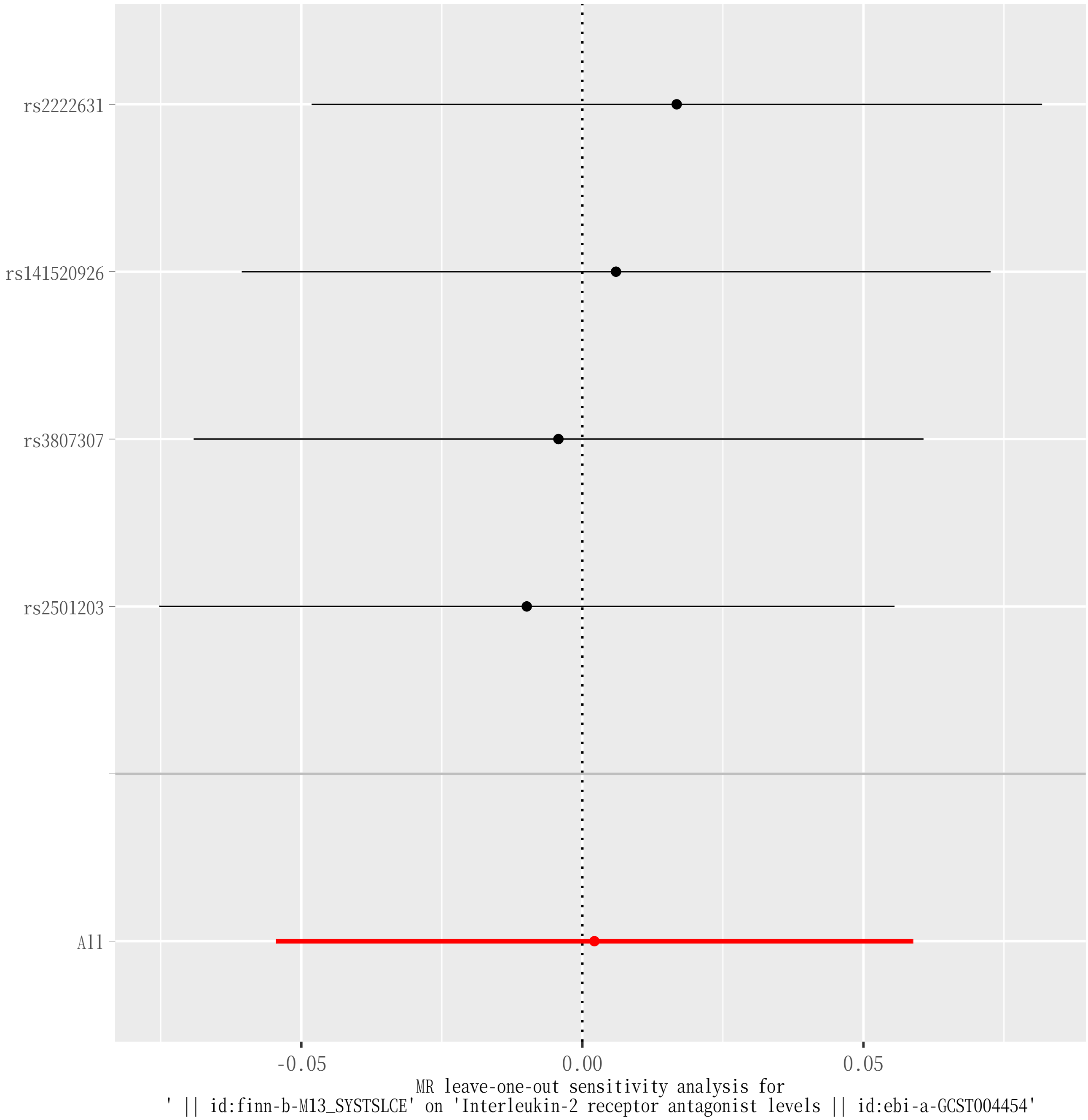
rs2222631
rs141520926
rs3807307
rs2501203
All
-0.05
0.00
0.05
MR leave-one-out sensitivity analysis for
' || id:finn-b-M13_SYSTSLCE' on 'Interleukin-2 receptor antagonist levels || id:ebi-a-GCST004454'

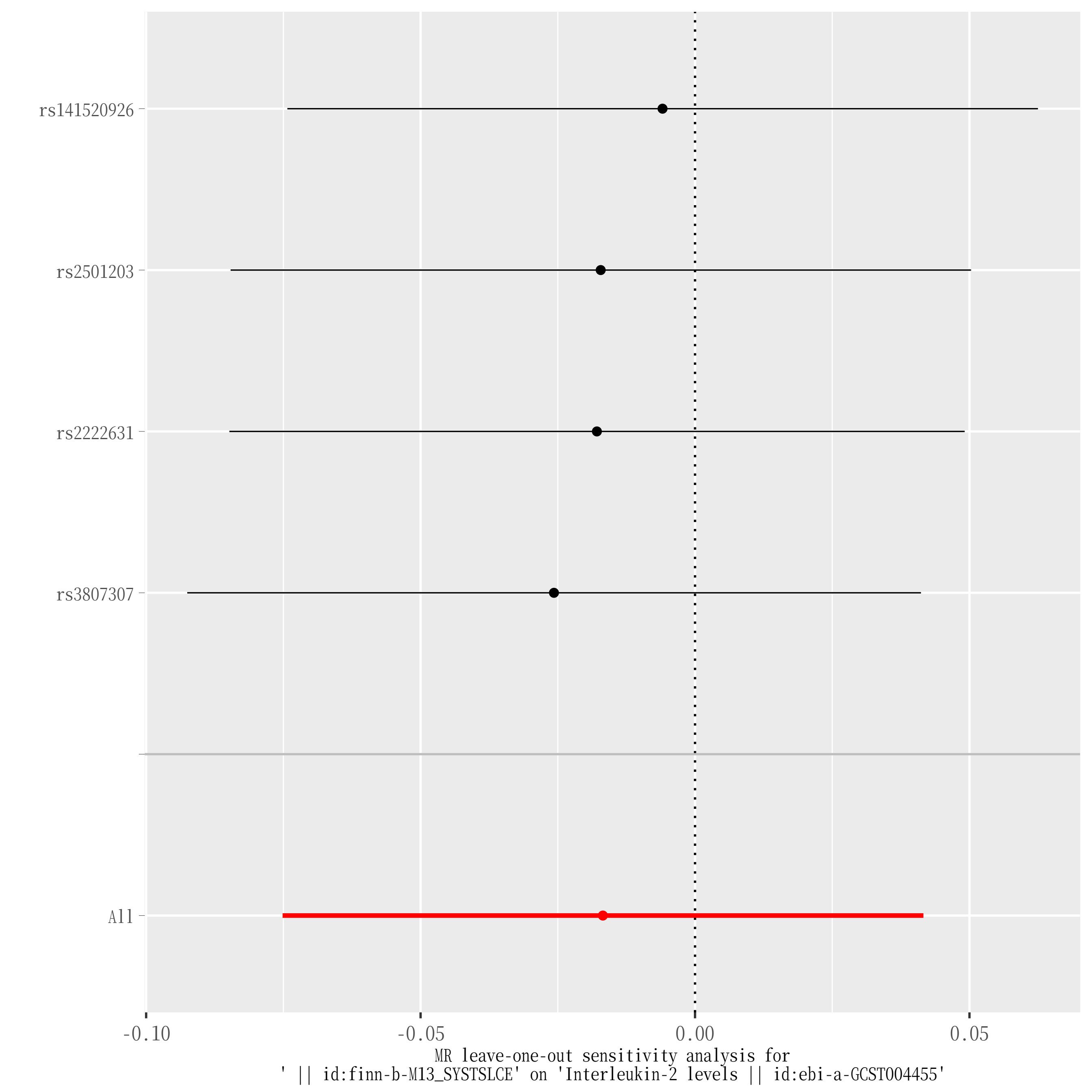

rs141520926
rs2501203
rs2222631
rs3807307
All
-0.10
-0.05
0.00
0.05
MR leave-one-out sensitivity analysis for
' || id:finn-b-M13_SYSTSLCE' on 'Interleukin-2 levels || id:ebi-a-GCST004455'

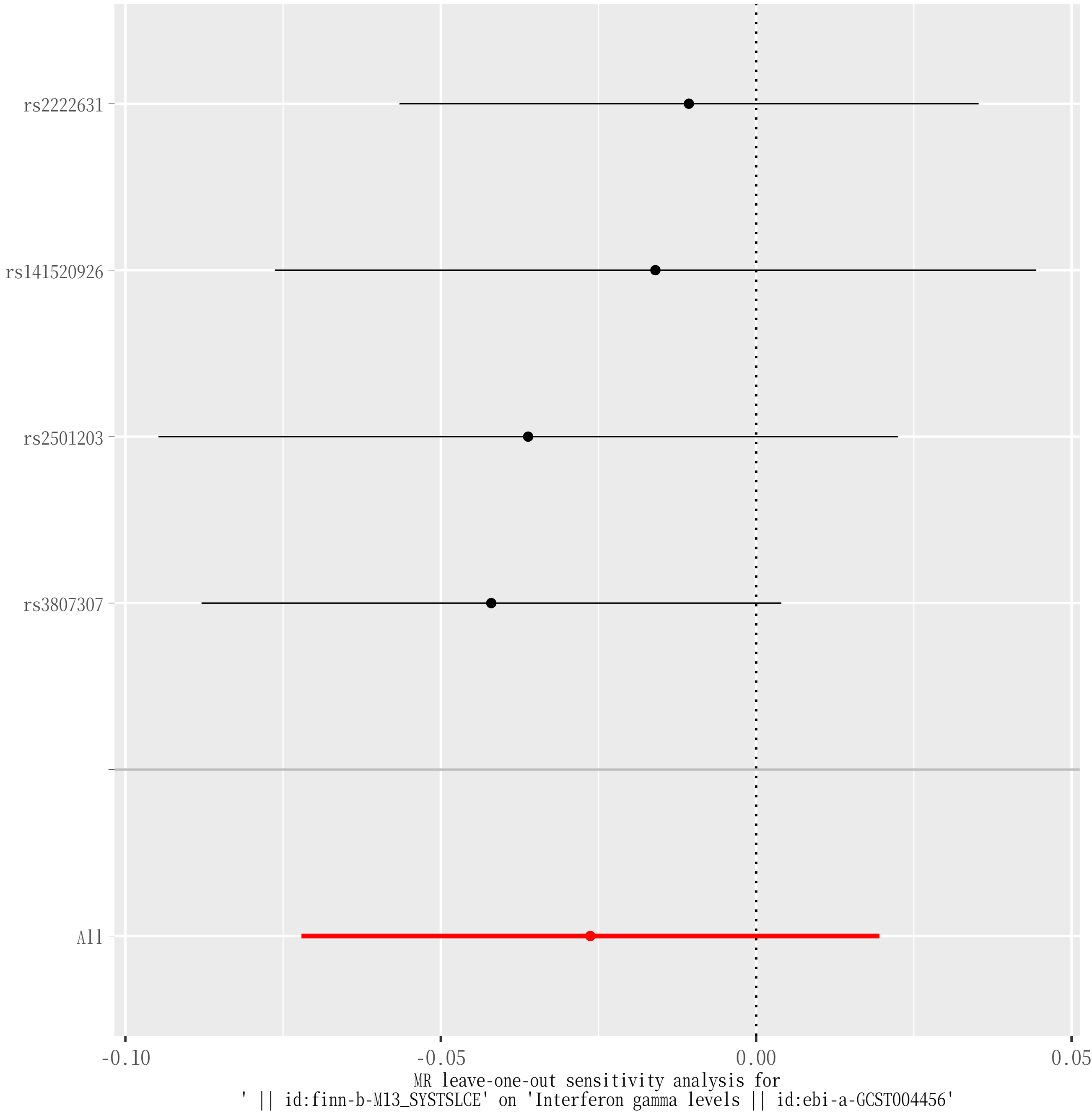
rs2222631
rs141520926
rs2501203
rs3807307
All
-0.10
-0.05
0.00
0.05
MR leave-one-out sensitivity analysis for
' || id:finn-b-M13_SYSTSLCE' on 'Interferon gamma levels || id:ebi-a-GCST004456'

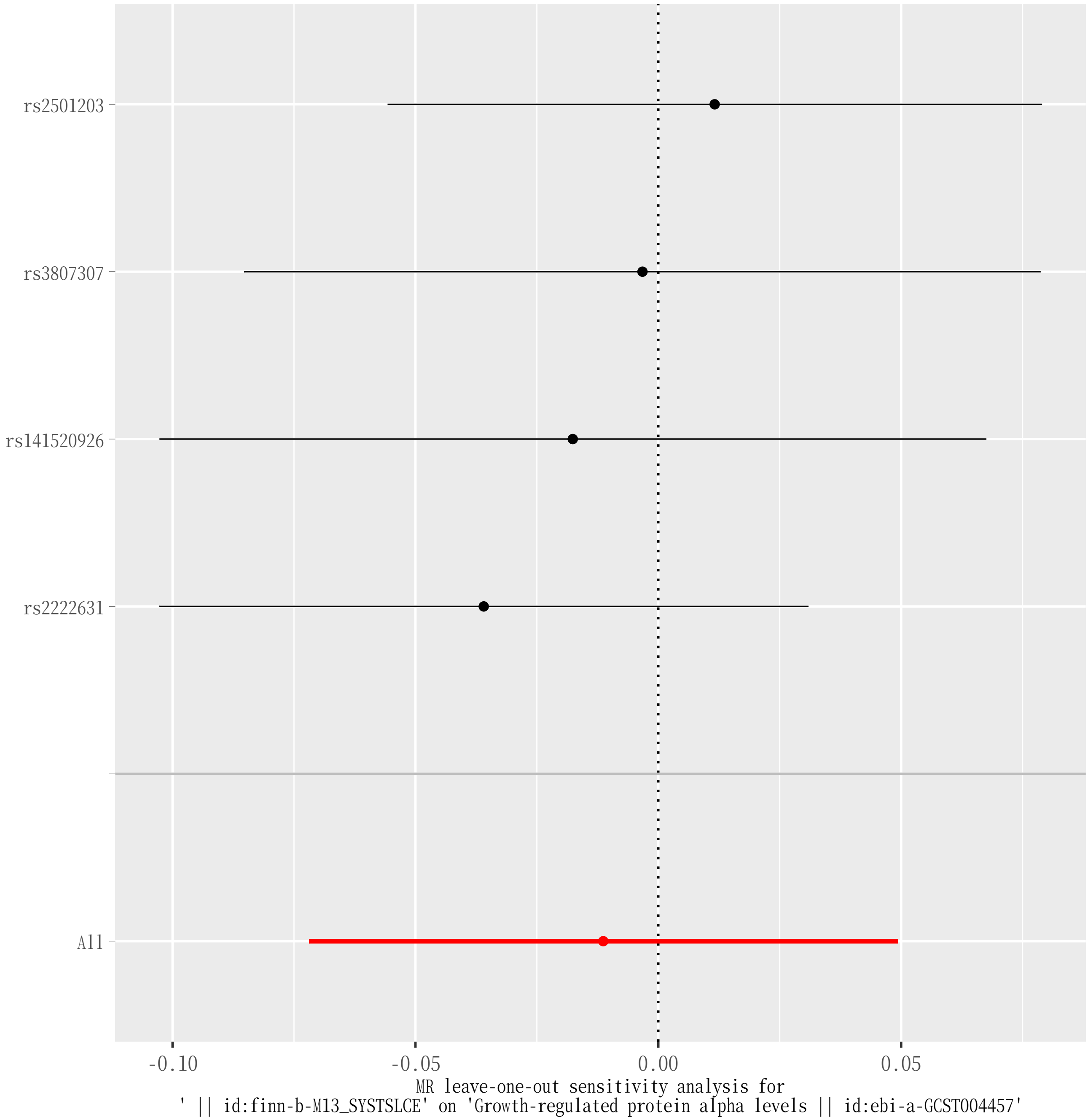
rs2501203
rs3807307
rs141520926
rs2222631
All
-0.10
-0.05
0.00
0.05
MR leave-one-out sensitivity analysis for
' || id:finn-b-M13_SYSTSLCE' on 'Growth-regulated protein alpha levels || id:ebi-a-GCST004457'

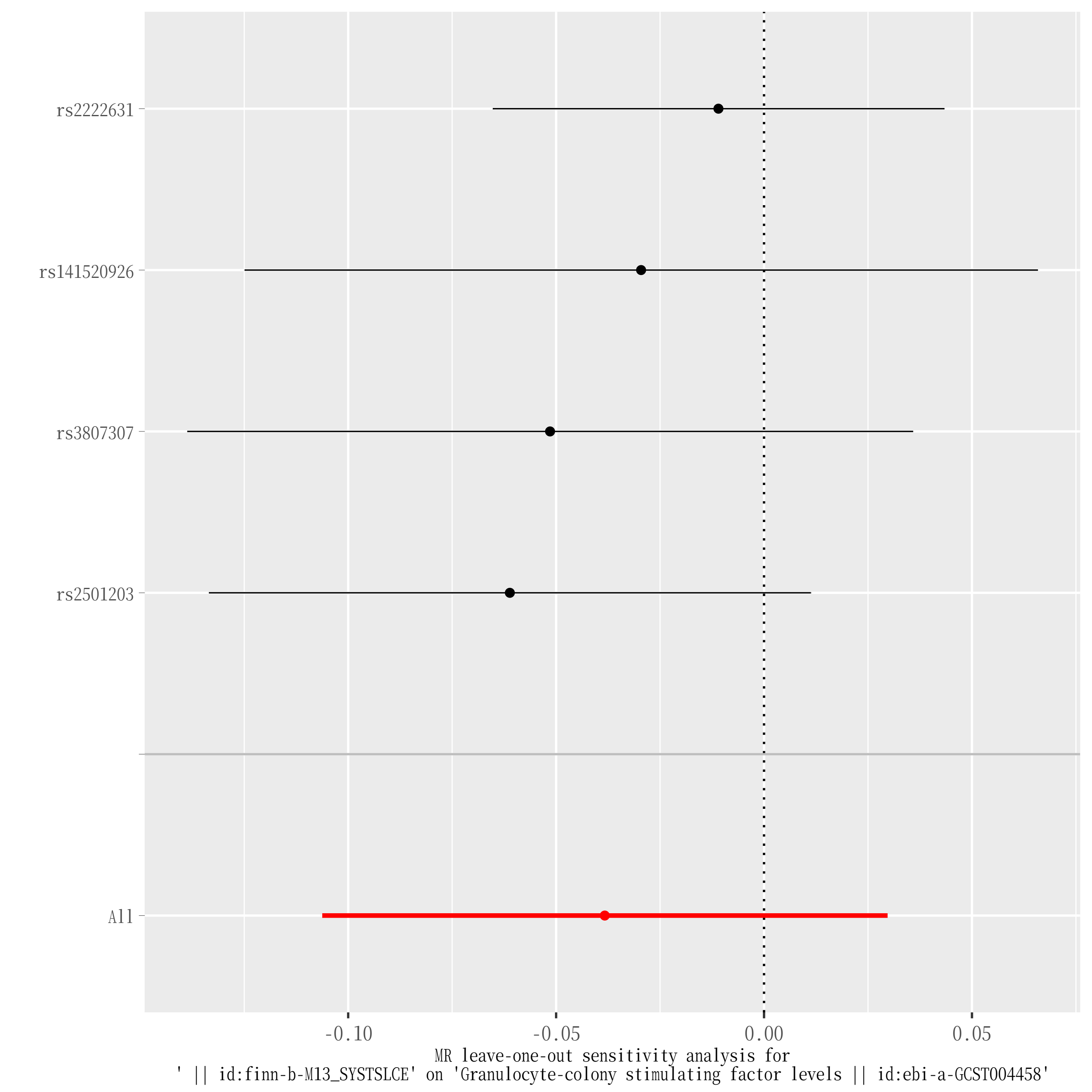
rs2222631
rs141520926
rs3807307
rs2501203
All
-0.10
-0.05
0.00
0.05
MR leave-one-out sensitivity analysis for
' || id:finn-b-M13_SYSTSLCE' on 'Granulocyte-colony stimulating factor levels || id:ebi-a-GCST004458'

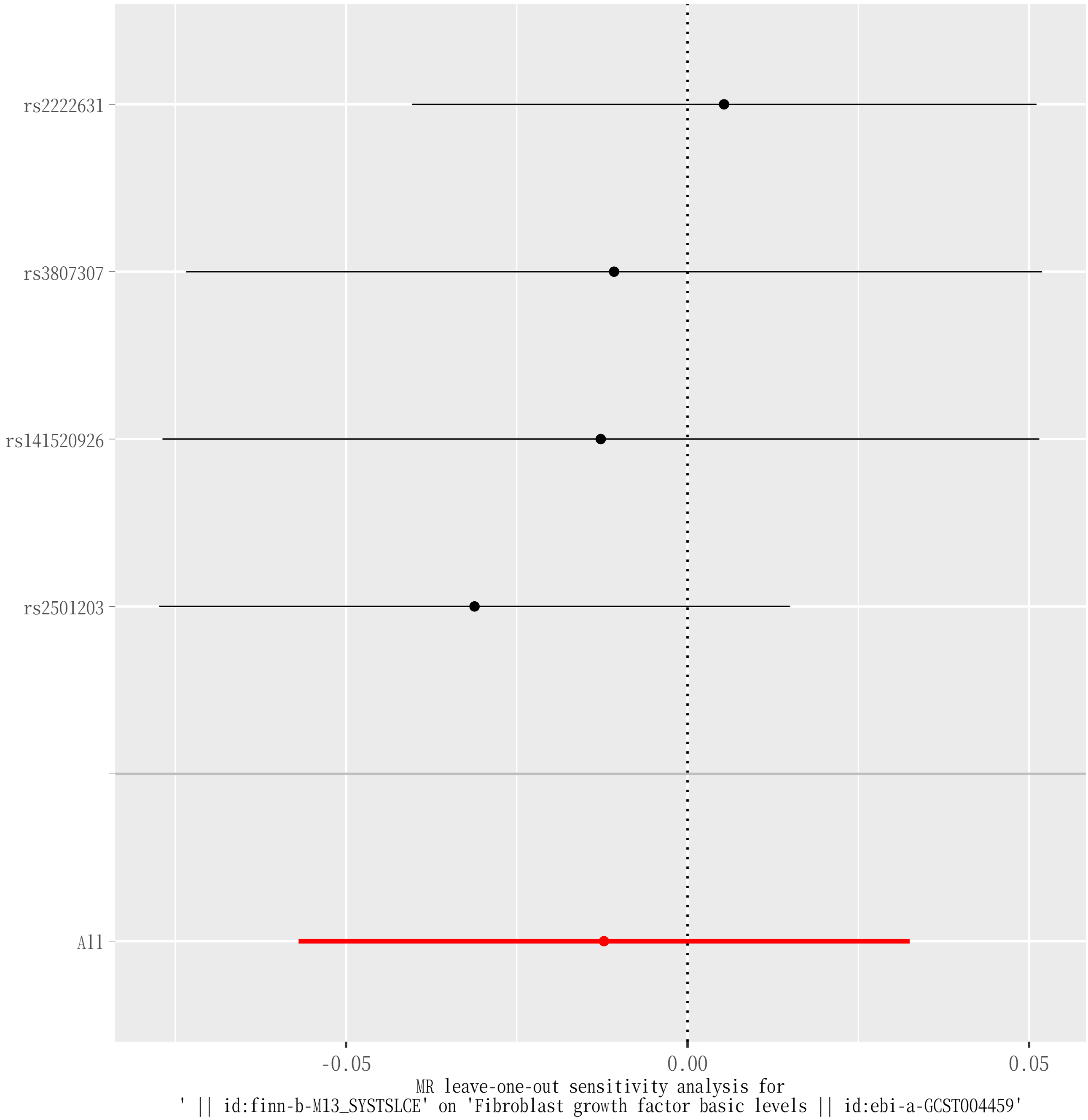

rs2222631
rs3807307
rs141520926
rs2501203
All
-0.05
0.00
0.05
MR leave-one-out sensitivity analysis for
' || id:finn-b-M13_SYSTSLCE' on 'Fibroblast growth factor basic levels || id:ebi-a-GCST004459'

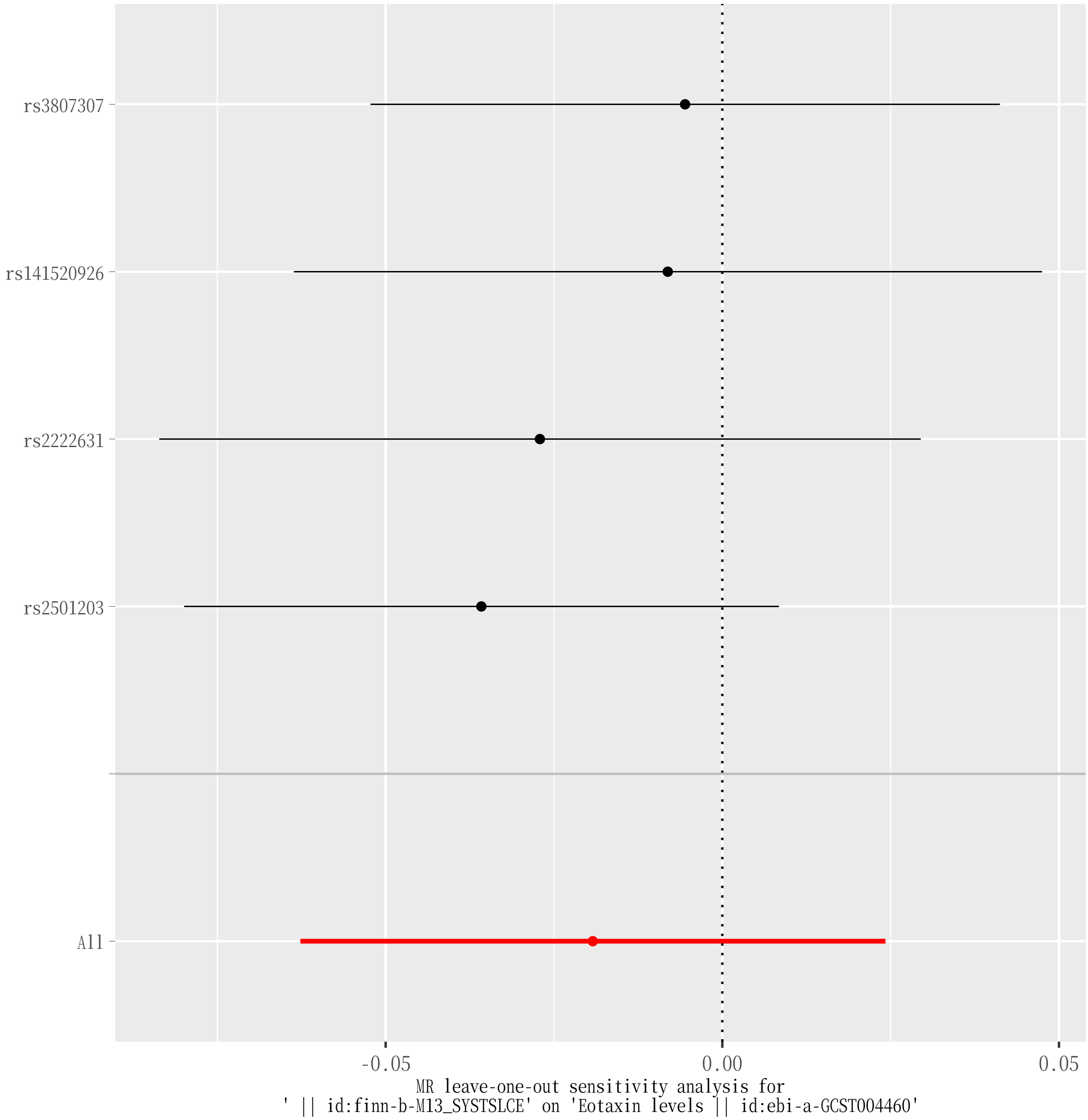
rs3807307
rs141520926
rs2222631
rs2501203
All
-0.05
0.00
0.05
MR leave-one-out sensitivity analysis for
' || id:finn-b-M13_SYSTSLCE' on 'Eotaxin levels || id:ebi-a-GCST004460'
